# Weaning differentially affects mitochondrial function, oxidative stress, inflammation and apoptosis in normal and low birth weight piglets

**DOI:** 10.1371/journal.pone.0247188

**Published:** 2021-02-19

**Authors:** Aliny K. Novais, Karine Deschêne, Yan Martel-Kennes, Caroline Roy, Jean-Paul Laforest, Martin Lessard, J. Jacques Matte, Jerome Lapointe

**Affiliations:** 1 Agriculture and Agri-Food Canada, Sherbrooke Research and Development Centre, Sherbrooke, Quebec, Canada; 2 Department of Animal Science, Universidade Estadual de Londrina, Londrina, Paraná, Brazil; 3 Département des Sciences Animales, Université Laval, Ville de Québec, Québec, Canada; INIA, SPAIN

## Abstract

Weaning is associated with increased occurrence of infections and diseases in piglets. Recent findings indicate that weaning induces mitochondrial dysfunction and oxidative stress conditions that more severely impact smaller piglets. The objective of this study was to characterize the molecular mechanisms underlying these physiological consequences and the relation with systemic inflammatory status in both normal and low birth weight (NBW and LBW) piglets throughout the peri-weaning period. To conduct the study, 30 sows were inseminated, and specific piglets from their litters were assigned to one of two experimental groups: NBW (n = 60, 1.73 ± 0.01 kg,) and LBW piglets weighing less than 1.2 kg (n = 60, 1.01 ± 0.01 kg). Then, 10 piglets from each group were selected at 14, 21 (weaning), 23, 25, 29 and 35 days of age to collect organ and plasma samples. Specific porcine RT^2^ Profiler™ PCR Arrays related to mitochondrial function, oxidative stress, inflammation and apoptosis processes were first used to target genes that are modulated after weaning in NBW piglets (d 23 and d 35 vs. d 14). Expression of selected genes was evaluated by quantitative PCR. These analyses revealed that expression of inflammatory genes *CXCL10* and *CCL19* increased after weaning in intestinal mucosa, while expression of genes encoding subunits of the mitochondrial respiratory chain was downregulated in liver and kidney of both groups. Interestingly, major modulators of mitophagy (*BNIP3*), cell survival (*BCL2A1*) and antioxidant defense system (*TXNRD2*, *GPx3*, *HMOX1*) were found to be highly expressed in NBW piglets. The systemic levels of TNF-α and IL1-β significantly increased following weaning and were higher in NBW piglets. These results provide novel information about the molecular origin of mitochondrial dysfunction and oxidative stress observed in weaned piglets and suggest that clearance of dysfunctional mitochondria, antioxidant defenses and inflammatory response are compromised in LBW piglets.

## Introduction

The separation of piglets from sows at weaning is known to induce many simultaneous stressors resulting in low feed intake, intestinal disturbances, inflammation and increased occurrence of diseases [1]. The post-weaning period is characterized by higher morbidity and mortality rates that cause substantial economic losses for producers. This is particularly true for low birth weight (LBW) piglets, which have become common in modern swine production with the advent of genetic selection for sows producing large litters [2, 3]. During the post-weaning period, these LBW piglets are recognized to have a lower feed intake than their normal birth weight (NBW) counterparts and to suffer from an incomplete development of both digestive and immune systems [4, 5]. These conditions result in an increase vulnerability of LBW piglets to bacterial infections and various pathologies known to be associated with the post-weaning period. This situation is highly problematic for swine producers which have to conform their practices with new and upcoming regulations regarding the utilisation of antibiotics and zinc oxide (ZnO) [6, 7]. As a result, alternatives to these products commonly used to promote health and growth of weaned piglets need to be developed.

It was convincingly demonstrated by several studies that oxidative stress conditions occur in weaned piglets whatever the weaning age [8–10]. Oxidative stress is normally defined as any imbalance between the generation of reactive oxygen species (ROS) and their neutralization by antioxidants. Several evidences indicate that resistance to diseases and integrity of intestinal barrier function could all be negatively affected by oxidative stress in mammals [11, 12]. In all cells, mitochondria play a crucial role in producing energy but are also the main sources of ROS as by-products of cellular respiration. Recent studies have clearly revealed that mitochondria constitute essential hubs controlling innate immune response [13], programmed cell death or apoptosis [14], inflammatory processes [15] and bacterial pathogenesis [16]. The links between mitochondrial energy metabolism, oxidative stress and development of adverse physiological outcomes might constitute important issues in swine production for animals facing high energetic demands [17]. Studies on the specific relation between mitochondrial function, oxidative stress and weaning stress are greatly limited but it was reported that the activities of mitochondrial respiratory complexes decrease in intestinal and hepatic tissues during the first week following weaning [9, 18]. Interestingly, we have recently demonstrated that mitochondrial dysfunction and cellular oxidative stress conditions are rapidly induced after weaning in piglets of 21 days of age and lasted for at least two weeks [19]. Results from this study also revealed that LBW piglets are affected by more severe mitochondrial energetic deficiencies and sustain higher oxidative damage than NBW piglets throughout the peri-weaning period.

Taken together, these results support the implication of mitochondrial dysfunction and oxidative stress conditions in the establishment of post-weaning stress, especially in LBW piglets. However, a more detailed analysis of the molecular mechanisms underlying these observed detrimental effects of weaning on mitochondria is needed in the optic of developing new molecular approaches to increase the robustness of weaning piglets and this is why we have decided to pursue their characterization. Mitochondria are at the interface between energetic substrate provided by the diet and cellular functions and an increasing number of evidence suggests that mitochondrial function can be modulated by nutrigenomics approaches [17]. In the same vein, the molecular consequences of weaning on the many physiological processes relying on mitochondrial function such as inflammation and programmed cell death also need to be better characterized throughout the peri-weaning period. Therefore, the objective of the present study is to provide for the first time a detailed molecular characterization of markers of mitochondrial energy metabolism, oxidative stress, inflammation and apoptosis in different tissues of both LBW and NBW piglets throughout a determined time period lasting from mid-lactation (day 14) until 2 weeks after weaning (day 35). This could contribute to establish the foundations for the development of novel targeted interventions aiming to improve health and resistance of weaned piglets.

## Materials and methods

### Animals

All animals involved in this research project have been treated in accordance with the code of good practice in effect (Agriculture and Agri-Food Canada, 1993) and all procedures involving these animals were approved by the Institutional Animal Care Committee of the Sherbrooke Research and Development Centre (CIPA 488) in accordance with Canadian Council of Animal Care guidelines on the care and use of farm animals in research [20]. The generation and the selection processes of the piglets involved in this study have been previously described [19]. Briefly, 30 conventional multiparous Yorkshire x Landrace sows were given the same feed allowances and diet formulations during gestation and lactation as previously described and had free access to water [21]. Litters were standardized to 12 piglets within 48 h of birth. The piglets were all weighed on day 1 and those presenting the required characteristics were assigned to one of two experimental groups: low birth weight piglets (LBW, n = 60) and normal birth weight piglets (NBW, n = 60). The selection criteria for NBW piglets was having a weight between the mean weight of the 360 produced piglets on d 1 and a standard deviation value (1.73 ± 0.01 kg) while the LBW piglets were selected among those presenting a body weight of less than 1.2 kg on d 1 (1.01 ± 0.01 kg) previously described and reported in the literature [19, 22]. The NBW and LBW piglets have been respectively selected from 25 and 26 out of the 30 litters. Both males and females were selected but they were carefully distributed within the two experimental groups (31 females and 29 males for LBW; 27 females and 30 males for NBW) as well as between the different sampling time points in order to avoid any confounding effect. Thus, litter and gender effects were not considered since the 120 piglets come from a total of 32 different litters and gender representation is fairly balanced between treatment groups and age. Piglets had no access to feed and water during the lactation period and were weaned on d 21. All the selected piglets were also weighted during lactation on days 1, 7, 14 and 21 (weaning day) as well as at 23, 25, 29 and 35 days of age. After weaning, previously identified LBW and NBW piglets were kept with their original littermates and raised in pens (one litter/pen) until weaning. Weaned piglets were fed ad libitum commercial weaning diets (3 phases feeding program containing high levels of ZnO) without antibiotics for the two weeks of the experimental period and had free access to drinking water. The analytical composition of these diets is described in S1 Table in S1 File. Within each group (LBW and NBW), 10 of the selected piglets were sacrificed on day 14, 21 (weaning), 23, 25, 29 and 35 days of age. Samples from the left lateral lobe of the liver and the left kidney were collected along with intestinal mucosal scrapings from the jejunum section. These samples were rinsed in phosphate-buffered saline (PBS), snap frozen in liquid nitrogen and stored at -80°C for future analyses. Blood samples were collected from 10 LBW and 10 NBW piglets on the corresponding day of sacrifice (days 14, 21, 23, 25, 29 and 35) as well as on day 22 for the piglets sacrificed on day 29. Blood samples were collected in EDTA tubes (Becton Dickinson and Company, Rutherford, NJ, USA) and were centrifuged within 20 min at 4°C for 12 min at 1800 x *g*. Plasma was immediately recovered and samples were frozen at -80°C until they were assayed.

### RNA extraction and cDNA synthesis

Total RNA was extracted from 50 mg of liver, intestinal mucosa and kidney samples from piglets of all ages using RNeasy spin columns (Qiagen, Mississauga, ON, Canada). The extracted RNA was dissolved in water and quantified by spectrophotometry using a NanoDrop ND-1000 (NanoDrop Technologies, Wilmington, DE, USA). An RNA aliquot was taken to verify its integrity using the Bioanalyzer RNA 6000 Nano assay (Agilent Technologies. Mississauga, ON, Canada). RNA integrity number (RIN) index was used as a numerical assessment of the integrity of RNA and only samples with a RIN greater than 9 were kept for further analyses. For each sample, 1 μg of total RNA was treated with DNase I (Life Technologies) to remove contaminating genomic DNA and first-strand cDNA was synthesized using either the RT^2^ First Strand Kit (Qiagen) for array analyses or SuperScript™ IV First-Strand Synthesis System (Life Technologies) for quantitative real-time PCR amplifications of specific genes.

### RT^2^ profiler™ qPCR arrays

Specific RT^2^ profiler PCR Array (Qiagen, Mississauga, ON, Canada) were used to asses the expression of porcine genes involved in mitochondrial energy metabolism (PASS-008Z-24), oxidative stress (PASS-065Z-24), apoptosis (PASS-012Z-24) and cytokines and chemokines (PASS-150ZC-24) in intestinal mucosa samples. For liver, only mitochondrial energy metabolism and oxidative stress arrays were performed. In order to profile the expression of genes involved in these processes throughout the peri-weaning period, these specific arrays were conducted on previously synthetized CDNA from intestinal and liver samples from NBW piglets of 14, 23 and 35 days of age following instructions provided by the manufacturer. Using SYBR Green ROX qPCR Mastermix (Qiagen), the samples were run on an Applied Biosystems™ 7500 Fast Real-Time PCR System (Applied Biosystems, Carlsbad, CA). Each array screened for 84 pathway-focussed porcine genes and five housekeeping genes and the data obtained were analyzed with the online Qiagen analysis software (RT^2^ profiler PCR array data analysis V3.5). Expression of genes that required more than 35 cycles to reach the threshold was determined as below the detection limit and discarded. Relative gene expression for days 23 and 35 were expressed as fold changes on a log2 scale compared to the expression on day 14 using the 2^-ΔΔCt^ method. Only individual pairwise comparisons were performed (d23 and d35 against d14) and not any comparison across multiple groups at the same time. Statistically significant differences between piglets from different ages were first calculated using a student-t-test on the log-transformed values of the expression normalized for the selected reference genes. Observed raw probabilities were also adjusted for multiple testing using False Discovery Rate in the MULTTEST procedure of SAS. A log2 fold change of 1 represents a two fold increase in gene expression, whereas a log2 fold change of -1 represents a two fold reduction in expression. The complete results obtained with these profiler arrays are provided in S3-S8 Tables in S1 File and all raw data are available in S9-S14 Tables in S1 File.

### Quantitative RT-PCR

The relative mRNA abundance of targeted genes in liver, intestinal mucosa and kidney was determined using real time PCR amplifications on an Applied Biosystems™ 7500 Fast Real-Time PCR System (Applied Biosystems, Foster City, CA, USA). The genes specific to liver and intestinal mucosa were selected based on arrays results and careful review of the literature. The genes related to the kidney were selected based on the array results obtained with liver samples as well as the scientific literature. Primers were designed using the Primer Express software 3.0 (Applied Biosystems) according to the National Center for Biotechnology Information (NCBI) database. Primer sequences for studied and reference genes are shown in S2 Table in S1 File. Quantitative real-time PCR were performed in a 10-μL reaction volume using Power SYBR-Green Master Mix (PE Applied BioSystems) with the following cycling conditions: 10 min at 95°C followed by 40 cycles at 95°C for 15 s and at 60°C for 45 s. Specificity of amplified fragments was verified with the melting curve analysis (Dissociation Curves v1.0; PE Applied Biosystems). Amplifications were performed in triplicate. The cycle threshold (Ct), the number of cycles required for the fluorescent signal to cross the threshold, was determined for all studied and reference genes. The relative expression of each gene was then calculated using 2^−ΔCt^, where ΔCt is the difference between the Ct of the genes of interest and the reference genes which were the least affected by treatment according to the NormFinder statistical algorithm. These reference genes are *RPL4* (*Ribosomal Protein L4*) and *RPL13* (*Ribosomal Protein L13A*) for intestinal mucosa samples and liver samples as well as *B2M* (*beta-2-Microglobulin*) and *GAPDH* (*Glyceraldehyde-3-Phosphate Dehydrogenase*) for kidney samples.

### Cytokines assays

Quantification of pro-inflammatory cytokines as tumor necrosis factor-α (TNF-α) and interleukin-1β (IL-1β) were assayed in duplicate in plasma samples using commercial porcine ELISA kits according to the protocols provided by the manufacturer R&D Systems. Briefly, standard curves were made using seven serial dilutions of the recombinant porcine cytokine for TNF-α were 2000 pg/ml and 4000 pg/ml for IL-1β for the first point, respectively. The readings were measured by a spectrophotometer at an OD of 450 nm and the correction made at 540 nm. The intra-assay coefficients of variation for TNF‐α and IL-1β were 3.1% and 3.8% respectively, and the inter-assay coefficients of variation for TNF‐α and IL-1β were 3.38% and 4.6%, respectively. The results were expressed in picograms per millilitre based on a standard curve.

### Statistical analysis

Data were analysed using the MIXED procedure of SAS (SAS release 9.2, 2002, SAS Institute, Cary, NC, USA). Analysis of variance was performed according to a completely randomized design to test the effects of the two studied factors (birth weight, age of piglets) and their interaction on mRNA expression and cytokines expression levels. Piglets were used as experimental units. The usual model for a complete randomized design used in this experiment is: *Y*_*ijk*_ = *μ* + *t*_*I*_ + *γ*_*j*_ + *(tγ)*_*ij*_ + *e*_*ijk*,_ where *Y*_*ijk*_ is the observation for animal *k* in birth weight group *i* at age *j*, *μ* is the overall mean, *t*_*i*_ is the fixed effect of birth weight (*i* = LBW of NBW), *γ*_*j*_ is the fixed effect of age *j* of piglet, *tγ*_*ij*_ is the interaction between birth weight and age and *e*_*ijk*_ is the residual error. Litter and gender effects were included in a separate comparative analysis as random factors in the previous model. Multiple comparisons were performed with a Tukey’s adjustment to compare ages. Effects of the statistical model were considered significant at P ≤ 0.05, and the tendency (trend) at 0.05 < P ≤ 0.10.

## Results

### Expression of mitochondrial energy metabolism and oxidative stress-associated genes in liver of pre- and post-weaned NBW piglets

The mean body weight of these piglets was 1.73 ± 0.01 kg at d 1 and they were 42% heavier than the LBW piglets. The weight gains for both groups were modest during the first days of the post-weaning period and the weight difference between them remains until d 35 (S1 Fig in S1 File). The decision to target the NBW piglets and to specifically use the mitochondrial energy metabolism and oxidative stress arrays is based on previous results showing that energy production and antioxidant response are compromised in liver of both LBW and NBW piglets. Thus, by performing these PCR arrays, we found that the number of differently expressed genes, compared to d 14, was greater two weeks after weaning at d 35 days than at d 23 (Tables 1 and 2). Almost all the differentially expressed genes in liver identified by performing student t-tests using the RT2 profiler PCR array data analysis software were found to be down-regulated. Among the ones related to the mitochondrial energy metabolism, the majority were genes encoding subunits of the mitochondrial respiratory chain such as the NADH dehydrogenase, succinate dehydrogenase and ATP synthase complexes. However, when adjusted for false discovery rate (FDR) for multiple comparisons, we observed that the expression of only one subunit of the NADH dehydrogenase (NDUFA1) tended to differ between d 23 and d 14 (Table 1). Similarly, the subunit ATP6V1G3 of ATP synthase complex was the one which tended to be differently expressed between d 35 and d 14 (Table 2). Results from the oxidative stress arrays indicated that only a few genes encoding proteins implicated in the antioxidant response tended to be differentially expressed between pre- and post-weaning periods after adjustment for FDR. These ones are arachidonate 12-Lipoxygenase 12S Type (ALOX12), albumin (ALB), metallothionein III (MT-III) as well as members of the thioredoxin systems (*TXN* and *TXNRD2*). The complete results obtained with these profiler arrays are provided in S3 and S4 Tables in S1 File.

**Table 1:** Differently expressed genes identified by RT^2^-profilers arrays in the liver of 23 versus 14 days old Normal Birth Weight (NBW) piglets.

Arrays/ Genes^a^	Description	Fold^b^	p-values^c^	FDR^d^
**Oxidative stress**				
***ALB***	Albumin	-2.60	0.040	0.553
***ALOX12***	Arachidonate 12-lipoxygenase	-5.82	0.013	0.553
***PRNP***	Prion protein	-2.17	0.046	0.553
**Mito. energy**				
***ATP5A1***	ATP synthase, H+ transporting, mitochondrial F1 complex, alpha subunit 1	-2.69	<0.01	0.165
***ATP6V1G3***	V-type proton ATPase subunit G 3-like	-3.52	<0.01	0.086
***NDUFB6***	NADH dehydrogenase 1 beta subcomplex 6	-2.55	0.020	0.216
***NDUFA1***	NADH dehydrogenase [ubiquinone] 1 alpha subcomplex subunit 1-like	-2.31	<0.01	0.074
***NDUFB7***	NADH dehydrogenase (ubiquinone) 1 beta subcomplex, 7, 18kDa	-6.55	0.020	0.216
***NDUFS2***	NADH dehydrogenase (ubiquinone) (NADH-coenzyme Q reductase)	-6.47	0.044	0.254
***SDHB***	Succinate dehydrogenase complex, subunit B, iron sulfur	-3.18	0.026	0.223

^a^RT^2^-profilers used were oxidative stress and mitochondrial energy metabolism (mito. energy) and each array included 84 genes.

^b^Fold change (2^-ΔΔCt^) is the normalized gene expression (2^-ΔCt^)) at d 23 divided the normalized gene expression (2^-ΔCt^) at d 14. Fold-change values greater than one indicates a positive- or an up-regulation and values less than one indicate a negative or down-regulation.

^c^The p values are calculated based on a Student’s t-test of the replicate (2^-ΔCt^) values for each gene from NBW piglets of both ages.

^d^The FDR is the False Discovery Rate adjusted p-values.

**Table 2:** Differently expressed genes identified by RT^2^-profilers arrays in the liver of 35 versus 14 days old Normal Birth Weight (NBW) piglets.

Arrays/ Genes^a^	Description	Fold^b^	p-values^c^	FDR^d^
**Oxidative stress**				
***ALB***	Albumin	-3.78	<0.01	0.037
***ALOX12***	Arachidonate 12-lipoxygenase	-5.02	<0.01	0.083
***APOE***	Apolipoprotein E	-2.26	<0.01	0.99
***CCL5***	Chemokine (C-C motif) ligand 5	-2.24	<0.01	0.083
***FOXM1***	Forkhead box M1	-3.62	0.049	0.188
***GPX3***	Glutathione peroxidase 3 (plasma)	-4.57	0.015	0.126
***MT-III***	Metallothionein-III	-5.96	<0.01	0.068
***PTGR1***	Prostaglandin reductase 1	3.61	0.020	0.158
***TTNLOC100620261***	Titin	-37.57	0.038	0.183
***TXN***	Thioredoxin	2.80	0.012	0.107
***TXNRD2***	Thioredoxin reductase 2	-2.06	<0.01	0.083
**Mito. energy**				
***ATP6V1C2***	ATPase, H+ transporting, lysosomal 42kDa, V1 subunit C2	-3.49	0.031	0.156
***ATP6V1G3***	V-type proton ATPase subunit G 3-like	-2.92	<0.01	0.087
***DNAJB1***	DnaJ (Hsp40) homolog, subfamily B, member 1	-2.93	0.035	0.156
***HSP70*.*2***	Heat shock protein 70.2	-6.42	0.015	0.141
***LOC100154992***	Mitochondrial inner membrane protein OXA1L-like	-2.84	0.035	0.156
***NDUFA10***	NADH dehydrogenase [ubiquinone] 1 alpha subcomplex subunit 10, mitochondrial-like	-2.18	0.037	0.156
***NDUFB7***	NADH dehydrogenase (ubiquinone) 1 beta subcomplex, 7, 18kDa	-88.59	0.012	0.135
***NDUFS2***	NADH dehydrogenase (ubiquinone) (NADH-coenzyme Q reductase)	-16.72	0.036	0.156
***NDUFS3***	NADH dehydrogenase (ubiquinone) (NADH-coenzyme Q reductase)	-2.28	0.019	0.149
***NDUFS7***	NADH dehydrogenase [ubiquinone] iron-sulfur protein 7, mitochondrial-like	-2.02	0.038	0.156
***SDHA***	Succinate dehydrogenase [ubiquinone] flavoprotein subunit, mitochondrial	-2.68	0.036	0.156
***SDHB***	Succinate dehydrogenase complex, subunit B, iron sulfur (Ip)	-2.94	0.030	0.156

^a^RT^2^-profilers used were oxidative stress and mitochondrial energy metabolism (mito. energy) and each array included 84 genes.

^b^Fold change (2^-ΔΔCt^) is the normalized gene expression (2^-ΔCt^)) at d 23 divided the normalized gene expression (2^-ΔCt^) at d 14. Fold-change values greater than one indicates a positive- or an up-regulation and values less than one indicate a negative or down-regulation.

^c^The p values are calculated based on a Student’s t-test of the replicate (2^-ΔCt^) values for each gene from NBW piglets of both ages.

^d^The FDR is the False Discovery Rate adjusted p-values.

### Expression of mitochondrial energy metabolism, oxidative stress, inflammation and apoptosis-associated genes in intestinal mucosa of pre- and post-weaned NBW piglets

As for the liver, intestinal mucosa samples from NBW piglets of 23 and 35 days of age were used to identify genes associated to mitochondrial energy metabolism and oxidative stress that are differentially expressed between the pre and post-weaning period. We first observed that the expression of only 3 genes related to mitochondrial energy metabolism, subunits of ATPase and NADH dehydrogenase, was modulated between d 14 and the post-weaning period and only *ATP4B* remained significant following FDR adjustement for multiple comparisons (Tables 3 and 4). In the oxidative stress PCR array, we found that several genes encoding enzymes implicated in the antioxidant response were highly expressed at d 35 when compared to d 14 in the lactation period. The expression of the FTH1 gene is significantly higher at d 35 while HMOX1, SIRT2 and genes related to the glutathione system (*GPx2 andGPx4*) tended to be highly expressed at d 35 (Table 4) systems. We performed the cytokines and chemokines PCR array and we observed that many genes encoding inflammatory factors such as members of CC and CXC chemokines families as well as some interleukins are up-regulated in intestinal mucosa of NBW piglets at both d 23 and d 35 in comparison to d 14 (Tables 3 and 4). The apoptosis PCR array was initially chosen because of the established link between programmed cell death and mitochondrial dysfunction. Defects in mitochondrial energy metabolism have previously been observed in both NBW and LBW post-weaned piglets. Results from this specific array indicate that only one gene associated to apoptosis was induced at d 23. More genes implicated in programmed-cell death and cell survival were found to be differentially expressed two weeks after weaning at d 35 (Tables 3 and 4). The complete results obtained with these profiler arrays are provided in S5-S8 Tables in S1 File.

**Table 3:** Differently expressed genes identified by RT^2^-profilers arrays in the intestinal mucosa of 23 versus 14 days old Normal Birth Weight (NBW) piglets.

Arrays/ Genes^a^	Description	Fold^b^	p-values^c^	FDR^d^
**Oxidative stress**				
***GP91-PHOX***	NADPH oxidase heavy chain subunit	5.92	<0.01	0.289
***GPX1***	Glutathione peroxidase 1	2.51	0.023	0.326
***LOC100049683***	Solute carrier family 7, member 11	2.84	0.046	0.336
***LOC100739163***	Glutathione S-transferase P	2.26	0.047	0.336
***MPO***	Myeloperoxidase	24.58	0.010	0.289
***NCF1***	Neutrophil cytosolic factor 1	5.04	0.017	0.290
***TXNRD2***	Thioredoxin reductase 2	2.31	0.031	0.336
**Mito. energy**				
***ATP6V0D2***	ATPase, H+ transporting, lysosomal 38kDa, V0 subunit d2	22.45	0.019	0.451
***ATP6V1E2***	ATPase, H+ transporting, lysosomal 31kDa, V1 subunit E2	8.50	0.038	0.451
***NDUFB7***	NADH dehydrogenase (ubiquinone) 1 beta subcomplex, 7, 18kDa	11.93	0.033	0.451
**Cytokines**				
***CCL1***	CCL1	8.53	<0.01	<0.01
***CCL19***	Chemokine (C-C motif) ligand 19	7.93	<0.01	<0.01
***CCL22***	C-C motif chemokine 22-like	9.30	0.021	0.070
***CCL8***	Chemokine (C-C motif) ligand 8	5.21	<0.01	<0.01
***CSF3***	Colony stimulating factor 3 (granulocyte)	3.35	<0.01	<0.01
***IFNB1***	Interferon beta	14.52	<0.01	<0.01
***IL12A***	Interleukin 12A (natural killer cell stimulatory factor 1)	9.60	<0.01	0.016
***IL12B***	Interleukin 12B (natural killer cell stimulatory factor 2)	33.47	0.035	0.104
***IL13***	Interleukin 13	4.74	0.014	0.057
***IL16***	Interleukin 16	2.57	<0.01	0.016
***IL17A***	Interleukin 17A	2.09	<0.01	<0.01
***IL4***	Interleukin 4	2.93	0.016	0.064
***IL6***	Interleukin 6 (interferon, beta 2)	10.14	<0.01	<0.01
***LTA***	Lymphotoxin alpha (TNF superfamily, member 1)	22.81	<0.01	0.029
***LTB***	Lymphotoxin beta (TNF superfamily, member 3)	10.71	<0.01	<0.01
***TGFB1***	Transforming growth factor, beta 1	3.68	<0.01	0.041
***TNFSF13B***	Tumor necrosis factor (ligand) superfamily, member 13b	11.50	<0.01	<0.01
**Apoptosis**				
***BCL2A1***	Apoptosis-associated speck-like protein containing a CARD-like	5.27	0.049	0.352
***CD40***	CD40 ligand	4.82	<0.01	0.029

^a^RT^2^-profilers used were oxidative stress, mitochondrial energy metabolism (mito. energy), cytokines and chemokines (cytokines) and apoptosis. Each array included 84 genes.

^b^Fold change (2^-ΔΔCt^) is the normalized gene expression (2^-ΔCt^)) at d 23 divided the normalized gene expression (2^-ΔCt^) at d 14. Fold-change values greater than one indicates a positive- or an up-regulation and values less than one indicate a negative or down-regulation.

^c^The p values are calculated based on a Student’s t-test of the replicate (2^-ΔCt^) values for each gene from NBW piglets of both ages.

^d^The FDR is the False Discovery Rate adjusted p-values.

**Table 4:** Differently expressed genes identified by RT^2^-profilers arrays in the intestinal mucosa of 35 versus 14 days old Normal Birth Weight (NBW) piglets.

Arrays/Genes^a^	Description	Fold^b^	p-values^c^	FDR^d^
**Oxidative stress**				
**AOX1**	Aldehyde oxidase-like	-2.17	0.017	0.120
**CCL5**	Chemokine (C-C motif) ligand 5	2.10	<0.01	0.049
**FTH1**	Ferritin, heavy polypeptide 1	-2.48	<0.01	0.031
**GPX2**	Glutathione peroxidase 2 (gastrointestinal)	4.82	<0.01	0.065
**GPX4**	Glutathione peroxidase 4 (phospholipid hydroperoxidase)	-2.19	<0.01	0.058
**HMOX1**	Heme oxygenase (decycling) 1	-5.31	0.036	0.106
**SFTPD**	Surfactant protein D	2.57	0.023	0.130
**SIRT2**	Sirtuin 2	-2.19	<0.01	0.058
**UCP2**	Uncoupling protein 2 (mitochondrial, proton carrier)	-2.62	0.046	0.186
**Mito. energy**				
**ATP4B**	ATPase, H+/K+ exchanging, beta polypeptide	9.00	<0.01	<0.01
**EDN1**	Endothelin 1	-2.21	0.025	0.323
**NDUFB4**	NADH dehydrogenase [ubiquinone] 1 beta subcomplex subunit 4-like	-2.27	<0.01	0.168
**Cytokines**				
**BMP3**	Bone morphogenetic protein 3	-2.58	<0.01	0.024
**C5**	Complement component 5	2.42	<0.01	<0.01
**CCL19**	Chemokine (C-C motif) ligand 19	2.38	0.015	0.026
**CCL21**	Chemokine (C-C motif) ligand 21	-2.98	0.028	0.078
**CCL28**	Chemokine (C-C motif) ligand 28	3.37	0.027	0.078
**CXCL10**	Chemokine (C-X-C motif) ligand 10	6.36	<0.01	<0.01
**CXCL9**	Chemokine (C-X-C motif) ligand 9	8.18	<0.01	0.026
**IFN-ALPHA-4**	Interferon-alpha-4	-2.69	<0.01	<0.01
**IFN-ALPHA-5**	Interferon, alpha 5	-2.06	<0.01	0.013
**IL12B**	Interleukin 12B (natural killer cell stimulatory factor 2, cytotoxic lymphocyte maturation factor 2, p40)	10.41	<0.01	0.018
**IL17A**	Interleukin 17A	4.68	<0.01	<0.01
**IL17F**	Interleukin 17F	5.66	0.028	0.078
**IL18**	Interleukin 18 (interferon-gamma-inducing factor)	2.10	<0.01	0.019
**IL2**	Interleukin 2	18.86	0.021	0.067
**IL21**	Interleukin 21	264.39	<0.01	<0.01
**IL22**	Interleukin 22	10.10	0.022	0.067
**INHBA**	Inhibin, beta A	-2.98	<0.01	<0.01
**CNTF**	Ciliary neurotrophic factor-like	-2.27	<0.01	0.026
**TNFSF13B**	Tumor necrosis factor (ligand) superfamily, member 13b	29.53	<0.01	<0.01
**Apoptosis**				
**APAF1**	Apoptotic peptidase activating factor 1	-2.58	0.044	0.104
**BCL2A1**	Apoptosis-associated speck-like protein containing a CARD-like	2.94	<0.01	0.015
**LOC100154044**	BCL2-like 2	-2.83	0.029	0.093
**CASP1**	Caspase 1, apoptosis-related cysteine peptidase (interleukin 1, beta, convertase)	2.09	<0.01	0.014
**CASP10**	Caspase 10, apoptosis-related cysteine peptidase	-2.06	0.012	0.046
**CIDEB**	Cell-death-inducing DNA-fragmentation-factor-like effector B	-2.63	<0.01	0.014
**IGF1R**	Insulin-like growth factor 1 receptor	-4.88	<0.01	0.022
**LOC100522011**	Apoptosis-associated speck-like protein containing a CARD-like	2.09	<0.01	0.012
**LTA**	Lymphotoxin alpha (TNF superfamily, member 1)	-4.11	0.042	0.101
**MTL5**	Metallothionein-like 5, testis-specific (tesmin)	2.24	0.032	0.093

^a^RT^2^-profilers used were oxidative stress, mitochondrial energy metabolism (mito. energy), cytokines and chemokines (cytokines) and apoptosis. Each array included 84 genes.

^b^Fold change (2^-ΔΔCt^) is the normalized gene expression (2^-ΔCt^)) at d 23 divided the normalized gene expression (2^-ΔCt^) at d 14. Fold-change values greater than one indicates a positive- or an up-regulation and values less than one indicate a negative or down-regulation.

^c^The p values are calculated based on a Student’s t-test of the replicate (2^-ΔCt^) values for each gene from NBW piglets of both ages.

^d^The FDR is the False Discovery Rate adjusted p-values.

### Expression of targeted genes in liver of both LBW and NBW piglets throughout the peri-weaning period

**Fig 1:**
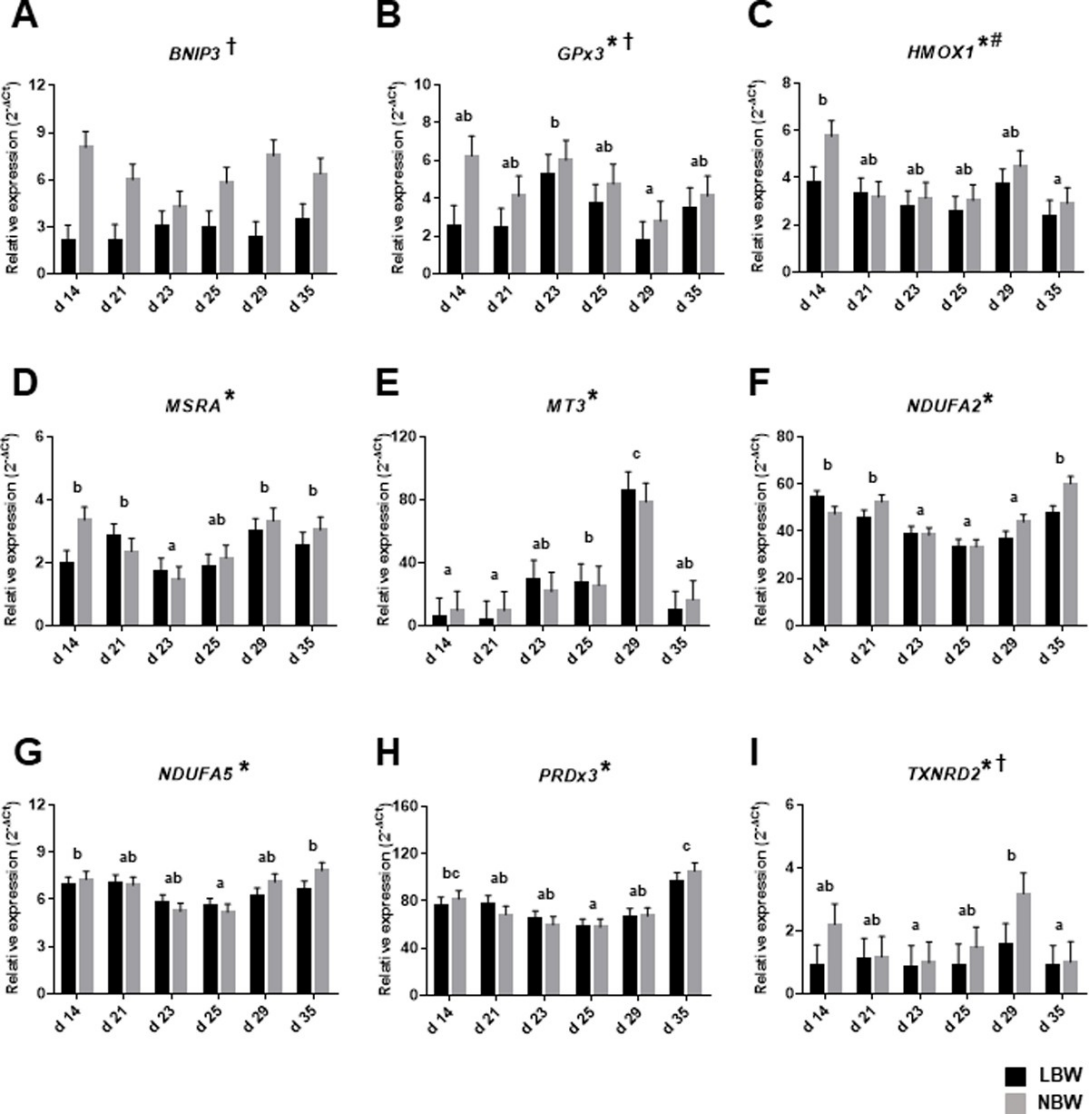
Relative mRNA levels of *BNIP3*, *GPx3*, *HMOX1*, *MRSA*, *MT3*, *NDUFA2*, *NDUFA5*, *PRDx3* and *TXNRD2* were analyzed in liver of LBW and NBW piglets of different ages throughout the peri-weaning period. Bars in the graphs represent least-square means ± SEM of 10 piglets. Asterisk (*) indicates a significant effect of the age on means at P ≤ 0.05. Dagger (†) indicates a significant effect of the birth weight on means at P ≤ 0.05. Pound (#) indicates that means tended to be affected by birth weight at P < 0.10. In all cases, the interaction between age and birth weight is not significant P > 0.05. Detailed *P-Values* for main effects and interactions are available in S15 Table in S1 File and comparative analysis with litter and gender as random cofactors in S16 Table in S1 File and box plot of raw data distribution in S3 Fig in S1 File. Ages with different letters (a, b, c, d) within the graphs are statistically different at P ≤ 0.05. LBW = low-birth weight piglets; NBW = normal-birth weight piglets; (A) *BNIP3* = BCL2 interacting protein 3; (B) *GPx3* = glutathione peroxidase 3; (C) *HMOX1* = heme oxygenase 1; (D) *MRSA* = methionine sulfoxide reductase A, (E) *MT3* = metallothionein 3; (F) *NDUFA2* = NADH ubiquinone oxidoreductase subunit A2; (G) *NDUFA5* = NADH ubiquinone oxidoreductase subunit A5; (H) *PRDx3* = peroxideroxin 3; (I) *TXNRD2* = thioredoxin reductase 2.

We have analyzed the expression of specific genes throughout the peri-weaning period in liver samples of LBW and NBW piglets. Results from these analyses first indicated that genes encoding *BNIP3*, known to be implicated in degradation of defective mitochondria, the mitochondrial antioxidant thioredoxin reductase 2 (*TXNRD2*) and the antioxidant enzyme *GPx3* are more expressed in NBW than LBW piglets (Fig 1A, 1B and 1I). We also observed that the expression of heme oxigenase 1 (*HMOX1*), methionine sulfoxide reductase system A (*MSRA*) and peroxiredoxin 3 (*PRDx3*) is modulated during the peri-weaning period (Fig 1C, 1D and 1H). Finally, the mRNA expression of subunits of the NADH dehydrogenase, *NDUFA2* and *NDUFA5*, is also significantly modulated throughout the peri-weaning period. We noted that the expression of these genes decreased sharply after weaning before increasing at d 35 (Fig 1F and 1G).

### Expression of targeted genes in intestinal mucosa of both LBW and NBW piglets throughout the peri-weaning period

**Fig 2:**
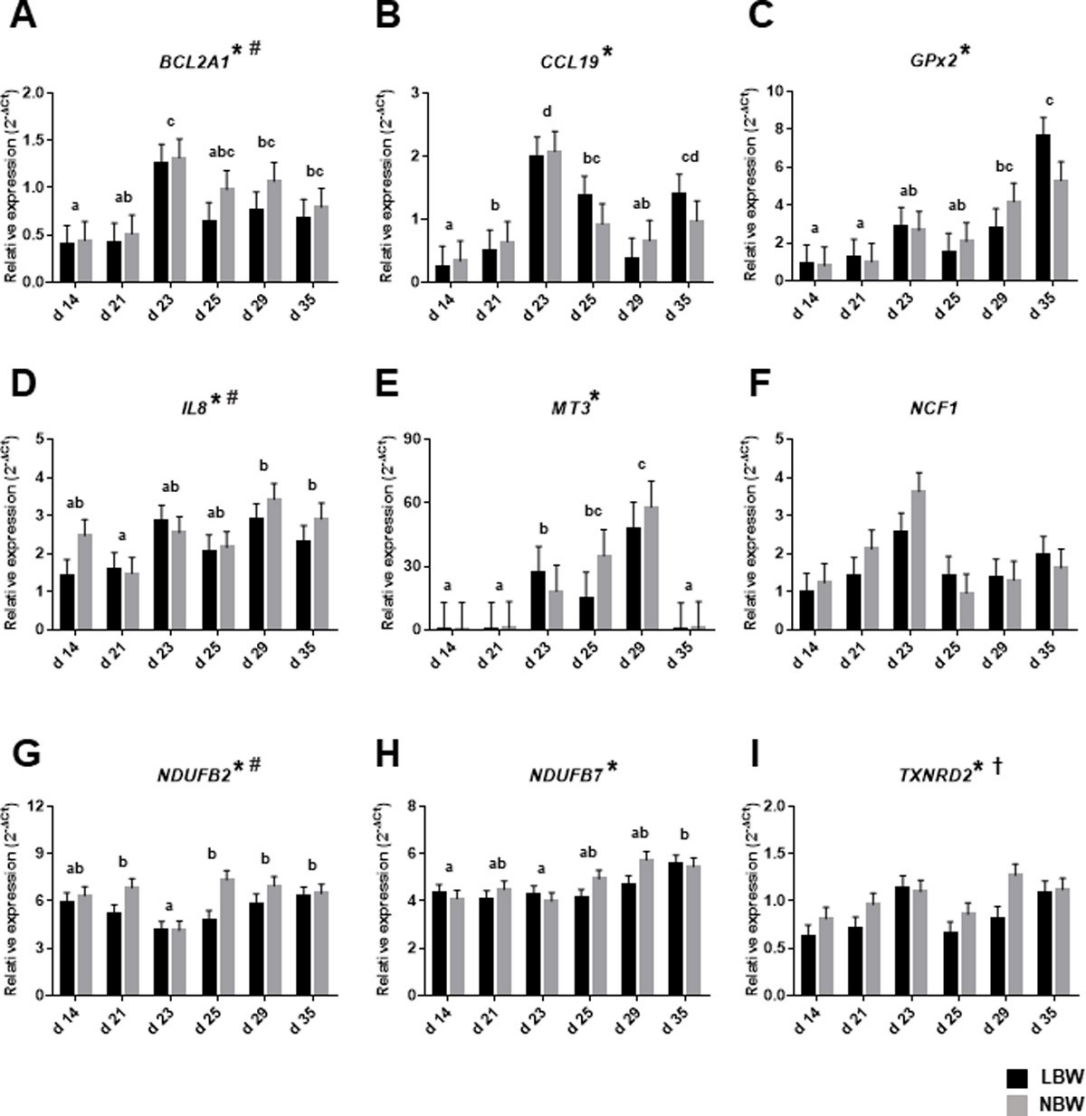
Relative mRNA levels of *BCL2A1*, *CCL19*, *GPx2*, *IL8*, *MT3*, *NCF1*, *NDUFB2*, *NDUFB7* and *TXNRD2* were analyzed in mucosa of the jejunum of from the two experimental groups of piglets from different ages throughout the peri-weaning period. Bars in the graphs represent least-square means ± SEM of 10 piglets. Asterisk (*) indicates a significant effect of the age on means at P ≤ 0.05. Dagger (†) indicates a significant effect of the birth weight on means at P ≤ 0.05. In all cases, the interaction between age and birth weight is not significant P > 0.05. Pound (#) indicates that means tended to be affected by birth weight at P < 0.10. Detailed *P-Values* for main effects and interactions are available in S15 Table in S1 File and comparative analysis with litter and gender as random cofactors in S16 Table in S1 File and box plot of raw data distribution in S4 Fig in S1 File. Ages with different letters (a, b, c, d) within the graphs are statistically different at P ≤ 0.05. LBW = low-birth weight piglets; NBW = normal-birth weight piglets; (A) *BCL2A1* = B-cell lymphoma 2-related protein A1 (B) *CCL19* = c-c motif chemokine ligand 19; (C) *GPx2* = glutathione peroxidase 2; (D) *IL8* = interleukin 8; (E) *MT3* = metallothionein 3; (F) *NCF1* = neutrophil cytosol factor 1; (G) *NDUFB2* = NADH ubiquinone oxidoreductase subunit B2; (H) *NDUFB7* = NADH ubiquinone oxidoreductase subunit B7; (I) *TXNRD2* = thioredoxin reductase 2.

The *BCL2A1* gene was found to be highly expressed in NBW piglets (Fig 2A). The expression of the genes encoding the pro-inflammatory chemokine CCL19 and the interleukin 8 was significantly modulated between piglets from different ages with the highest levels being measured after weaning as observed with the PCR arrays (Fig 2B and 2D). The mRNA expression of *GPx2* and metallothionein 3 (*MT3*) is also significantly regulated throughout the peri-weaning period (Fig 2C and 2E). The results of the PCR arrays revealing that genes encoding subunits of the mitochondrial electron transport chain are down-regulated after weaning were confirmed by quantitative PCR for *NDUFB2* which also revealed that its expression is influenced by birth weight (Fig 2G). The *NDUFB7* gene is also differentially expressed throughout the peri-weaning period with the lowest levels being observed at d 23 in comparison to d 35 (Fig 2H). As for the liver, the expression of the mitochondrial thioredoxin reductase (*TXNRD2*) in the intestinal mucosa of NBW piglets was found to be significantly higher than in their LBW counterparts as well as to be modulated throughout the peri-weaning period (Fig 2I).

### Expression of targeted genes in kidney of both LBW and NBW piglets throughout the peri-weaning period

**Fig 3:**
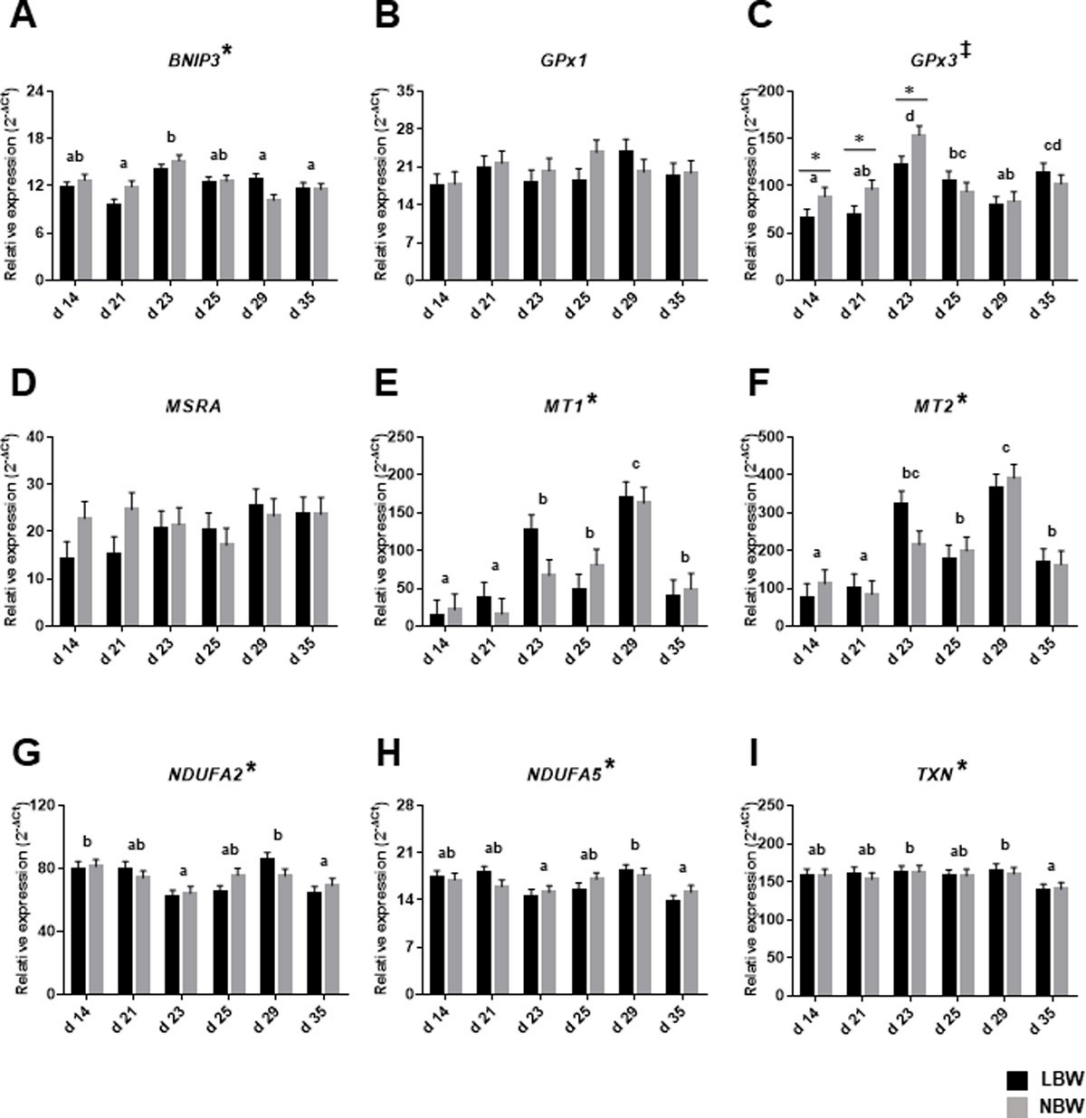
Relative mRNA levels of *BNIP3*, *GPx1*, *GPx3*, *MRSA*, *MT1*, *MT2*, *NDUFA2*, *NDUFA5* and *TXN* were analyzed in kidney of LBW and NBW piglets of different ages throughout the peri-weaning period. Bars in the graphs represent least-square means ± SEM of 10 piglets. Asterisk (*) indicates a significant effect of the age on means at P ≤ 0.05. Dagger (†) indicates a significant effect of the birth weight on means at P ≤ 0.05. Pound (#) indicates that means tended to be affected by birth weight at P < 0.10. Double cross(‡) indicates a significant interaction between age and birth weight at P < 0.01. Detailed *P-Values* for main effects and interactions are available in S15 Table in S1 File comparative analysis with litter and gender as random cofactors in S16 Table in S1 File and box plot of raw data distribution in S4 Fig in S1 File. Ages with different letters (a, b, c, d) within the graphs are statistically different at P ≤ 0.05.LBW = low-birth weight piglets; NBW = normal-birth weight piglets; (A) *BNIP3* = BCL2 interacting protein 3; (B) *GPx1* = glutathione peroxidase 1; (C) *GPx3* = glutathione peroxidase 3; (D) *MRSA* = methionine sulfoxide reductase A; (E) *MT1* = metallothionein 1A; (F) *MT2* = metallothionein 2B; (G) *NDUFA2* = NADH ubiquinone oxidoreductase subunit A2; (H) *NDUFA5* = NADH ubiquinone oxidoreductase subunit A5; (I) TXN = thioredoxin.

For the kidney, targeted genes were selected based on the array results obtained for the liver samples as well as from previous study [19]. The quantitative PCR results indicated that the expression of the *BNIP3* genes is regulated by the ages of the piglets but, in contrast to what was observed in liver, is not influenced by the birth weight (Fig 3A). The expression of the intracellular *GPx1* was not found to be modulated by birth weight and the age of piglets but the extracellular *GPx3* is influenced by both factors (interaction birth weight x age) with the highest expression levels being measured at d 23 for both groups (Fig 3C). The mRNA expression levels of GPx3 at d14, d21 and d23 where is higher in NBW piglets. In opposite to the liver, no difference of mRNA expression was detected for the *MSRA* gene. The metallothioneins 1 and 2 are highly expressed during the post-weaning period as it is also the case for *MT3* in the intestinal mucosa (Fig 3D and 3F). Finally, as it was observed for the liver and the intestine, the expression of genes encoding subunits of the mitochondrial respiratory chain (*NDUFA2* and *NDUFA5*) is significantly affected by the age of the piglets from both groups (Fig 3G and 3H). The gene encoding thioredoxin (*TXN*) is modulated throughout the experimental period with low levels observed at d 35 when compared to d 29 (Fig 3I).

### Expression of inflammatory cytokines in plasma of both LBW and NBW piglets throughout the peri-weaning period

**Fig 4:**
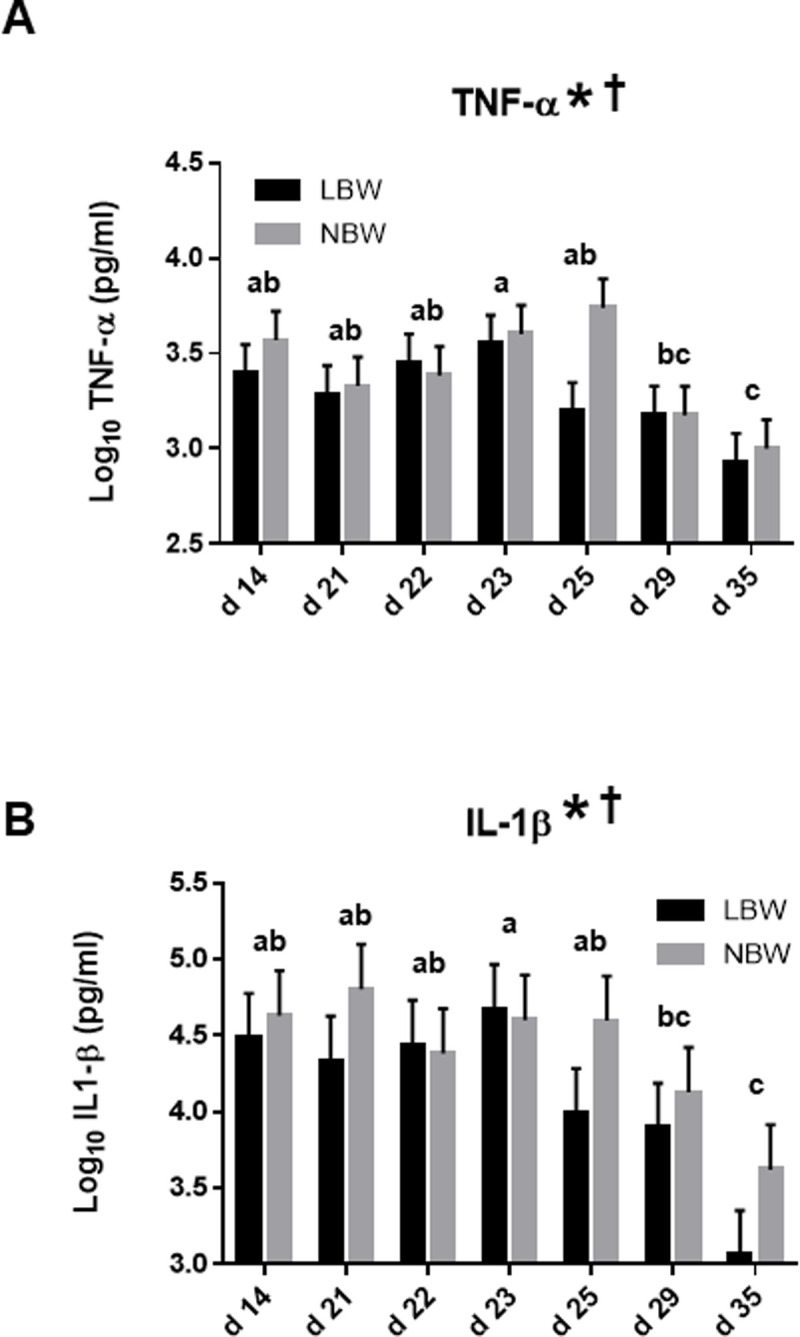
Levels of TNF-α and IL-1β in plasma samples of LBW and NBW piglets of different ages throughout the peri-weaning period. Bars in the graphs represent Log_10_ means ± SEM of 10 piglets of TNF-α (A) and IL-1β (B) in plasma samples. Asterisk (*) indicates a significant effect of the age on means at P ≤ 0.05. Dagger (†) indicates a significant effect of the birth weight on means at P ≤ 0.05. Ages with different letters (a, b, c) within the graphs are statistically different at P ≤ 0.05. Detailed P-Values for main effects and interactions are available in S15 Table in S1 File, comparative analysis with litter and gender as random cofactors in S16 Table in S1 File and box plot of data distribution in S5 Fig in S1 File. TNF-α = Tumor necrosis factor-α; IL-1β = Interleukin-1β.

The inflammatory status of LBW and NBW piglets during the peri-weaning period was assessed by performing ELISA analyses to measure the levels of tumor necrosis factor (TNF-α) and interleukin 1 (IL-1β) in plasma samples (Fig 4). Results from these experiments indicated that the age of the piglets had a significant impact on the plasma levels of both inflammatory cytokines. The lowest levels of TNF-α and IL-1β are detected at the end of the experimental period at d 29 and 35. We further observed that these inflammatory cytokines were highly expressed in NBW piglets when compared with their LBW counterparts.

## Discussion

We recently reported that oxidative stress conditions are rapidly induced after weaning in piglets of 21 days of age and lasted for at least two weeks [19]. The LBW piglets are affected by more severe mitochondrial energetic deficiencies following weaning and sustain higher levels of oxidative damage than NBW piglets throughout the peri-weaning period. Here, we provide some insights about the molecular mechanisms underlying these specific results related to mitochondrial function and oxidative stress. This is the first report of a study utilizing both pathway-focused PCR-based profiler arrays and quantitative RT-PCR analyses to investigate gene expression in normal and low-birth weight piglets throughout the peri-weaning period. The profiler arrays were performed on samples from liver and intestinal mucosa of NBW piglets. We have previously found that the levels of cellular ATP significantly decreased after weaning in liver samples revealing mitochondrial dysfunction. This specific decrease could be related to a reduced mRNA expression of major components of the mitochondrial respiratory complexes in liver. Our results indicate that the expression of specific subunits of the respiratory complexes of the mitochondrial electron transport chain (ETC) tended to decrease two days after weaning in comparison with the lactation period in liver samples. This tendency has been supported by quantitative RT-PCR for genes encoding the subunits NDUFA2 and NDUFA5 of complex I, a major entry point of respiratory chain which is crucial to establish the driving force for the ATP synthesis by ATP synthase (complex V) [23]. A similar pattern of expression was further observed in kidney for these two subunits of the complex I. In the intestinal mucosa, there are considerably less genes related to mitochondrial ETC which were found to be significantly modulated throughout the peri-weaning period. However, *NDUFB2* was found to be downregulated at d 23 in comparison with d 21 and also tended to be less expressed in LBW piglets. This is in line with previous study indicating that the enzymatic activity of the mitochondrial respiratory complexes is significantly reduced in intestinal mucosa of post-weaned piglets [9]. This sudden decrease in *NDUFB2* mRNA expression may partly explain the post-weaning drop in liver ATP levels since differential expression of mitochondrial ETC related genes is well recognized to have a significant impact on mitochondrial energy metabolism [24]. Such decrease in expression of mitochondrial ETC genes combined to a reduction in ATP production is also frequently associated to superoxide production resulting from defective electron transfer and excessive leaking to oxygen especially at the level of complexes I and III [25]. This is also in accordance with our previous results showing an increase in antioxidant response in tissue samples and accumulation of systemic oxidative damage to macromolecules following weaning [19]. However, this modulation in mRNA expression of mitochondrial ETC related genes does not really explain the higher susceptibility of LBW piglets to oxidative damage since no significant effect of birth weight have been observed for these genes throughout the peri-weaning period.

There was surprisingly not a lot of genes related to the oxidative stress related profiler that were found to be significantly modulated between d 14 and the post-weaning period in liver samples. Among them, very few could be specifically related to mitochondrial oxidative stress. The mRNA expression of arachidonate 12-lipoxygenase (*ALOX12*) tended to be reduced at d 35 in comparison with the lactation period. This 12-Lipoxygenase has been considered to be a relevant source of lipid peroxidation as it deoxygenates membrane-bound arachidonic acid to its corresponding hydroperoxy derivatives in different cellular compartments including mitochondria [26]. *ALOX12* has been recently associated to the induction of an iron-dependent form of regulated cell death, ferroptosis, which occurs in liver through the toxic accumulation of lipid hydroperoxides and promotion of oxidative stress conditions when glutathione-dependant antioxidant defense systems are compromised [27, 28]. Accordingly, the inhibition of *ALOX12* was shown to prevent cell death in hepatic tissue by sustaining glutathione hemostasis through the involvement of GPx antioxidant enzymes and attenuation of oxidative stress [29]. It is therefore possible that the tendency toward a downregulation in mRNA expression of *ALOX12* observed in the present study after weaning could be linked to molecular response aiming to protect liver cells from excessive lipid peroxidation and induction of cell death. Our previous results indicating that the lowest level GPx activity in liver is also observed at d 23 support this hypothesis [19].

Among the genes that were found to be downregulated during the post-weaning period without reaching the significance level, one, *BNIP3*, brought our attention because of its crucial role associated to mitochondrial dysfunction [30]. This protein is localized at the outer mitochondrial membrane and recent studies have demonstrated that it regulates mitochondrial dysfunction and mitophagy homeostasis via numerous cellular signaling pathways [31]. Mitochondria produce energy in the form of ATP from multiple energy sources and this energy is delivered to all cellular compartments to feed various cellular activities. Mitochondrial energy metabolism is tightly controlled to constantly meet the energetic requirements of cells and to utilize the available energy substrates [32]. The control of mitochondrial mass is a crucial determinant of energy metabolism and it is determined by the balance of elongation and degradation. While mitochondrial biogenesis increases mitochondrial mass, this is countered by the role of mitophagy in targeting dysfunctional mitochondria for degradation, resulting in reduced mitochondrial mass [33]. *BNIP3* is recognized as a transcription target in response to mitochondrial dysfunction and causes mitophagy through triggering mitochondrial depolarization and the subsequent sequestrating of the mitochondria into autophagosomes. Induction of mitophagy by *BNIP3* has been shown to be crucial for normal liver energy metabolism by showing that reduced *BNIP3* expression leads to increased mitochondrial mass, mitochondrial dysfunction and excessive ROS production [34]. These defects were exacerbated in period of nutritional deficiencies [31]. Here we demonstrated that *BNIP3* gene is significantly less expressed in the liver of LBW piglets when compared to their NBW counterparts. This correlates with previous results showing lower ATP production and higher oxidative damage in the very same LBW piglets throughout the peri-weaning period [19]. Interestingly, it is known that mitophagy is normally stimulated in response to starvation and energy stress while other organelles remain unaffected [33]. Studies in multiple cells have further shown that *BNIP3*-induced mitophagy acts in a protective role against mitochondrial dysfunction by controlling the ROS generation [35, 36]. It can thus be suggested that reduction of *BNIP3* expression in LBW piglets is associated with defects in the elimination of dysfunctional mitochondria leading to perturbation in mitochondrial homeostasis, energetic deficiency and oxidative stress.

The mRNA expression of glutathione peroxidase 3 (*GPx3*) was found to be downregulated at d 35 in comparison with d 14 in liver of NBW piglets by using profiler arrays but without reaching the significance level following FDR adjustment. Quantitative RT-PCR revealed that the expression of this enzyme is significantly lower in LBW than NBW piglets suggesting a defective antioxidant response. This was further supported by results showing that heme-oxygenase 1 (*HMOX1*) tended to be differentially expressed between LBW and NBW piglets. HMOX1 is an inducible form of heme oxygenase which is upregulated by heavy metals and many stimuli known to promote oxidative stress such as ROS [37]. The induction of *HMOX1* is usually associated with a protective antioxidant response due to its ability of removing free cytotoxic heme and producing carbon monoxide CO and biliverdin which are linked to response against oxidative stress and inflammation [38]. Thioredoxin reductase-2 (TXNRD2) is a mitochondrial enzyme responsible for reducing oxidized thioredoxin-2 (TRX2) and is essential to control mitochondrial oxidative stress status and we found that it was differently expressed between LBW and NBW piglets [39]. Inhibition of *TXNRD2* has been associated with increased production of superoxide and concomitant emission of hydrogen peroxide from mitochondria toward the cytosol. It is well known that when the rate of superoxide production exceeds the scavenging capacity of the antioxidant systems, H_2_O_2_ emission from the mitochondria will increase and promote oxidative damage. This scenario may likely apply to dysfunctional liver mitochondria of LBW characterized by low ATP production, high levels of oxidative damage and decreased mRNA expression of crucial antioxidant enzymes. Interestingly, it was demonstrated that the TXNRD2/TRX2 system is essential to supply reduced NADPH to peroxiredoxin 3 (PRDx3) which is a mitochondrial enzyme responsible of the reduction of H_2_O_2_ to water. These enzymes function in parallel with the mitochondrial GSH/GPx system to assure protection against oxidative stress [40]. The expression of almost all these enzymatic mitochondrial antioxidants were shown to be significantly modulated throughout the peri-weaning with the lowest levels being observed shortly after weaning in liver. This confirms that newly weaned piglets, especially the LBW ones, are highly susceptible to mitochondrial oxidative stress. Another indication to support this conclusion is the significant modulation of methionine sulfoxide reductase A (*MSRA*) with the lowest mRNA expression measured immediately following weaning. The methionine sulfoxide reductases are crucial antioxidant enzymes that reduce oxidized methionine (methionine sulfoxide, MetO) of both proteins and free amino acids to methionine [41]. The MSRA form is mainly expressed in the mitochondria of liver and kidney and it was shown that lack of MSRA dramatically increases sensitivity to oxidative stress under stressful conditions [42]. Interestingly, this decrease in *MSRA* expression has been linked to elevated protein carbonyls accumulations as we have previously observed in plasma samples of weaned piglets [19]. Moreover, we have shown that LBW piglets had higher levels of liver protein carbonyls in liver prior to weaning and this parallel the lower expression of *MSRA* observed in the present study in liver samples.

Weaning is known to induce substantial changes in the morphology of the intestine which are frequently accompanied by intestinal disturbances, inflammation and bacterial infections that could be either transient or long lasting [43, 44]. Here we have studied mRNA expression of mucosal jejunal genes implicated in oxidative stress response as well as others related to energy metabolism, inflammation and apoptosis processes to better defined the molecular mechanisms underlying intestinal weaning stress. We found only few genes encoding subunits of mitochondrial ETC that were modulated after weaning in comparison to d 14 in profiler arrays. This was expected considering that this tissue is less metabolically active than liver but additional RT-PCR analyses still revealed that expression of one subunit of complex I (*NDUFB2* and *NDUFB7*) was significantly modulated throughout the peri-weaning period with lowest levels measured few days after weaning for *NDUFB2*. This suggests that intestinal cells sustained similar mitochondrial energy dysfunction as it was observed in liver. Reduced mitochondrial function is often linked to increase production of ROS and we have recently demonstrated that the intestinal enzymatic activity of antioxidants GPx and SOD were modulated throughout the peri-weaning period in response to oxidative stress conditions. By performing the profiler arrays and quantitative RT-PCR analyses, we have gained more insights about the molecular origins and consequences of this post-weaning oxidative stress within the intestine. Interestingly, we found that the mRNA expression pattern of *GPx2*, the main intestinal form of GPx [45], is in accordance with our previous results on intestinal GPx enzymatic activity in intestinal mucosa with the highest levels observed at d 35. Analysis of mitochondrial *TXNRD2* expression revealed a similar pattern of expression as the one observed in the liver with an upregulation after weaning with a significant difference between both groups of piglets. This indicates that mitochondrial reduction of oxidized thioredoxin-2 (*TRX2*), a crucial step in the control of mitochondrial oxidative stress, is also compromised in the intestinal mucosa of LBW piglets.

The activation of some inflammatory cytokines in the intestine has already been associated with the weaning period [46] and this has been confirmed with more details here by showing that many cytokines present in the corresponding profiler array were upregulated at d 23. Results from quantitative RT-PCR analyses on two selected inflammatory cytokines known to be up-regulated in response to bacterial infection in porcine intestinal cells, *CCL19* and *IL8* [47], further suggest that inflammatory conditions rapidly occurred two days post-weaning and could last for several days. Surprisingly, very few genes related to the apoptosis process were found to be modulated throughout the peri-weaning period with only the gene *BCL2A1* being upregulated at d 35 in comparison to d 14. The expression of this gene was also significantly different between NBW and LBW piglets. This gene is a member of the B-cell lymphoma 2 (BCL2) proteins which are important regulators of cell death [48]. In a physiological context, *BCL2A1* is a highly regulated gene expressed in a variety of tissues that exerts important pro-survival functions on pro-inflammatory cells during an immune response [49]. This suggests that activation of *BCL2A1* following weaning possibly allows these crucial immune cells to survive in an inflammatory context and to protect intestinal cells of piglets in this stressful environment. Lower expression of this gene in LBW may indicate that this process was compromised and may contributed to their increased vulnerability to intestinal disturbances and inflammatory reactions.

The inflammatory status of piglets throughout the peri-weaning period was further analyzed by measuring the circulating levels of pro-inflammatory cytokines IL-1β and TNF-α. We showed that not only the secretion of these inflammatory molecules were modulated during this period but also that NBW piglets have significantly higher levels of both cytokines. This correlates with the observed trend to decrease *IL-8* mRNA expression in LBW piglets. This is also in line with recent results showing that LBW piglets have decreased expression of some inflammatory cytokines in intestinal tissue in comparison to NBW piglets during the lactation period [5]. The results presented here suggest that LBW piglets are still affected by this impaired innate immune response in the post-weaning period. Interestingly, as an inflammatory response regulator of mitophagy, TNF-α has been identified to be an activator of BNIP3 which is required for clearance of dysfunctional mitochondria and also highly expressed in NBW piglets [34]. Additional data further indicate that TNF-α upregulates expression of specific anti-apoptotic proteins and concomitantly decreases expression of specific proteins involved in death signaling is actively implicated in neutrophils under inflammatory conditions. It is thus plausible that TNF-α triggers the expression of the survival gene *BCL2A1* in intestine of post-weaned piglets but significantly less in the LBW ones.

In the current study, the post-weaning increase observed in the expression of metallothioneins (MTs), small cysteine-rich proteins implicated in metal detoxification as well as in zinc and copper homeostasis might be attributable to the zinc oxide (ZnO) levels present in the post-weaned diet [50]. MTs expression is regulated by the amount of cytosolic free zinc ions. Expression of MTs sharply increased at d 29 in intestine, liver and kidney tissues, reflecting increased tissue zinc-binding capacity. Moreover, the high thiol content of metallothioneins allows them to protect against oxidative stress by interacting with and inhibiting ROS. It has been shown that transcription of MT genes is specifically upregulated by ROS through the antioxidant response elements (AREs) located in their promoter regions [51]. This suggests that upregulation of MT expression in the three studied tissues of post-weaned piglets may origin from a response to in-feed ZnO in combination with oxidative stress conditions.

The expression of energy metabolism and oxidative stress related genes in kidney of peri-weaned piglets has never been studied. The results obtained for mRNA expression of genes encoding subunits of the mitochondrial respiratory chain revealed significant impact of the weaning period with lowest levels measured around d 23 as observed in liver and intestinal mucosa. Results for the expression of *GPx3*, the major GPx enzyme known to be expressed in kidney [52], is in line with our previous results on GPx activity in this organ which was significantly higher in the post-weaning period in response to oxidative stress conditions. Moreover, the analysis of mRNA expression of *GPx3* revealed a significant interaction between birthweight and age with suggesting a more efficient transcriptional activation of *GPx3* by oxidative stress in NBW piglets. In contrast to our observation in the liver, the expression of *BNIP3* and *MSRA* are not modulated during the peri-weaning period suggesting that the regulation of mitophagy and reduction of oxidized methionine are respectively different between organs.

Taken together, the results of this study shed a novel light on the molecular mechanisms underlying previous results about post-weaning mitochondrial energetic dysfunction and oxidative stress response in NBW and LBW piglets. We have demonstrated that the post-weaning period is characterized by a significant decrease in genes encoding subunits of mitochondrial respiratory chain linked to an increase in genes implicated in mitochondrial and cellular response against oxidative stress. This specific response is further related to modulation of specific genes implicated in clearance of dysfunctional mitochondria, in establishment of inflammation and in survival of crucial immune cells under these inflammatory conditions. We also established for the first time that the increased oxidative damage and energetic deficit previously shown to affect LBW piglets is linked to reduced mRNA expression of genes encoding major regulators of mitochondrial oxidative stress response, cell survival and mitophagy. Accumulation of dysfunctional mitochondria linked to reduced mitochondrial antioxidant capacity and suboptimal inflammatory response are likely at the origin of the increased vulnerability of LBW piglets to the multiple stressors occurring during the peri-weaning period. This reveals that lower birth weight could have long-lasting detrimental effects on mitochondrial function and protection against oxidative stress. This study has identified clear molecular pathways implicated in weaning stress and thus provides useful genetic markers and targets to test the efficiency of strategy aiming at increasing the robustness of piglets during this problematic period.

## Supporting information

S1 File (DOCX)
