# Supplementary material for: Weaning differentially affects mitochondrial function, oxidative stress, inflammation and apoptosis in normal and low birth weight piglets
Source: PLoS One. 2021 Feb 19;16(2):e0247188. doi: 10.1371/journal.pone.0247188 (PMC7894895; doi:10.1371/journal.pone.0247188)

**S1 Fig.** **Evolution of the bodyweights of low birth weight piglets (LBW) and normal birth weight piglets (NBW) throughout the lactation and the post-weaning period.** Each points in the graph represents means ± standard deviation (SD). The number of LBW and NBW piglets is 60 for d 1, 7 and 14 and then it decreased by 10 piglets per day from d 21 to d 35 (10 piglets per group were slaughtered at each time point starting from d 14).

**S1 Table. Ingredients and nutrients provided as fed basis in weaning diets for piglets**

|  | Weaning Diets | | | | |
| --- | --- | --- | --- | --- | --- |
|  |  |  |  |  |  |
| Ingredients/nutrients | d 21 to 23 | | | d 24 to 29 | d 30 to 35 |
| Digestible energy (MKcal/kg) | 3,420 | | | 3,470 | 3,350 |
| Moisture (%) | 9.19 | | | 9.2 | 10.87 |
| Total protein (%) | 18.45 | | | 18.59 | 20.82 |
| Crude fibre (%) | 2.35 | | | 3.01 | 3.02 |
| Ash (%) | 5.93 | | | 5.57 | 5.73 |
| Fat (%) | 5.21 | | | 6.35 | 4.78 |
| Calcium (%) | 0.65 | | | 0.69 | 0.70 |
| Phosphorus (%) | 0.67 | | | 0.75 | 0.68 |
| Sodium (%) | 0.52 | | | 0.51 | 0.38 |
| Potassium (%) | 0.75 | | | 0.66 | 0.96 |
| Magnesium (%) | 0.14 | | | 0.15 | 0.20 |
| Copper (ppm) | 114.37 | | | 132.28 | 121.09 |
| Zinc (ppm) | 2 850.04 | | | 2 476.49 | 428.38 |
| Selenium (ppm) | 0.80 | | | 0.70 | 0.60 |
| Ca:P | 0.98 | | | 0.92 | 1.03 |

**S2 Table. Primers sequence used for quantitative reverse transcription (RT PCR)**

| Gene | Gene Bank | Name | Sequence 5' to 3' | Product(bp) | |
| --- | --- | --- | --- | --- | --- |
| BCL2A1 | NM_001164511.2 | B-cell lymphoma 2-related protein A1 | F:GGATAAGGCAAAACGGAGGCT | 84 |  |
|  |  |  | R:TAACTTCCACAAAGGTCAGCCA |  |  |
| BNIP3 | XM_003359404.4 | BCL2 interacting protein 3 | F:GGAACACGAGCGTGATGAAGA | 122 |  |
|  |  |  | R:GACGCCTCCCGATGTAGATC |  |  |
| CCL19 | NM_001170516.1 | C-C motif chemokine ligand 19 | F:AGTCACACTACTGGCCCTCA | 89 |  |
|  |  |  | R:CAGACAGGCAGCAGTCTTCA |  |  |
| GPx1 | NM_214201.1 | glutathione peroxidase 1 | F:CAGGTACAGCCGTCGCTTTC | 137 |  |
|  |  |  | R:AAAATCCCGAGAGTAGCACTGTAAC |  |  |
| GPx2 | NM_001115136.1 | glutathione peroxidase 2 | F: TGCAACCAATTTGGACATCAG | 122 |  |
|  |  |  | R: TTCACGTCACACTTCTGGATAAGG |  |  |
| GPx3 | NM_001115155.1 | glutathione peroxidase 3 | F:AAACAGGAACCGGGAGACAA | 156 |  |
|  |  |  | R:AGGACAGGCGTTCTTCAGGAA |  |  |
| HMOX1 | NM_001004027.1 | heme oxygenase 1 | F:GGTCACCCGAGAAGGCTTTA | 97 |  |
|  |  |  | R:GACCGGGTTCTCCTTGTTGT |  |  |
| IL-8 | NM_213867.1 | interleukin 8 | F:AGAACTGAGAAGCAACAACAACAG | 131 |  |
|  |  |  | R:CACAGGAATGAGGCATAGATGTAG |  |  |
| MSRA | XM_021072635.1 | methionine sulfoxide reductase A | F:GCATCACGTCAATGGCAACA | 94 |  |
|  |  |  | R:TCGGACTACTTCTGCATGGC |  |  |
| MT1A | NM_001001266.2 | metallothionein 1A | F:GCTTGGTCTCACCTGCCTC | 119 |  |
|  |  |  | R:CTCTTCTTGCAGGAGGTGCAT |  |  |
| MT2B | XM_003355808.4 | metallothionein 2B | F:GCCTGAAGTTGGGGAGACC | 89 |  |
|  |  |  | R:TAGCAAACGGGTCAGGTTGTAT |  |  |
| MT3 | NM_214056.1 | metallothionein 3 | F:CAAGTGCGAGGGATGCAAAT | 95 |  |
|  |  |  | R:TTACACACGCAATCCTTGGC |  |  |
| NCF1 | NM_001113220.1 | neutrophil cytosol factor 1 | F:GCGCTTCACTGAGATCTACGA | 127 |  |
|  |  |  | R:CTGCCCATCAAACCAACGTG |  |  |
| NDUFA2 | XM_003124046.4 | NADH ubiquinone oxidoreductase subunit A2 | F:TCTGATGTGCAGCCCAAGC | 120 |  |
|  |  |  | R:TTTGCCACTTAGCACATTCTCC |  |  |
| NDUFA5 | XM_003134727.3 | NADH ubiquinone oxidoreductase subunit A5 | F:GGTTAAAGCGGAACCAGATGTTA | 132 |  |
|  |  |  | R: GGCTCCCATGGCTTCCA |  |  |
| NDUFB2 | NM_001244885.1 | NADH ubiquinone oxidoreductase subunit B2 | F:CGCTTTTGGCACGACTCAGA | 81 |  |
|  |  |  | R:GCCTAATTCTTCATCCGTCCAC |  |  |
| NDUFB7 | XM_003480775.4 | NADH ubiquinone oxidoreductase subunit B7 | F:CCACCTTCCCGCCTGACTA | 88 |  |
|  |  |  | R:ATCAGCTGTGCGTCGTTCA |  |  |
| PRDx3 | NM_001244531.1 | peroxideroxin 3 | F:TCTGGCTTGGATAAATACACC | 115 |  |
|  |  |  | R:GACCTTCTAACAGCACACC |  |  |
| TXN | XM_021083263.1 | thioredoxin | F: ATTCCAATGTCGTGTTCCTTGAA | 127 |  |
|  |  |  | R:TCCTTGTTAGCTCCAGAAAATTCAC |  |  |
| TXNRD2 | NM_001168702.1 | thioredoxin reductase 2 | F:CGAAAGGCGGAAAAGAGATTC | 104 |  |
|  |  |  | R:TTCCATATTCCACAGCACCTTC |  |  |
| B2M | NM_213978.1 | beta-2-Microglobulin | F:CTTTCAGCAAGGACTGGTCTTTCTAC | 134 |  |
|  |  |  | R:GTGGTCTCGATCCCACTTAACTATC |  |  |
| GAPDH | NM_001206359.1 | glyceraldehyde-3-phosphate dehydrogenase | F:CCCCAACGTGTCGGTTGT | 91 |  |
|  |  |  | R:CTCGGACGCCTGCTTCAC |  |  |
| RPL13A | NM_001244068.1 | ribosomal protein L13A | F:GTGGCCAAGCAGGTACTTCTG | 136 |  |
|  |  |  | R:GGGACGGGTTGGTATTCATG |  |  |
| RPL4 | XM_005659862.3 | ribosomal protein L4 | F:AGGAGGCTGTTCTGCTTCTG | 185 |  |
|  |  |  | R:TCCAGGGATGTTTCTGAAGG |  |  |

**S3 Table. Relative expression levels of the genes of the oxidative stress in liver in post-weaning (D23 and D35) in relation to the pre-weaning period (D-14)**

| Symbol | Description | (d23/14) | Fold d23 | FDR d23 | (d35/14) | Fold d35 | FDR d35 |
| --- | --- | --- | --- | --- | --- | --- | --- |
| ALB | Albumin | -2.60 | 0.040 | 0.553 | -3.77 | <0.01 | 0.037 |
| ALOX12 | Arachidonate 12-lipoxygenase | -5.82 | 0.013 | 0.553 | -5.01 | <0.01 | 0.083 |
| AOX1 | Aldehyde oxidase-like | 1.05 | 0.774 | 0.833 | 1.70 | 0.167 | 0.270 |
| APOE | Apolipoprotein E | -1.68 | 0.036 | 0.553 | -2.26 | 0.01 | 0.099 |
| BAG2 | BAG family molecular chaperone regulator 2-like | -1.31 | 0.271 | 0.553 | -1.97^A^ | 0.131 | 0.230 |
| BNIP3 | BCL2/adenovirus E1B 19 kDa protein-interacting protein 3-like | -1.53 | 0.283 | 0.553 | -1.88^A^ | 0.108 | 0.230 |
| CAT | Catalase | -1.61 | 0.090 | 0.553 | -1.29 | 0.123 | 0.230 |
| CCL5 | Chemokine (C-C motif) ligand 5 | -2.01 | 0.201 | 0.553 | -2.24 | <0.01 | 0.083 |
| CYGB | Cytoglobin-like | -4.23 | 0.115 | 0.553 | -3.68^A^ | 0.064 | 0.191 |
| DHCR24 | 24-dehydrocholesterol reductase | -2.10 | 0.260 | 0.553 | -1.93 | 0.038 | 0.183 |
| DUOX1 | Dual oxidase 1 | -2.02^B^ | 0.340 | 0.563 | -4.06^B^ | 0.203 | 0.305 |
| DUOX2 | Dual oxidase 2 | -7.34^B^ | 0.129 | 0.553 | -12.78^B^ | 0.056 | 0.188 |
| DUSP1 | Dual specificity phosphatase 1 | -1.80 | 0.121 | 0.553 | -1.94 | 0.038 | 0.183 |
| EPHX2 | Epoxide hydrolase 2, cytoplasmic | -1.32 | 0.244 | 00.553 | 1.06 | 0.591 | 0.628 |
| EPX | Eosinophil peroxidase | 1.03^B^ | 0.547 | 0.682 | -1.48^B^ | 0.366 | 0.440 |
| FHL2 | Four and a half LIM domains protein 2-like | -2.37^B^ | 0.265 | 0.553 | -9.65^B^ | 0.098 | 0.230 |
| FOXM1 | Forkhead box M1 | -8.15^B^ | 0.073 | 0.553 | -3.62 | 0.049 | 0.188 |
| FTH1 | Ferritin, heavy polypeptide 1 | -1.35 | 0.367 | 0.563 | -1.37 | 0.290 | 0.392 |
| GCLC | Glutamate-cysteine ligase, catalytic subunit | -1.68 | 0.366 | 0.563 | -1.73 | 0.262 | 0.386 |
| GCLM | Glutamate-cysteine ligase, modifier subunit | -1.78 | 0.389 | 0.563 | 1.62 | 0.359 | 0.440 |
| GLA | Alpha-galactosidase A | -3.46 | 0.050 | 0.553 | -2.20 | 0.055 | 0.188 |
| GP91-PHOX | NADPH oxidase heavy chain subunit | -3.82 | 0.092 | 0.553 | -6.86 | 0.025 | 0.163 |
| GPX1 | Glutathione peroxidase 1 | -1.76 | 0.381 | 0.563 | 1.22 | 0.381 | 0.451 |
| GPX2 | Glutathione peroxidase 2 (gastrointestinal) | -2.92^B^ | 0.281 | 0.553 | -14.55^B^ | 0.116 | 0.230 |
| GPX3 | Glutathione peroxidase 3 (plasma) | -3.04 | 0.068 | 0.553 | -4.57 | 0.015 | 0.126 |
| GPX4 | Glutathione peroxidase 4 (phospholipid hydroperoxidase) | -1.27 | 0.141 | 0.553 | -1.30 | 0.062 | 0.191 |
| GPX5 | Glutathione peroxidase 5 (epididymal androgen-related protein) | -3.40^B^ | 0.326 | 0.563 | -2.20^B^ | 0.277 | 0.388 |
| GSS | Glutathione synthetase | -2.98 | 0.134 | 0.553 | -3.88 | 0.060 | 0.191 |
| GSTZ1 | Glutathione S-transferase zeta 1 | -3.07 | 0.260 | 0.553 | -3.00 | 0.178 | 0.271 |
| HMOX1 | Heme oxygenase (decycling) 1 | -2.35 | 0.206 | 0.553 | -3.40 | 0.091 | 0.230 |
| HSP70.2 | Heat shock protein 70.2 | -1.80 | 0.378 | 0.563 | -4.02 | 0.130 | 0.230 |
| HSP90AA1 | 90-kDa heat shock protein | -1.27 | 0.760 | 0.829 | -1.59 | 0.173 | 0.270 |
| KRT1 | Keratin 1 | 1.64^B^ | 0.425 | 0.595 | -1.13 | 0.327 | 0.423 |
| LHPP | Phospholysine phosphohistidine inorganic pyrophosphate phosphatase | -3.30 | 0.154 | 0.553 | -3.86 | 0.078 | 0.218 |
| LOC100049683 | Solute carrier family 7 (anionic amino acid transporter light chain, xc- system), member 11 | -1.63^B^ | 0.500 | 0.656 | -1.01^B^ | 0.609 | 0.640 |
| LOC100516395 | Lactoperoxidase-like | -6.30^B^ | 0.645 | 0.753 | 1.88^B^ | 0.506 | 0.569 |
| TTN | Titin-like | -2.87^B^ | 0.273 | 0.553 | -9.85^B^ | 0.115 | 0.230 |
| LOC100622575 | Pancreatic secretory trypsin inhibitor-like | -5.51^B^ | 0.832 | 0.885 | -42.37^B^ | 0.805 | 0.824 |
| LOC100739163 | Glutathione S-transferase P | 1.12 | 0.485 | 0.647 | 1.68 | 0.354 | 0.440 |
| LOC396625 | Glutathione reductase | -1.40 | 0.211 | 0.553 | -1.45 | 0.084 | 0.222 |
| LOC733635 | Aldo-keto reductase family 1 member C2-like | -2.76 | 0.142 | 0.553 | -5.29 | 0.327 | 0.423 |
| MB | Myoglobin | -1.86^C^ | 0.244 | 0.553 | -1.13 | 0.345 | 0.439 |
| MBL2 | Mannose-binding lectin (protein C) 2, soluble | -2.53 | 0.368 | 0.563 | -1.10 | 0.670 | 0.695 |
| MGST3 | Microsomal glutathione S-transferase 3 | -1.75 | 0.159 | 0.553 | 1.14 | 0.110 | 0.230 |
| MPO | Myeloperoxidase | -5.20^B^ | 0.386 | 0.563 | -2.70^B^ | 0.277 | 0.388 |
| MPV17 | MpV17 mitochondrial inner membrane protein | -2.27^B^ | 0.078 | 0.553 | -3.17^B^ | 0.053 | 0.188 |
| MSRA | Peptide methionine sulfoxide reductase | -5.37 | 0.705 | 0.789 | -2.95 | <0.01 | 0.068 |
| MT-III | Metallothionein-III | -2.12 | 0.089 | 0.553 | -5.96 | 0.024 | 0.163 |
| NCF1 | Neutrophil cytosolic factor 1 | -3.54^A^ | 0.311 | 0.563 | -7.16 | 0.164 | 0.269 |
| NCF2 | Neutrophil cytosolic factor 2 | -2.77^B^ | 0.260 | 0.553 | -5.57^B^ | 0.129 | 0.230 |
| NCOA7 | Nuclear receptor coactivator 7 | -1.95 | 0.376 | 0.563 | -3.09 | 0.275 | 0.388 |
| NOS2 | Nitric oxide synthase 2, inducible | -3.30^B^ | 0.127 | 0.553 | -2.64^B^ | 0.053 | 0.188 |
| NOX4 | NADPH oxidase 4 | -20.07^B^ | 0.552 | 0.682 | -61.92^B^ | 0.283 | 0.390 |
| NOX5 | NADPH oxidase, EF-hand calcium binding domain 5 | 1.68 | 0.668 | 0.759 | -2.82 | 0.067 | 0.193 |
| NQO1 | NAD(P)H dehydrogenase, quinone 1 | -1.02 | 0.190 | 0.553 | 2.75 | 0.305 | 0.407 |
| NUDT1 | Nudix (nucleoside diphosphate linked moiety X)-type motif 1 | -2.79 | 0.250 | 0.553 | -1.30 | 0.150 | 0.257 |
| OXR1 | Oxidation resistance 1 | -1.61 | 0.229 | 0.553 | -1.65 | 0.117 | 0.230 |
| OXSR1 | Oxidative-stress responsive 1 | -2.88 | 0.529 | 0.682 | -3.79 | 0.429 | 0.501 |
| PDLIM1 | PDZ and LIM domain 1 | -1.37 | 0.987 | 0.987 | -1.38 | <0.01 | 0.068 |
| PRDX1 | Peroxiredoxin-1-like | -1.08 | 0.751 | 0.829 | 1.74 | 0.994 | 0.994 |
| PRDX2 | Peroxiredoxin 2 | -1.10 | 0.868 | 0.911 | 1.02 | 0.053 | 0.188 |
| PRDX3 | Peroxiredoxin 3 | 1.10 | 0.477 | 0.647 | 1.75 | 0.590 | 0.628 |
| PRDX4 | Peroxiredoxin 4 | -1.20 | 0.645 | 0.753 | -1.09 | 0.840 | 0.850 |
| PRDX5 | Peroxiredoxin 5 | -1.05 | 0.618 | 0.742 | 1.30 | 0.039 | 0.183 |
| PRDX6 | Peroxiredoxin 6 | 1.18 | 0.046 | 0.553 | 1.70 | <0.01 | 0.083 |
| PRNP | Prion protein | -2.16 | 0.546 | 0.682 | -1.97 | 0.021 | 0.158 |
| PTGR1 | Prostaglandin reductase 1 | 1.04 | 0.217 | 0.553 | 3.61 | 0.111 | 0.230 |
| PTGS1 | Prostaglandin-endoperoxide synthase 1 (prostaglandin G/H synthase and cyclooxygenase) | -3.07 | 0.159 | 0.553 | -3.55 | 0.082 | 0.222 |
| PTGS2 | Prostaglandin G/H synthase-2 | -2.23^B^ | 0.413 | 0.588 | -2.56^B^ | 0.515 | 0.569 |
| RNF7 | Ring finger protein 7 | -1.28 | 0.220 | 0.553 | -1.07 | 0.107 | 0.230 |
| SCARA3 | Scavenger receptor class A, member 3 | -4.99^B^ | 0.570 | 0.694 | -14.1^B^ | 0.119 | 0.230 |
| SEPP1 | Selenoprotein P, plasma, 1 | 1.52 | 0.295 | 0.563 | 2.12 | 0.174 | 0.271 |
| SFTPD | Surfactant protein D | -2.46^B^ | 0.107 | 0.553 | -9.03^B^ | 0.030 | 0.181 |
| SIRT2 | Sirtuin 2 | -2.20 | 0.924 | 0.958 | -4.07 | 0.568 | 0.620 |
| SOD1 | Superoxide dismutase 1, soluble | 1.05 | 0.209 | 0.553 | -1.14 | 0.123 | 0.230 |
| SOD3 | Extracellular superoxide dismutase | -1.60 | 0.063 | 0.553 | -2.23 | 0.043 | 0.188 |
| SQSTM1 | Sequestosome-1-like | -1.29 | 0.483 | 0.647 | -1.54 | 0.513 | 0.569 |
| TRAPPC6B | Trafficking protein particle complex subunit 6B-like | -1.15^A^ | 0.335 | 0.563 | -1.09^B^ | 0.162 | 0.269 |
| TTNLOC100620261 | Titin | -4.28^B^ | 0.976 | 0.987 | -37.57 | 0.038 | 0.183 |
| TXN | Thioredoxin | 1.33 | 0.654 | 0.753 | 2.79 | 0.013 | 0.117 |
| TXNRD1 | Thioredoxin reductase 1 | 1.29 | 0.385 | 0.563 | 1.24 | 0.366 | 0.440 |
| TXNRD2 | Thioredoxin reductase 2 | -1.62 | 0.328 | 0.563 | -2.06 | <0.01 | 0.083 |
| UCP2 | Uncoupling protein 2 (mitochondrial, proton carrier) | -2.08^B^ | 0.259 | 0.553 | -3.81^B^ | 0.102 | 0.230 |
| VIMP | Selenoprotein S | 1.24 | 0.978 | 0.987 | 1.65 | 0.477 | 0.549 |

A: This gene's average threshold cycle is relatively high (> 30) in either the control or the test sample, and is reasonably low in the other sample (< 30). B: This gene's average threshold cycle is relatively high (> 30), meaning that its relative expression level is low, in both control and test samples, and the p-value for the fold-change is either unavailable or relatively high (p > 0.05). C: This gene's average threshold cycle is either not determined or greater than the defined cut-off (default 35), in both samples meaning that its expression was undetected, making this fold-change result erroneous and un-interpretable. The FDR is the False Discovery Rate adjusted p-values.

**S4 Table. Relative expression levels of the genes of the energy metabolism in liver in post-weaning (D23 and D35) in relation to the pre-weaning period (D-14)**

| Symbol | Description | (d23/14) | Fold d23 | FDR d23 | (d35/14) | Fold d35 | FDR d35 |
| --- | --- | --- | --- | --- | --- | --- | --- |
| ARRDC3 | Arrestin domain containing 3 | -1.06 | 0.879 | 0.901 | -1.42 | 0.173 | 0.281 |
| ASB1 | Ankyrin repeat and SOCS box protein 1-like | -3.26^A^ | 0.111 | 0.267 | -4.20^A^ | 0.060 | 0.198 |
| ATP12A | ATPase, H+/K+ transporting, nongastric, alpha polypeptide | -1.32^B^ | 0.278 | 0.376 | -1.11^B^ | 0.533 | 0.649 |
| ATP4A | ATPase, H+/K+ exchanging, alpha polypeptide | -3.97^B^ | 0.101 | 0.267 | -11.6^B^ | 0.110 | 0.243 |
| ATP4B | ATPase, H+/K+ exchanging, beta polypeptide | -14.5^B^ | 0.143 | 0.293 | -2.24^B^ | 0.141 | 0.271 |
| ATP5A1 | ATP synthase, H+ transporting, mitochondrial F1 complex, alpha subunit 1, cardiac muscle | -2.69 | <0.01 | 0.165 | -1.66 | <0.01 | 0.129 |
| ATP5B | ATP synthase, H+ transporting, mitochondrial F1 complex, beta polypeptide | -1.54 | 0.048 | 0.254 | -1.49 | 0.083 | 0.226 |
| ATP5E | ATP synthase, H+ transporting, mitochondrial F1 complex, epsilon subunit | -1.50 | 0.153 | 0.299 | 1.02 | 0.890 | 0.934 |
| ATP5F1 | ATP synthase, H+ transporting, mitochondrial Fo complex, subunit B1 | -1.02 | 0.804 | 0.866 | -1.48 | 0.153 | 0.2718 |
| ATP5G1 | ATP synthase, H+ transporting, mitochondrial Fo complex, subunit C1 (subunit 9) | -2.34 | 0.126 | 0.286 | -1.68 | 0.102 | 0.236 |
| LOC100037988 | ATP synthase, H+ transporting, mitochondrial Fo complex, subunit C2 (subunit 9) | -1.67^B^ | 0.993 | 0.998 | -1.21^B^ | 0.457 | 0.572 |
| ATP5G3 | ATP synthase, H+ transporting, mitochondrial Fo complex, subunit C3 (subunit 9) | 54.32 | 0.879 | 0.901 | 73.04 | 0.382 | 0.493 |
| ATP5H | ATP synthase, H+ transporting, mitochondrial Fo complex, subunit d | -1.05 | 0.301 | 0.389 | -1.19 | 0.177 | 0.281 |
| ATP5I | ATP synthase, H+ transporting, mitochondrial Fo complex, subunit E | -1.23 | 0.391 | 0.469 | -1.16 | 0.651 | 0.729 |
| ATP5L | ATP synthase, H+ transporting, mitochondrial Fo complex, subunit G | -1.10 | 0.731 | 0.808 | 1.02 | 0.939 | 0.949 |
| ATP6V0A2 | ATPase, H+ transporting, lysosomal V0 subunit a2 | -2.37 | 0.197 | 0.318 | -2.27 | 0.151 | 0.271 |
| ATP6V0D2 | ATPase, H+ transporting, lysosomal 38kDa, V0 subunit d2 | -8.64^A^ | 0.087 | 0.267 | -1.42^A^ | 0.171 | 0.281 |
| ATP6V1C2 | ATPase, H+ transporting, lysosomal 42kDa, V1 subunit C2 | -2.79 | 0.058 | 0.254 | -3.49 | 0.031 | 0.156 |
| ATP6V1E2 | ATPase, H+ transporting, lysosomal 31kDa, V1 subunit E2 | -2.12^B^ | 0.189 | 0.316 | -12.07^B^ | 0.096 | 0.235 |
| ATP6V1G3 | V-type proton ATPase subunit G 3-like | -3.51 | <0.01 | 0.086 | -2.92 | <0.01 | 0.087 |
| BCS1L | BCS1-like (S. cerevisiae) | -2.64 | 0.162 | 0.306 | -4.86^A^ | 0.104 | 0.236 |
| Cox5b | Mitochondrial cytochrome c oxidase subunit Vb | -2.11 | 0.062 | 0.254 | -1.34 | 0.152 | 0.271 |
| COX6A1 | Cytochrome c oxidase subunit VIa polypeptide 1 | 1.07 | 0.767 | 0.837 | -1.04 | 0.676 | 0.747 |
| COX6C | COX6C protein | -1.00 | 0.854 | 0.896 | 1.02 | 0.934 | 0.949 |
| COX7A2 | COX7A2 protein | -1.12 | 0.677 | 0.768 | -1.18 | 0.327 | 0.436 |
| DNAJB1 | DnaJ (Hsp40) homolog, subfamily B, member 1 | -1.50 | 0.205 | 0.323 | -2.92 | 0.035 | 0.156 |
| EDN1 | Endothelin 1 | -2.50^B^ | 0.150 | 0.299 | -3.38^B^ | 0.061 | 0.198 |
| GADD45B | Growth arrest and DNA-damage-inducible, beta | -2.21^A^ | 0.207 | 0.323 | -2.73^A^ | 0.146 | 0.271 |
| HSP70.2 | Heat shock protein 70.2 | -2.73 | 0.135 | 0.293 | -6.41 | 0.015 | 0.141 |
| LHPP | Phospholysine phosphohistidine inorganic pyrophosphate phosphatase | -5.13 | 0.106 | 0.267 | -6.45 | 0.098 | 0.235 |
| LOC100154992 | Mitochondrial inner membrane protein OXA1L-like | -1.87 | 0.312 | 0.398 | -2.83 | 0.557 | 0.668 |
| LOC100156375 | COX15 homolog, cytochrome c oxidase assembly protein | -3.69 | 0.125 | 0.286 | -1.81 | 0.036 | 0.156 |
| LOC100156879 | Ubiquinol--cytochrome c reductase | -2.00 | 0.107 | 0.267 | -2.49 | 0.206 | 0.309 |
| LOC100156967 | Cytochrome c oxidase subunit 5A, mitochondrial-like | -2.48 | 0.2764 | 0.376 | -1.25 | 0.235 | 0.346 |
| LOC100157935 | Cytochrome c oxidase subunit 8C, mitochondrial-like | -4.50^B^ | 0.064 | 0.254 | -6.26^B^ | 0.316 | 0.428 |
| LOC100511690 | Cytochrome c1, heme protein, mitochondrial-like | -2.38 | 0.301 | 0.389 | -2.34 | 0.283 | 0.403 |
| LOC100517408 | Cytochrome c oxidase subunit 7B2, mitochondrial-like | 2.11B | 0.220 | 0.335 | -14.40^B^ | 0.205 | 0.309 |
| LOC100519366 | Cytochrome c oxidase subunit 6B1-like | 1.51B | 0.853 | 0.896 | 1.95^B^ | 0.083 | 0.226 |
| LOC100519594 | Cytochrome c oxidase subunit 6A2, mitochondrial-like | -2.56^A^ | 0.329 | 0.407 | -3.26^A^ | 0.137 | 0.271 |
| LOC100522725 | Cytochrome c oxidase subunit 8A, mitochondrial-like | -2.48 | 0.012 | 0.171 | -2.57 | <0.01 | 0.129 |
| LOC100624067 | Cytochrome c oxidase subunit 4 isoform 1, mitochondrial-like | -1.94 | 0.080 | 0.267 | -1.51 | 0.0743 | 0.223 |
| LOC100624950 | Cytochrome b561 domain-containing protein 1-like | -1.31 | 0.031 | 0.235 | -1.89 | <0.01 | 0.129 |
| LOC100739238 | Low-density lipoprotein receptor-related protein 5-like | -3.19 | 0.192 | 0.316 | -7.24 | 0.041 | 0.158 |
| NDUFB6 | NADH dehydrogenase 1 beta subcomplex 6 | -2.55 | 0.175 | 0.306 | -1.34 | 0.116 | 0.249 |
| ATP5O | ATP synthase H+-transporting mitochondrial F1 complex O subunit | 1.07 | <0.01 | 0.074 | 1.03 | 0.013 | 0.135 |
| NDUFA1 | NADH dehydrogenase [ubiquinone] 1 alpha subcomplex subunit 1-like | -2.30 | 0.240 | 0.342 | -1.57 | 0.037 | 0.156 |
| NDUFA10 | NADH dehydrogenase [ubiquinone] 1 alpha subcomplex subunit 10, mitochondrial-like | -1.27 | 0.227 | 0.335 | -2.17 | 0.183 | 0.285 |
| NDUFA11 | NADH dehydrogenase (ubiquinone) 1 alpha subcomplex, 11, 14.7kDa | -1.65 | 0.364 | 0.443 | -1.84 | 0.733 | 0.799 |
| NDUFA2 | NADH dehydrogenase (ubiquinone) 1 alpha subcomplex, 2, 8kDa | -6.73^A^ | 0.139 | 0.293 | 1.78 | 0.129 | 0.271 |
| NDUFA3 | NADH dehydrogenase [ubiquinone] 1 alpha subcomplex subunit 3-like | -2.16 | 0.169 | 0.306 | -2.28 | 0.155 | 0.271 |
| NDUFA4 | NADH dehydrogenase (ubiquinone) 1 alpha subcomplex, 4, 9kDa | -1.24 | 0.026 | 0.223 | -1.22 | 0.480 | 0.593 |
| NDUFA5 | NADH dehydrogenase [ubiquinone] 1 alpha subcomplex subunit 5-like | -1.59 | <0.01 | 0.086 | -1.04 | <0.01 | 0.121 |
| NDUFA6 | NADH dehydrogenase (ubiquinone) 1 alpha subcomplex, 6, 14kDa | -4.09^A^ | 0.055 | 0.254 | -7.78^A^ | 0.864 | 0.918 |
| NDUFA7 | NADH dehydrogenase [ubiquinone] 1 alpha subcomplex subunit 7-like | -1.51 | 0.096 | 0.267 | -1.01 | 0.176 | 0.281 |
| NDUFA8 | NADH dehydrogenase (ubiquinone) 1 alpha subcomplex, 8, 19kDa | -1.63 | 0.282 | 0.376 | -1.41 | 0.288 | 0.403 |
| NDUFA9 | NADH dehydrogenase [ubiquinone] 1 alpha subcomplex subunit 9, mitochondrial-like | -3.55^B^ | 0.225 | 0.335 | -2.99^B^ | 0.060 | 0.198 |
| NDUFAB1 | NADH dehydrogenase (ubiquinone) 1, alpha/beta subcomplex, 1, 8kDa | -1.42 | 0.317 | 0.398 | -1.56 | 0.139 | 0.271 |
| NDUFB10 | NADH dehydrogenase [ubiquinone] 1 beta subcomplex subunit 10-like | -1.19 | 0.238 | 0.342 | -1.33 | 0.633 | 0.718 |
| NDUFB2 | NADH dehydrogenase (ubiquinone) 1 beta subcomplex, 2, 8kDa | -1.31 | 0.081 | 0.267 | -1.07 | <0.01 | 0.121 |
| NDUFB3 | NADH dehydrogenase (ubiquinone) 1 beta subcomplex, 3, 12kDa | -1.38^B^ | 0.046 | 0.254 | 1.11 | 0.0170 | 0.143 |
| NDUFB4 | NADH dehydrogenase [ubiquinone] 1 beta subcomplex subunit 4-like | -1.54 | 0.043 | 0.254 | -1.59 | 0.564 | 0.668 |
| NDUFB5 | NADH dehydrogenase (ubiquinone) 1 beta subcomplex, 5, 16kDa | -66.13^A^ | 0.020 | 0.216 | -1.11 | 0.162 | 0.278 |
| NDUFB7 | NADH dehydrogenase (ubiquinone) 1 beta subcomplex, 7, 18kDa | -6.55 | 0.021 | 0.216 | -88.59 | 0.012 | 0.135 |
| NDUFB8 | NADH dehydrogenase (ubiquinone) 1 beta subcomplex, 8, 19kDa | -1.40 | 0.258 | 0.361 | 1.13 | 0.933 | 0.949 |
| NDUFB9 | NADH dehydrogenase (ubiquinone) 1 beta subcomplex, 9, 22kDa | -1.11 | 0.481 | 0.561 | -1.00 | 0.818 | 0.881 |
| NDUFC1 | NADH dehydrogenase (ubiquinone) 1, subcomplex unknown, 1, 6kDa | -1.45 | 0.179 | 0.3061 | 1.07 | 0.949 | 0.949 |
| NDUFS1 | NADH dehydrogenase (ubiquinone) Fe-S protein 1, 75kDa (NADH-coenzyme Q reductase) | -2.72 | 0.072 | 0.267 | -2.31 | 0.092 | 0.234 |
| NDUFS2 | NADH dehydrogenase (ubiquinone) Fe-S protein 2, 49kDa (NADH-coenzyme Q reductase) | -6.47 | 0.045 | 0.254 | -16.72 | 0.037 | 0.156 |
| NDUFS3 | NADH dehydrogenase (ubiquinone) Fe-S protein 3, 30kDa (NADH-coenzyme Q reductase) | -1.78 | 0.052 | 0.254 | -2.27 | 0.020 | 0.149 |
| NDUFS4 | NADH dehydrogenase (ubiquinone) Fe-S protein 4, 18kDa (NADH-coenzyme Q reductase) | 1.04 | 0.690 | 0.772 | 1.08 | 0.624 | 0.718 |
| NDUFS5 | NADH dehydrogenase (ubiquinone) Fe-S protein 5, 15kDa (NADH-coenzyme Q reductase) | -1.65 | <0.01 | 0.165 | -1.33 | 0.027 | 0.156 |
| NDUFS6 | NADH dehydrogenase [ubiquinone] iron-sulfur protein 6, mitochondrial-like | -2.05 | 0.097 | 0.267 | -2.03 | 0.080 | 0.226 |
| NDUFS7 | NADH dehydrogenase [ubiquinone] iron-sulfur protein 7, mitochondrial-like | -1.34 | 0.176 | 0.306 | -2.02 | 0.039 | 0.156 |
| NDUFS8 | NADH dehydrogenase [ubiquinone] iron-sulfur protein 8, mitochondrial-like | -3.16^B^ | 0.174 | 0.306 | -2.31^B^ | 0.254 | 0.368 |
| NDUFV1 | NADH dehydrogenase [ubiquinone] flavoprotein 1, mitochondrial-like | -3.97^A^ | 0.087 | 0.267 | -5.14^A^ | 0.060 | 0.198 |
| PPA1 | Pyrophosphatase 1 | -1.58 | 0.104 | 0.267 | 1.30 | 0.332 | 0.436 |
| PPA2 | Inorganic pyrophosphatase 2, mitochondrial-like | -1.30 | 0.140 | 0.293 | -177.6^A^ | 0.303 | 0.418 |
| SDHA | Succinate dehydrogenase [ubiquinone] flavoprotein subunit, mitochondrial | -3.01 | 0.051 | 0.254 | -2.67 | 0.037 | 0.156 |
| SDHB | Succinate dehydrogenase complex, subunit B, iron sulfur (Ip) | -3.17 | 0.027 | 0.223 | -2.93 | 0.030 | 0.156 |
| SDHC | Succinate dehydrogenase cytochrome b560 subunit, mitochondrial-like | -1.28 | 0.561 | 0.646 | -1.19 | 0.419 | 0.533 |
| SDHD | Succinate dehydrogenase complex, subunit D, integral membrane protein | -1.24 | 0.447 | 0.529 | -1.35 | 0.027 | 0.156 |
| SLC25A25 | Solute carrier family 25 (mitochondrial carrier; phosphate carrier), member 25 | -3.33^A^ | 0.110 | 0.267 | -7.44^A^ | 0.074 | 0.223 |
| UCP2 | Uncoupling protein 2 (mitochondrial, proton carrier) | -7.34^B^ | 0.098 | 0.267 | -16.55^B^ | 0.088 | 0.230 |
| UQCR10 | Ubiquinol-cytochrome c reductase complex 7.2 kDa protein | 24.94 | 0.998 | 0.998 | 30.59 | 0.615 | 0.717 |

A: This gene's average threshold cycle is relatively high (> 30) in either the control or the test sample, and is reasonably low in the other sample (< 30). B: This gene's average threshold cycle is relatively high (> 30), meaning that its relative expression level is low, in both control and test samples, and the p-value for the fold-change is either unavailable or relatively high (p > 0.05). The FDR is the False Discovery Rate adjusted p-values

**S5 Table. Relative expression levels of the genes of the oxidative stress in intestinal mucosa in post-weaning (D23 and D35) in relation to the pre-weaning period (D-14)**

| Symbol | Description | (d23/14) | Fold d23 | FDR d23 | (d35/14) | Fold d35 | FDR d35 |
| --- | --- | --- | --- | --- | --- | --- | --- |
| ALB | Albumin | -4.35^B^ | 0.293 | 0.535 | 4.39^B^ | 0.163 | 0.350 |
| ALOX12 | Arachidonate 12-lipoxygenase | 2.50^A^ | 0.146 | 0.437 | -1.93^B^ | 0.072 | 0.251 |
| AOX1 | Aldehyde oxidase-like | -1.00^B^ | 0.873 | 0.945 | -2.17 | 0.017 | 0.120 |
| APOE | Apolipoprotein E | 3.52 | 0.260 | 0.495 | 1.00 | 0.898 | 0.943 |
| BAG2 | BAG family molecular chaperone regulator 2-like | 2.68 | 0.052 | 0.336 | 1.65 | 0.023 | 0.130 |
| BNIP3 | BCL2/adenovirus E1B 19 kDa protein-interacting protein 3-like | 1.34 | 0.437 | 0.568 | -1.35 | 0.309 | 0.499 |
| CAT | Catalase | 1.34 | 0.389 | 0.544 | -1.13 | 0.670 | 0.792 |
| CCL5 | Chemokine (C-C motif) ligand 5 | 1.76 | 0.176 | 0.456 | 2.09 | <0.01 | 0.049 |
| CYGB | Cytoglobin-like | 1.46 | 0.352 | 0.544 | -1.85 | <0.01 | 0.037 |
| DHCR24 | 24-dehydrocholesterol reductase | 1.85 | 0.377 | 0.544 | 1.05 | 0.500 | 0.675 |
| DUOX1 | Dual oxidase 1 | 2.22^B^ | 0.190 | 0.456 | 1.34^B^ | 0.396 | 0.572 |
| DUOX2 | Dual oxidase 2 | 15.35^B^ | 0.118 | 0.400 | 8.12^B^ | 0.097 | 0.263 |
| DUSP1 | Dual specificity phosphatase 1 | 1.20 | 0.889 | 0.945 | -2.17 | 0.138 | 0.331 |
| EPHX2 | Epoxide hydrolase 2, cytoplasmic | -1.08 | 0.756 | 0.836 | 1.79 | 0.219 | 0.391 |
| EPX | Eosinophil peroxidase | 46.73^B^ | 0.169 | 0.456 | 1.19^B^ | 0.888 | 0.943 |
| FHL2 | Four and a half LIM domains protein 2-like | 1.95 | 0.089 | 0.400 | -1.84^A^ | 0.038 | 0.159 |
| FOXM1 | Forkhead box M1 | 3.24^A^ | 0.049 | 0.336 | 1.03^B^ | 0.789 | 0.861 |
| FTH1 | Ferritin, heavy polypeptide 1 | 1.07 | 0.626 | 0.721 | -2.47 | <0.01 | 0.031 |
| GCLC | Glutamate-cysteine ligase, catalytic subunit | 1.85 | 0.115 | 0.400 | -1.38 | 0.176 | 0.367 |
| GCLM | Glutamate-cysteine ligase, modifier subunit | -1.02 | 0.925 | 0.963 | 1.96 | 0.967 | 0.986 |
| GLA | Alpha-galactosidase A | -2.42 | 0.127 | 0.409 | -2.72 | 0.055 | 0.212 |
| GP91-PHOX | NADPH oxidase heavy chain subunit | 5.91 | <0.01 | 0.289 | -1.32 | 0.084 | 0.263 |
| GPX1 | Glutathione peroxidase 1 | 2.50* | 0.023 | 0.326 | 1.06 | 0.785 | 0.861 |
| GPX2 | Glutathione peroxidase 2 (gastrointestinal) | 3.60 | 0.251 | 0.495 | 4.82 | <0.01 | 0.065 |
| GPX3 | Glutathione peroxidase 3 (plasma) | -3.89 | 0.378 | 0.544 | -21.73 | 0.226 | 0.396 |
| GPX4 | Glutathione peroxidase 4 (phospholipid hydroperoxidase) | -1.92 | 0.014 | 0.289 | -2.18 | <0.01 | 0.058 |
| GPX5 | Glutathione peroxidase 5 (epididymal androgen-related protein) | 2.61^B^ | 0.439 | 0.568 | 1.45^B^ | 0.975 | 0.986 |
| GSS | Glutathione synthetase | 1.23 | 0.390 | 0.544 | -1.69 | <0.01 | 0.049 |
| GSTZ1 | Glutathione S-transferase zeta 1 | -1.06 | 0.929 | 0.963 | -3.80^A^ | <0.01 | 0.037 |
| HMOX1 | Heme oxygenase (decycling) 1 | -1.43 | 0.458 | 0.571 | -5.30 | 0.037 | 0.159 |
| HSP70.2 | Heat shock protein 70.2 | 3.51 | 0.154 | 0.446 | 1.75 | 0.179 | 0.367 |
| HSP90AA1 | 90-kDa heat shock protein | 1.92 | 0.087 | 0.400 | -1.44 | 0.090 | 0.263 |
| KRT1 | Keratin 1 | 1.56^B^ | 0.471 | 0.573 | -1.25^B^ | 0.402 | 0.572 |
| LHPP | Phospholysine phosphohistidine inorganic pyrophosphate phosphatase | 3.24 | 0.184 | 0.456 | 1.22 | 0.726 | 0.824 |
| LOC100049683 | Solute carrier family 7 (anionic amino acid transporter light chain, xc- system), member 11 | 2.83 | 0.046 | 0.336 | 5.72^B^ | 0.137 | 0.331 |
| LOC100516395 | Lactoperoxidase-like | 2.29^B^ | 0.361 | 0.544 | 4.83^B^ | 0.206 | 0.385 |
| TTN | Titin-like | 2.52^B^ | 0.961 | 0.981 | -1.24^B^ | 0.091 | 0.263 |
| LOC100622575 | Pancreatic secretory trypsin inhibitor-like | 1.03 | 0.048 | 0.336 | 1.65 | 0.033 | 0.156 |
| LOC100739163 | Glutathione S-transferase P | 2.25 | 0.165 | 0.456 | 1.44 | <0.01 | 0.031 |
| LOC396625 | Glutathione reductase | 1.22 | 0.879 | 0.945 | 1.67 | 0.628 | 0.756 |
| LOC733635 | Aldo-keto reductase family 1 member C2-like | -1.05 | 0.417 | 0.556 | 1.05 | 0.319 | 0.506 |
| MB | Myoglobin | -1.58^B^ | 0.254 | 0.495 | -1.64^B^ | 0.389 | 0.572 |
| MBL2 | Mannose-binding lectin (protein C) 2, soluble | 2.80^B^ | 0.034 | 0.336 | 1.65^B^ | <0.01 | 0.031 |
| MGST3 | Microsomal glutathione S-transferase 3 | -1.56 | 0.011 | 0.289 | 1.40 | 0.191 | 0.374 |
| MPO | Myeloperoxidase | 24.57 | 0.325 | 0.544 | 4.30^B^ | 0.506 | 0.675 |
| MPV17 | MpV17 mitochondrial inner membrane protein | 2.55^B^ | 0.359 | 0.544 | 1.85^B^ | 0.741 | 0.829 |
| MSRA | Peptide methionine sulfoxide reductase | -1.40 | 0.074 | 0.400 | -1.03 | 0.992 | 0.992 |
| MT-III | Metallothionein-III | 29.99 | 0.017 | 0.290 | 1.14 | 0.196 | 0.374 |
| NCF1 | Neutrophil cytosolic factor 1 | 5.03* | 0.101 | 0.400 | -1.34 | 0.684 | 0.798 |
| NCF2 | Neutrophil cytosolic factor 2 | 3.12^A^ | 0.218 | 0.494 | 1.21^A^ | 0.137 | 0.331 |
| NCOA7 | Nuclear receptor coactivator 7 | 1.58 | 0.081 | 0.400 | -1.57 | 0.079 | 0.263 |
| NOS2 | Nitric oxide synthase 2, inducible | 7.61^A^ | 0.357 | 0.544 | 7.78^A^ | 0.026 | 0.130 |
| NOX4 | NADPH oxidase 4 | 1.62^B^ | 0.378 | 0.544 | -3.79^A^ | 0.143 | 0.331 |
| NOX5 | NADPH oxidase, EF-hand calcium binding domain 5 | 1.09^B^ | 0.116 | 0.400 | -2.87^B^ | 0.245 | 0.421 |
| NQO1 | NAD(P)H dehydrogenase, quinone 1 | 3.58 | 0.602 | 0.712 | 1.61 | 0.630 | 0.756 |
| NUDT1 | Nudix (nucleoside diphosphate linked moiety X)-type motif 1 | 1.22 | 0.301 | 0.538 | 1.17 | 0.146 | 0.331 |
| OXR1 | Oxidation resistance 1 | -1.39 | 0.462 | 0.571 | -1.45 | 0.016 | 0.120 |
| OXSR1 | Oxidative-stress responsive 1 | 1.24 | 0.333 | 0.544 | -1.74 | 0.250 | 0.421 |
| PDLIM1 | PDZ and LIM domain 1 | 1.64 | 0.401 | 0.544 | -1.25 | 0.846 | 0.911 |
| PRDX1 | Peroxiredoxin-1-like | -1.29 | 0.265 | 0.495 | 1.08 | 0.915 | 0.949 |
| PRDX2 | Peroxiredoxin 2 | -1.33 | <0.01 | 0.168 | 1.00 | 0.710 | 0.817 |
| PRDX3 | Peroxiredoxin 3 | -1.75 | 0.092 | 0.400 | 1.03 | 0.060 | 0.218 |
| PRDX4 | Peroxiredoxin 4 | -1.30 | 0.664 | 0.743 | 1.30 | 0.366 | 0.559 |
| PRDX5 | Peroxiredoxin 5 | -1.03 | 0.041 | 0.336 | -1.20 | 0.026 | 0.130 |
| PRDX6 | Peroxiredoxin 6 | -1.76 | 0.457 | 0.571 | -1.46 | 0.216 | 0.391 |
| PRNP | Prion protein | 1.46 | 0.215 | 0.494 | -1.32^A^ | 0.193 | 0.374 |
| PTGR1 | Prostaglandin reductase 1 | 2.23 | 0.119 | 0.400 | 2.23 | 0.161 | 0.350 |
| PTGS1 | Prostaglandin-endoperoxide synthase 1 (prostaglandin G/H synthase and cyclooxygenase) | 2.71 | 0.261 | 0.495 | -1.96^A^ | 0.517 | 0.679 |
| PTGS2 | Prostaglandin G/H synthase-2 | 4.40^A^ | 0.185 | 0.456 | -1.15^B^ | 0.092 | 0.263 |
| RNF7 | Ring finger protein 7 | -1.51 | 0.110 | 0.400 | -1.52 | 0.140 | 0.331 |
| SCARA3 | Scavenger receptor class A, member 3 | 2.95^B^ | 0.056 | 0.338 | -1.23^B^ | 0.019 | 0.120 |
| SEPP1 | Selenoprotein P, plasma, 1 | -3.26 | 0.341 | 0.544 | -1.67 | 0.023 | 0.130 |
| SFTPD | Surfactant protein D | 1.86 | 0.492 | 0.590 | 2.57 | <0.01 | 0.058 |
| SIRT2 | Sirtuin 2 | 1.19 | 0.971 | 0.981 | -2.19 | 0.097 | 0.263 |
| SOD1 | Superoxide dismutase 1, soluble | 1.01 | 0.138 | 0.430 | -1.43 | 0.410 | 0.574 |
| SOD3 | Extracellular superoxide dismutase | 5.91 | 0.315 | 0.544 | -1.07 | 0.331 | 0.515 |
| SQSTM1 | Sequestosome-1-like | 1.69 | 0.981 | 0.981 | -1.44 | 0.591 | 0.752 |
| TRAPPC6B | Trafficking protein particle complex subunit 6B-like | 1.05 | 0.232 | 0.495 | -1.07 | 0.376 | 0.564 |
| TTNLOC100620261 | Titin | -1.25^B^ | 0.611 | 0.713 | -2.16^B^ | 0.292 | 0.480 |
| TXN | Thioredoxin | 1.12 | 0.639 | 0.725 | 1.25 | 0.448 | 0.617 |
| TXNRD1 | Thioredoxin reductase 1 | 2.18 | 0.253 | 0.495 | 1.21 | 0.616 | 0.756 |
| TXNRD2 | Thioredoxin reductase 2 | 2.31 | 0.031 | 0.336 | -1.08 | 0.562 | 0.726 |
| UCP2 | Uncoupling protein 2 (mitochondrial, proton carrier) | 2.92^B^ | 0.249 | 0.495 | -2.62 | 0.046 | 0.186 |
| VIMP | Selenoprotein S | -1.39 | 0.398 | 0.544 | -1.06 | 0.614 | 0.756 |

A: This gene's average threshold cycle is relatively high (> 30) in either the control or the test sample, and is reasonably low in the other sample (< 30). B: This gene's average threshold cycle is relatively high (> 30), meaning that its relative expression level is low, in both control and test samples, and the p-value for the fold-change is either unavailable or relatively high (p > 0.05). The FDR is the False Discovery Rate adjusted p-values

**S6 Table. Relative expression levels of the genes of the energy metabolism in intestinal mucosa in post-weaning (D23 and D35) in relation to the pre-weaning period (D-14)**

| Symbol | Description | (d23/14) | Fold d23 | FDR d23 | (d35/14) | Fold d35 | FDR d35 |
| --- | --- | --- | --- | --- | --- | --- | --- |
| ARRDC3 | Arrestin domain containing 3 | -1.74 | 0.285 | 0.499 | -2.71 | 0.088 | 0.332 |
| ASB1 | Ankyrin repeat and SOCS box protein 1-like | 2.16 | 0.177 | 0.451 | -2.67^A^ | 0.053 | 0.323 |
| ATP12A | ATPase, H+/K+ transporting, nongastric, alpha polypeptide | 7.91^B^ | 0.092 | 0.451 | -1.52 | 0.049 | 0.323 |
| ATP4A | ATPase, H+/K+ exchanging, alpha polypeptide | 5.23^B^ | 0.129 | 0.451 | -1.12^B^ | 0.427 | 0.643 |
| ATP4B | ATPase, H+/K+ exchanging, beta polypeptide | 2.41^B^ | 0.194 | 0.451 | 9.00 | <0.01 | <0.01 |
| ATP5A1 | ATP synthase, H+ transporting, mitochondrial F1 complex, alpha subunit 1, cardiac muscle | -1.28 | 0.199 | 0.451 | -1.01 | 0.942 | 0.977 |
| ATP5B | ATP synthase, H+ transporting, mitochondrial F1 complex, beta polypeptide | -1.08 | 0.960 | 0.980 | 1.28 | 0.077 | 0.323 |
| ATP5E | ATP synthase, H+ transporting, mitochondrial F1 complex, epsilon subunit | -1.84 | 0.305 | 0.507 | 1.16 | 0.381 | 0.643 |
| ATP5F1 | ATP synthase, H+ transporting, mitochondrial Fo complex, subunit B1 | -1.34 | 0.559 | 0.637 | 1.58 | 0.208 | 0.500 |
| ATP5G1 | ATP synthase, H+ transporting, mitochondrial Fo complex, subunit C1 (subunit 9) | -1.04 | 0.996 | 0.996 | 1.34 | 0.059 | 0.323 |
| LOC100037988 | ATP synthase, H+ transporting, mitochondrial Fo complex, subunit C2 (subunit 9) | 2.45^B^ | 0.332 | 0.514 | -3.34^B^ | 0.768 | 0.898 |
| ATP5G3 | ATP synthase, H+ transporting, mitochondrial Fo complex, subunit C3 (subunit 9) | -2.03 | 0.168 | 0.451 | 1.01 | 0.418 | 0.643 |
| ATP5H | ATP synthase, H+ transporting, mitochondrial Fo complex, subunit d | -2.12 | 0.274 | 0.490 | -1.19 | 0.262 | 0.527 |
| ATP5I | ATP synthase, H+ transporting, mitochondrial Fo complex, subunit E | -2.14 | 0.494 | 0.618 | -1.62 | 0.256 | 0.527 |
| ATP5L | ATP synthase, H+ transporting, mitochondrial Fo complex, subunit G | -1.35 | 0.161 | 0.451 | -1.56 | 0.476 | 0.690 |
| ATP6V0A2 | ATPase, H+ transporting, lysosomal V0 subunit a2 | 1.34 | 0.267 | 0.490 | -1.31 | 0.017 | 0.323 |
| ATP6V0D2 | ATPase, H+ transporting, lysosomal 38kDa, V0 subunit d2 | 22.4 | 0.020 | 0.451 | -1.66^B^ | 0.146 | 0.395 |
| ATP6V1C2 | ATPase, H+ transporting, lysosomal 42kDa, V1 subunit C2 | 1.07 | 0.797 | 0.851 | -1.81 | 0.138 | 0.394 |
| ATP6V1E2 | ATPase, H+ transporting, lysosomal 31kDa, V1 subunit E2 | 8.49 | 0.039 | 0.451 | 1.17^B^ | 0.962 | 0.986 |
| ATP6V1G3 | V-type proton ATPase subunit G 3-like | 2.16^B^ | 0.1909 | 0.451 | -1.82^B^ | 0.115 | 0.394 |
| BCS1L | BCS1-like (S. cerevisiae) | 5.07^A^ | 0.177 | 0.451 | 2.41^A^ | 0.710 | 0.864 |
| Cox5b | Mitochondrial cytochrome c oxidase subunit Vb | -1.28 | 0.196 | 0.451 | 1.25 | 0.091 | 0.332 |
| COX6A1 | Cytochrome c oxidase subunit VIa polypeptide 1 | -1.65 | 0.300 | 0.507 | 1.14 | 0.569 | 0.759 |
| COX6C | COX6C protein | -3.18 | 0.110 | 0.451 | -1.21 | 0.283 | 0.528 |
| COX7A2 | COX7A2 protein | -2.36 | 0.215 | 0.473 | -1.53 | 0.270 | 0.527 |
| DNAJB1 | DnaJ (Hsp40) homolog, subfamily B, member 1 | 1.13 | 0.443 | 0.594 | -1.41 | <0.01 | 0.092 |
| EDN1 | Endothelin 1 | 2.73^A^ | 0.237 | 0.485 | -2.20 | 0.026 | 0.323 |
| GADD45B | Growth arrest and DNA-damage-inducible, beta | 1.82 | 0.177 | 0.451 | -1.49 | 0.137 | 0.394 |
| HSP70.2 | Heat shock protein 70.2 | 3.11 | 0.149 | 0.451 | 1.39 | 0.237 | 0.527 |
| LHPP | Phospholysine phosphohistidine inorganic pyrophosphate phosphatase | 2.61 | 0.196 | 0.451 | -1.26 | 0.308 | 0.550 |
| LOC100154992 | Mitochondrial inner membrane protein OXA1L-like | 1.92 | 0.454 | 0.594 | 1.312 | 0.264 | 0.527 |
| LOC100156375 | COX15 homolog, cytochrome c oxidase assembly protein | -1.42 | 0.134 | 0.451 | -1.16 | 0.262 | 0.527 |
| LOC100156879 | Ubiquinol--cytochrome c reductase | 2.04 | 0.516 | 0.629 | 1.40 | 0.432 | 0.643 |
| LOC100156967 | Cytochrome c oxidase subunit 5A, mitochondrial-like | -1.49 | 0.361 | 0.532 | -1.22 | 0.850 | 0.942 |
| LOC100157935 | Cytochrome c oxidase subunit 8C, mitochondrial-like | -4.16*^B^ | 0.332 | 0.514 | -3.23^B^ | 0.414 | 0.643 |
| LOC100511690 | Cytochrome c1, heme protein, mitochondrial-like | 1.54 | 0.140 | 0.451 | 1.32 | 0.084 | 0.332 |
| LOC100517408 | Cytochrome c oxidase subunit 7B2, mitochondrial-like | -15.74*^B^ | 0.598 | 0.670 | -1.31^B^ | 0.907 | 0.977 |
| LOC100519366 | Cytochrome c oxidase subunit 6B1-like | 3.70^B^ | 0.410 | 0.574 | -1.01^B^ | 0.436 | 0.643 |
| LOC100519594 | Cytochrome c oxidase subunit 6A2, mitochondrial-like | 1.92 | 0.268 | 0.490 | -1.02 | 0.780 | 0.898 |
| LOC100522725 | Cytochrome c oxidase subunit 8A, mitochondrial-like | -1.03 | 0.266 | 0.490 | -1.26 | 0.772 | 0.898 |
| LOC100624067 | Cytochrome c oxidase subunit 4 isoform 1, mitochondrial-like | -1.26 | 0.875 | 0.918 | -1.32 | 0.070 | 0.323 |
| LOC100624950 | Cytochrome b561 domain-containing protein 1-like | -1.73 | 0.091 | 0.451 | -3.22 | 0.063 | 0.323 |
| LOC100739238 | Low-density lipoprotein receptor-related protein 5-like | 1.61 | 0.356 | 0.532 | -3.31 | 0.121 | 0.394 |
| NDUFB6 | NADH dehydrogenase 1 beta subcomplex 6 | -1.87 | 0.459 | 0.594 | -1.01 | 0.063 | 0.323 |
| ATP5O | ATP synthase H+-transporting mitochondrial F1 complex O subunit | -2.61* | 0.135 | 0.451 | -1.12 | 0.074 | 0.323 |
| NDUFA1 | NADH dehydrogenase [ubiquinone] 1 alpha subcomplex subunit 1-like | -1.92 | 0.450 | 0.594 | -1.85 | 0.937 | 0.977 |
| NDUFA10 | NADH dehydrogenase [ubiquinone] 1 alpha subcomplex subunit 10, mitochondrial-like | 1.36 | 0.561 | 0.637 | 1.02 | 0.237 | 0.527 |
| NDUFA11 | NADH dehydrogenase (ubiquinone) 1 alpha subcomplex, 11, 14.7kDa | -1.29 | 0.323 | 0.514 | 1.27 | 0.870 | 0.949 |
| NDUFA2 | NADH dehydrogenase (ubiquinone) 1 alpha subcomplex, 2, 8kDa | -1.76 | 0.165 | 0.451 | 1.26 | 0.045 | 0.323 |
| NDUFA3 | NADH dehydrogenase [ubiquinone] 1 alpha subcomplex subunit 3-like | 1.43 | 0.079 | 0.451 | -1.24 | 0.068 | 0.323 |
| NDUFA4 | NADH dehydrogenase (ubiquinone) 1 alpha subcomplex, 4, 9kDa | -2.63 | 0.137 | 0.451 | -1.56 | 0.249 | 0.527 |
| NDUFA5 | NADH dehydrogenase [ubiquinone] 1 alpha subcomplex subunit 5-like | -2.13 | 0.042 | 0.451 | -1.27 | 0.135 | 0.394 |
| NDUFA6 | NADH dehydrogenase (ubiquinone) 1 alpha subcomplex, 6, 14kDa | 2.22^A^ | 0.163 | 0.451 | -1.49^B^ | 0.978 | 0.989 |
| NDUFA7 | NADH dehydrogenase [ubiquinone] 1 alpha subcomplex subunit 7-like | -1.64 | 0.386 | 0.558 | -1.00 | 1.000 | 1.000 |
| NDUFA8 | NADH dehydrogenase (ubiquinone) 1 alpha subcomplex, 8, 19kDa | -1.77 | 0.551 | 0.637 | 1.00 | 0.705 | 0.864 |
| NDUFA9 | NADH dehydrogenase [ubiquinone] 1 alpha subcomplex subunit 9, mitochondrial-like | 1.04^B^ | 0.800 | 0.851 | 1.76^B^ | 0.357 | 0.624 |
| NDUFAB1 | NADH dehydrogenase (ubiquinone) 1, alpha/beta subcomplex, 1, 8kDa | 1.04 | 0.531 | 0.629 | -1.21 | 0.061 | 0.323 |
| NDUFB10 | NADH dehydrogenase [ubiquinone] 1 beta subcomplex subunit 10-like | -1.28 | 0.069 | 0.451 | -1.27 | 0.131 | 0.394 |
| NDUFB2 | NADH dehydrogenase (ubiquinone) 1 beta subcomplex, 2, 8kDa | -3.09* | 0.530 | 0.629 | -1.23 | 0.039 | 0.323 |
| NDUFB3 | NADH dehydrogenase (ubiquinone) 1 beta subcomplex, 3, 12kDa | -1.88^B^ | 0.125 | 0.451 | 1.78^A^ | <0.01 | 0.168 |
| NDUFB4 | NADH dehydrogenase [ubiquinone] 1 beta subcomplex subunit 4-like | -1.32 | 0.661 | 0.721 | -2.26 | 0.282 | 0.528 |
| NDUFB5 | NADH dehydrogenase (ubiquinone) 1 beta subcomplex, 5, 16kDa | -1.02 | 0.065 | 0.451 | 1.55 | 0.828 | 0.940 |
| NDUFB7 | NADH dehydrogenase (ubiquinone) 1 beta subcomplex, 7, 18kDa | 11.9* | 0.033 | 0.451 | 1.33^B^ | 0.706 | 0.864 |
| NDUFB8 | NADH dehydrogenase (ubiquinone) 1 beta subcomplex, 8, 19kDa | -1.85 | 0.039 | 0.451 | 1.01 | 0.852 | 0.942 |
| NDUFB9 | NADH dehydrogenase (ubiquinone) 1 beta subcomplex, 9, 22kDa | -1.70 | 0.088 | 0.451 | -1.06 | 0.657 | 0.849 |
| NDUFC1 | NADH dehydrogenase (ubiquinone) 1, subcomplex unknown, 1, 6kDa | -2.88 | 0.101 | 0.451 | -1.34 | 0.206 | 0.500 |
| NDUFS1 | NADH dehydrogenase (ubiquinone) Fe-S protein 1, 75kDa (NADH-coenzyme Q reductase) | 1.01 | 0.912 | 0.946 | 1.40 | 0.161 | 0.414 |
| NDUFS2 | NADH dehydrogenase (ubiquinone) Fe-S protein 2, 49kDa (NADH-coenzyme Q reductase) | 3.69^B^ | 0.257 | 0.490 | -1.34^B^ | 0.163 | 0.414 |
| NDUFS3 | NADH dehydrogenase (ubiquinone) Fe-S protein 3, 30kDa (NADH-coenzyme Q reductase) | 1.29 | 0.337 | 0.514 | 1.16 | 0.564 | 0.759 |
| NDUFS4 | NADH dehydrogenase (ubiquinone) Fe-S protein 4, 18kDa (NADH-coenzyme Q reductase) | -1.79 | 0.220 | 0.473 | -1.02 | 0.737 | 0.884 |
| NDUFS5 | NADH dehydrogenase (ubiquinone) Fe-S protein 5, 15kDa (NADH-coenzyme Q reductase) | -2.52 | 0.115 | 0.451 | -1.01 | 0.934 | 0.977 |
| NDUFS6 | NADH dehydrogenase [ubiquinone] iron-sulfur protein 6, mitochondrial-like | -1.15 | 0.968 | 0.980 | -1.08 | 0.495 | 0.705 |
| NDUFS7 | NADH dehydrogenase [ubiquinone] iron-sulfur protein 7, mitochondrial-like | -1.20 | 0.627 | 0.693 | -1.82 | 0.038 | 0.323 |
| NDUFS8 | NADH dehydrogenase [ubiquinone] iron-sulfur protein 8, mitochondrial-like | 1.16^B^ | 0.475 | 0.605 | 1.10^B^ | 0.418 | 0.643 |
| NDUFV1 | NADH dehydrogenase [ubiquinone] flavoprotein 1, mitochondrial-like | 2.58^A^ | 0.180 | 0.451 | 1.49^A^ | 0.545 | 0.759 |
| PPA1 | Pyrophosphatase 1 | -1.58 | 0.232 | 0.485 | 1.37 | 0.064 | 0.323 |
| PPA2 | Inorganic pyrophosphatase 2, mitochondrial-like | -1.41 | 0.308 | 0.507 | -1.10 | 0.683 | 0.864 |
| SDHA | Succinate dehydrogenase [ubiquinone] flavoprotein subunit, mitochondrial | 1.23 | 0.500 | 0.618 | -1.21 | 0.294 | 0.537 |
| SDHB | Succinate dehydrogenase complex, subunit B, iron sulfur (Ip) | -1.37 | 0.401 | 0.571 | 1.05 | 0.593 | 0.778 |
| SDHC | Succinate dehydrogenase cytochrome b560 subunit, mitochondrial-like | -1.86 | <0.01 | 0.315 | -1.23 | 0.057 | 0.323 |
| SDHD | Succinate dehydrogenase complex, subunit D, integral membrane protein | -1.24 | 0.254 | 0.490 | -1.35 | 0.398 | 0.643 |
| SLC25A25 | Solute carrier family 25 (mitochondrial carrier; phosphate carrier), member 25 | 2.53 | 0.1404 | 0.451 | -1.10 | 0.567 | 0.759 |
| UCP2 | Uncoupling protein 2 (mitochondrial, proton carrier) | 5.59^B^ | 0.1924 | 0.451 | -1.36^B^ | 0.384 | 0.643 |
| UQCR10 | Ubiquinol-cytochrome c reductase complex 7.2 kDa protein | -2.14 | 0.4424 | 0.594 | 1.38 | 0.141 | 0.394 |

A: This gene's average threshold cycle is relatively high (> 30) in either the control or the test sample, and is reasonably low in the other sample (< 30). B: This gene's average threshold cycle is relatively high (> 30), meaning that its relative expression level is low, in both control and test samples, and the p-value for the fold-change is either unavailable or relatively high (p > 0.05). The FDR is the False Discovery Rate adjusted p-values

**S7 Table. Relative expression levels of the genes of cytokines in intestinal mucosa in post-weaning (D23 and D35) in relation to the pre-weaning period (D-14)**

| Symbol | Description | (d23/14) | Fold d23 | FDR d23 | (d35/14) | Fold d35 | FDR d35 |
| --- | --- | --- | --- | --- | --- | --- | --- |
| ADIPOQ | Adiponectin, C1Q and collagen domain containing | 3.49^B^ | 0.095 | 0.184 | -1.62^B^ | 0.096 | 0.184 |
| AMCF-II | Alveolar macrophage-derived chemotactic factor-II | 1.14 | 0.664 | 0.734 | 2.71 | 0.113 | 0.201 |
| BMP2 | Bone morphogenetic protein 2 | 1.51 | 0.049 | 0.132 | -1.28 | 0.737 | 0.774 |
| BMP3 | Bone morphogenetic protein 3 | 1.45 | 0.106 | 0.193 | -2.57 | 0.005 | 0.024 |
| BMP4 | Bone morphogenetic protein 4 | 1.13 | 0.404 | 0.509 | -1.23 | 0.034 | 0.090 |
| BMP6 | Bone morphogenetic protein 6 | 1.14^B^ | 0.839 | 0.880 | -2.95^B^ | 0.115 | 0.201 |
| Bmp7 | Bone morphogenetic protein 7 | 1.89^A^ | 0.117 | 0.201 | -1.24^B^ | 0.746 | 0.774 |
| C5 | Complement component 5 | 1.92 | 0.154 | 0.235 | 2.42 | <0.01 | <0.01 |
| CCL1 | Chemokine (C-C motif) ligand 1 | 8.52 | <0.01 | <0.01 | 1.22^B^ | 0.220 | 0.313 |
| CCL11 | CCL11 | -1.87 | 0.050 | 0.132 | 1.04 | 0.969 | 0.969 |
| CCL17 | Chemokine ligand 17-like protein | 29.41^A^ | 0.133 | 0.207 | 1.54^B^ | 0.158 | 0.255 |
| CCL19 | Chemokine (C-C motif) ligand 19 | 7.92 | <0.01 | <0.01 | 2.38 | 0.211 | 0.306 |
| CCL2 | Chemokine (C-C motif) ligand 2 | 1.80 | <0.01 | <0.01 | 1.25 | 0.016 | 0.058 |
| CCL20 | Chemokine (C-C motif) ligand 20 | -2.09 | 0.030 | 0.089 | -2.93 | 0.142 | 0.239 |
| CCL21 | Chemokine (C-C motif) ligand 21 | 1.15 | 0.193 | 0.280 | -2.97 | 0.136 | 0.234 |
| CCL22 | C-C motif chemokine 22-like | 9.30 | 0.492 | 0.585 | -1.53^B^ | 0.029 | 0.078 |
| CCL25 | Chemokine (C-C motif) ligand 25 | -5.21 | 0.022 | 0.070 | -1.39 | 0.430 | 0.509 |
| CCL27 | Chemokine (C-C motif) ligand 27 | 1.80^A^ | 0.014 | 0.057 | 1.51^A^ | 0.290 | 0.375 |
| CCL28 | Chemokine (C-C motif) ligand 28 | 1.04 | 0.086 | 0.181 | 3.37 | <0.01 | 0.011 |
| CCL3L1 | Chemokine (C-C motif) ligand 3-like 1 | 8.28^A^ | 0.061 | 0.146 | 3.63^A^ | 0.042 | 0.106 |
| CCL4 | Chemokine (C-C motif) ligand 4 | 9.14^A^ | 0.120 | 0.201 | 4.56^A^ | <0.01 | 0.002 |
| CCL5 | Chemokine (C-C motif) ligand 5 | -1.03 | 0.673 | 0.734 | 1.48 | 0.028 | 0.078 |
| CCL8 | Chemokine (C-C motif) ligand 8 | 5.21 | 0.019 | 0.068 | 2.38 | 0.051 | 0.115 |
| CD40LG | CD40 ligand | 1.35^A^ | 0.020 | 0.068 | 1.12^B^ | 0.048 | 0.111 |
| CSF1 | Colony stimulating factor 1 (macrophage) | 3.34^A^ | 0.70 | 0.757 | 1.24^B^ | <0.01 | 0.002 |
| CSF2 | Colony stimulating factor 2 (granulocyte-macrophage) | -1.42^B^ | <0.01 | <0.01 | -1.83 | 0.082 | 0.160 |
| CSF3 | Colony stimulating factor 3 (granulocyte) | 3.34 | 0.047 | 0.132 | -1.71 | 0.498 | 0.565 |
| CXCL10 | Chemokine (C-X-C motif) ligand 10 | 6.25 | 0.340 | 0.440 | 6.35 | <0.01 | 0.026 |
| CXCL11 | Chemokine (C-X-C motif) ligand 11 | 20.45^A^ | 0.073 | 0.161 | 12.9^A^ | 0.271 | 0.362 |
| CXCL12 | Chemokine (C-X-C motif) ligand 12 | 1.08 | 0.132 | 0.207 | -1.17 | 0.047 | 0.111 |
| LOC396594 | Growth-regulated protein homolog gamma | -1.48 | <0.01 | <0.01 | -1.48 | <0.01 | <0.01 |
| CXCL9 | Chemokine (C-X-C motif) ligand 9 | 10.4 | 0.117 | 0.201 | 8.17 | <0.01 | <0.01 |
| FASLG | Fas ligand (TNF superfamily, member 6) | -1.31^B^ | 0.116 | 0.201 | 1.07^B^ | <0.01 | 0.030 |
| IFN-ALPHA-4 | Interferon-alpha-4 | 1.55^B^ | 0.282 | 0.375 | -2.69 | 0.062 | 0.137 |
| IFN-ALPHA-5 | Interferon, alpha 5 | 2,76^A^ | 0.128 | 0.206 | -2.05 | 0.019 | 0.065 |
| IFNB1 | Interferon beta | 14.52 | 0.318 | 0.417 | 1.98^B^ | 0.442 | 0.516 |
| IFNG | Interferon-gamma | 2.31^B^ | 0.095 | 0.184 | 19.16^A^ | <0.01 | 0.026 |
| IL10 | Interleukin 10 | -1.00 | 0.425 | 0.524 | -1.15 | 0.509 | 0.570 |
| IL12A | Interleukin 12A (natural killer cell stimulatory factor 1, cytotoxic lymphocyte maturation factor 1, p35) | 9.60 | 0.198 | 0.282 | -1.64^B^ | <0.01 | <0.01 |
| IL12B | Interleukin 12B (natural killer cell stimulatory factor 2, cytotoxic lymphocyte maturation factor 2, p40) | 33.47 | 0.064 | 0.149 | 10.40 | <0.01 | 0.013 |
| IL13 | Interleukin 13 | 4.74 | <0.01 | <0.01 | -1.55^B^ | 0.185 | 0.283 |
| IL15 | Interleukin 15 | -1.45 | 0.088 | 0.181 | 1.35 | <0.01 | <0.01 |
| IL16 | Interleukin 16 | 2.57 | 0.849 | 0.880 | 1.06 | 0.339 | 0.418 |
| IL17A | Interleukin 17A | 2.09 | <0.01 | 0.016 | 4.67 | 0.717 | 0.774 |
| IL17F | Interleukin 17F | -1.39^B^ | 0.036 | 0.104 | 5.66 | <0.01 | 0.018 |
| IL18 | Interleukin 18 (interferon-gamma-inducing factor) | 1.21 | 0.014 | 0.057 | 2.09 | 0.193 | 0.284 |
| IL1A | Interleukin 1, alpha | 1.97^A^ | <0.01 | <0.01 | 1.67^A^ | 0.068 | 0.142 |
| IL1B1 | Interleukin 1, beta 1 | 3.54^A^ | <0.01 | 0.016 | 1.35^B^ | 0.703 | 0.774 |
| IL2 | Interleukin 2 | 25.63^B^ | <0.01 | <0.01 | 18.86 | <0.01 | <0.01 |
| IL21 | Interleukin 21 | 52.72^B^ | 0.267 | 0.367 | 264.38 | 0.028 | 0.078 |
| IL22 | Interleukin 22 | 1.04^B^ | 0.495 | 0.585 | 10.10 | <0.01 | 0.019 |
| IL23A | Interleukin 23, alpha subunit p19 | 1.99^B^ | 0.052 | 0.132 | -1.10^B^ | 0.243 | 0.335 |
| IL27 | Interleukin 27 | 5.50^B^ | 0.018 | 0.065 | 1.32^B^ | 0.283 | 0.371 |
| IL4 | Interleukin 4 | 2.93 | 0.097 | 0.184 | -1.68^B^ | 0.022 | 0.067 |
| IL5 | Interleukin 5 | -1.03^A^ | 0.058 | 0.143 | -1.54^B^ | <0.01 | <0.01 |
| IL6 | Interleukin 6 (interferon, beta 2) | 10.14 | 0.921 | 0.943 | 2.74^B^ | 0.022 | 0.067 |
| IL7 | Interleukin 7 | -1.23 | 0.187 | 0.275 | 1.12 | 0.723 | 0.774 |
| CXCL8 | Interleukin 8 | -1.36 | 0.087 | 0.181 | 1.46 | 0.325 | 0.407 |
| IL9 | Interleukin 9 | -1.38^B^ | 0.017 | 0.064 | -1.15^B^ | 0.190 | 0.284 |
| INHA | Inhibin, alpha | -1.92^C^ | 0.666 | 0.734 | -1.31^C^ | 0.067 | 0.142 |
| INHBA | Inhibin, beta A | 1.00^A^ | <0.01 | <0.01 | -2.98 | 0.070 | 0.143 |
| LIF | Leukemia inhibitory factor (cholinergic differentiation factor) | 1.91^A^ | <0.01 | 0.022 | -1.40^B^ | 0.045 | 0.111 |
| OSM | Oncostatin-M-like | 2.85^B^ | 0.596 | 0.686 | -1.31^C^ | 0.761 | 0.779 |
| LOC100515857 | C-C motif chemokine 3-like | -1.23^B^ | <0.01 | 0.024 | 1.80 | 0.020 | 0.066 |
| CCL23 | C-C motif chemokine 23-like | 10.41^A^ | 0.590 | 0.686 | 2.18^B^ | <0.01 | <0.01 |
| CCL16 | C-C motif chemokine 16-like | -45.74^A^ | 0.099 | 0.184 | -9.86^A^ | 0.158 | 0.255 |
| CNTF | Ciliary neurotrophic factor-like | 1.21^A^ | 0.465 | 0.566 | -2.26 | <0.01 | 0.013 |
| CXCL13 | C-X-C motif chemokine 13-like | 12.55^A^ | 0.406 | 0.509 | 4.15^A^ | 0.415 | 0.498 |
| THPO | Thrombopoietin | 1.61^B^ | 0.243 | 0.341 | 2.07^B^ | 0.263 | 0.357 |
| LOC100621682 | Uncharacterized LOC100621682 | 1.62^B^ | <0.01 | 0.029 | -1.32^B^ | 0.226 | 0.316 |
| TNFSF9 | Tumor necrosis factor ligand superfamily member 9-like | 1.99 | <0.01 | <0.01 | 1.26^B^ | 0.165 | 0.262 |
| CCL24 | Chemokine ligand 24-like protein | 10.22^A^ | 0.989 | 0.989 | 11.47^A^ | 0.169 | 0.263 |
| LTA | Lymphotoxin alpha (TNF superfamily, member 1) | 22.80 | 0.281 | 0.375 | -2.25^B^ | 0.397 | 0.483 |
| LTB | Lymphotoxin beta (TNF superfamily, member 3) | 10.70 | 0.071 | 0.161 | 1.52 | 0.020 | 0.066 |
| MIF | Macrophage migration inhibitory factor (glycosylation-inhibiting factor) | 1.02 | <0.01 | <0.01 | -1.23 | 0.111 | 0.201 |
| MSTN | Myostatin | 1.19^B^ | <0.01 | 0.041 | -1.66^B^ | 0.791 | 0.801 |
| SPP1 | Secreted phosphoprotein 1 | 141.50^A^ | 0.127 | 0.206 | 10.97^A^ | <0.01 | 0.025 |
| TGFB1 | Transforming growth factor, beta 1 | 3.68 | 0.740 | 0.786 | -1.01 | 0.319 | 0.407 |
| TGFB2 | Transforming growth factor, beta 2 | -1.34 | <0.01 | 0.0242 | -2.73^A^ | 0.074 | 0.147 |
| TNF | Tumor necrosis factor | 1.50 | 0.656 | 0.734 | -1.34 | <0.01 | <0.01 |
| TNFSF10 | Tumor necrosis factor (ligand) superfamily, member 10 | -1.04 | <0.01 | <0.01 | 1.65 | <0.01 | <0.01 |
| TNFSF13B | Tumor necrosis factor (ligand) superfamily, member 13b | 11.49 | 0.178 | 0.266 | 29.53 | 0.729 | 0.774 |
| TNFSF4 | Tumor necrosis factor (ligand) superfamily, member 4 | 1.52^B^ | 0.025 | 0.0777 | -1.22^B^ | 0.478 | 0.550 |
| VEGFA | Vascular endothelial growth factor A | 1.09 | 0.966 | 0.977 | -1.87 | 0.111 | 0.201 |

A: This gene's average threshold cycle is relatively high (> 30) in either the control or the test sample, and is reasonably low in the other sample (< 30). B: This gene's average threshold cycle is relatively high (> 30), meaning that its relative expression level is low, in both control and test samples, and the p-value for the fold-change is either unavailable or relatively high (p > 0.05). The FDR is the False Discovery Rate adjusted p-values

**S8 Table. Relative expression levels of the genes of the apoptosis in intestinal mucosa in post-weaning (D23 and D35) in relation to the pre-weaning period (D-14)**

| Symbol | Description | (d23/14) | Fold d23 | FDR d23 | (d35/14) | Fold d35 | FDR d35 |
| --- | --- | --- | --- | --- | --- | --- | --- |
| ABL1 | C-abl oncogene 1, non-receptor tyrosine kinase | 1.76 | 0.217 | 0.535 | -3.15^A^ | <0.01 | 0.012 |
| AIFM1 | Apoptosis-inducing factor, mitochondrion-associated, 1 | 1.03 | 0.748 | 0.923 | -1.22 | <0.01 | 0.017 |
| AIFM3 | Apoptosis-inducing factor, mitochondrion-associated, 3 | 1.25^B^ | 0.967 | 0.988 | -1.74^B^ | 0.171 | 0.256 |
| AKT1 | V-akt murine thymoma viral oncogene homolog 1 | 1.75 | 0.122 | 0.449 | -1.86 | <0.01 | 0.015 |
| APAF1 | Apoptotic peptidase activating factor 1 | -1.17 | 0.508 | 0.765 | -2.57 | 0.045 | 0.104 |
| BAD | BCL2-associated agonist of cell death | 1.69 | 0.162 | 0.469 | -1.34^A^ | 0.119 | 0.204 |
| BAG3 | BCL2-associated athanogene 3 | -1.54 | 0.416 | 0.698 | -6.69^A^ | <0.01 | 0.035 |
| BAK1 | Bak protein | 1.39 | 0.302 | 0.597 | -1.19 | 0.040 | 0.098 |
| BAX | BCL2-associated X protein | -1.31 | 0.428 | 0.702 | -1.34 | 0.160 | 0.245 |
| BCL2 | B-cell CLL/lymphoma 2 | 2.32 | 0.158 | 0.469 | -1.29 | <0.01 | 0.012 |
| BCL2A1 | BCL2-related protein A1 | 5.27 | 0.050 | 0.352 | 2.94 | <0.01 | 0.015 |
| BCL2L1 | BCL2-like 1 | 1.39 | 0.699 | 0.917 | -1.15 | 0.540 | 0.597 |
| BCL2L10 | BCL2-like 10 (apoptosis facilitator) | 3.18^B^ | 0.292 | 0.597 | -2.09^B^ | 0.304 | 0.381 |
| LOC100154044 | BCL2-like 2 | 1.18 | 0.773 | 0.923 | -2.82 | <0.01 | 0.022 |
| BID | BH3 interacting domain death agonist | -1.08 | 0.675 | 0.917 | -1.48 | 0.756 | 0.804 |
| LOC100622859 | Baculoviral IAP repeat containing 2 | 1.08 | 0.510 | 0.765 | -1.40 | <0.01 | 0.014 |
| BIRC3 | Baculoviral IAP repeat containing 3 | -1.09 | 0.899 | 0.973 | 1.15 | 0.348 | 0.418 |
| BIRC5 | Baculoviral IAP repeat containing 5 | 1.21 | 0.990 | 0.990 | 1.84 | 0.033 | 0.093 |
| BNIP2 | BCL2/adenovirus E1B 19kDa interacting protein 2 | -1.02^B^ | 0.019 | 0.352 | -1.14^B^ | <0.01 | 0.014 |
| BNIP3L | BCL2/adenovirus E1B 19kDa interacting protein 3-like | -1.56 | 0.753 | 0.923 | -1.58 | 0.012 | 0.046 |
| BRAF | V-raf murine sarcoma viral oncogene homolog B1 | 1.05 | 0.344 | 0.627 | -1.64 | <0.01 | 0.014 |
| CASP1 | Caspase 1, apoptosis-related cysteine peptidase (interleukin 1, beta, convertase) | -1.17 | 0.780 | 0.923 | 2.08 | 0.013 | 0.046 |
| CASP10 | Caspase 10, apoptosis-related cysteine peptidase | -1.20 | 0.325 | 0.607 | -2.06 | 0.945 | 0.956 |
| CASP14 | Caspase 14, apoptosis-related cysteine peptidase | 4.06^B^ | 0.058 | 0.352 | 1.00^C^ | 0.322 | 0.397 |
| CASP2 | Caspase 2, apoptosis-related cysteine peptidase | 2.34 | <0.01 | 0.029 | -1.26 | 0.126 | 0.204 |
| CASP3 | Caspase 3, apoptosis-related cysteine peptidase | -1.51 | 0.250 | 0.583 | -1.25 | 0.610 | 0.656 |
| LOC100522887 | Caspase 4, apoptosis-related cysteine peptidase | -1.02 | 0.035 | 0.352 | 1.47 | 0.010 | 0.042 |
| CASP6 | Caspase 6, apoptosis-related cysteine peptidase | -1.20 | 0.351 | 0.627 | 1.05 | 0.057 | 0.124 |
| CASP8 | Caspase 8, apoptosis-related cysteine peptidase | -1.29 | 0.491 | 0.763 | 1.23 | 0.034 | 0.093 |
| CBX4 | Chromobox homolog 4 | 1.32 | <0.01 | 0.029 | -2.58^A^ | <0.01 | 0.033 |
| CD40 | CD40 molecule, TNF receptor superfamily member 5 | 4.81 | 0.058 | 0.352 | 1.34 | 0.253 | 0.348 |
| CD40LG | CD40 ligand | 1.37^A^ | 0.095 | 0.442 | -1.06^B^ | 0.181 | 0.258 |
| CD70 | CD70 molecule | 7.03^B^ | 0.886 | 0.973 | 1.99^B^ | 0.123 | 0.204 |
| CFDP1 | Craniofacial development protein 1 | -1.02 | 0.927 | 0.973 | -1.32 | 0.275 | 0.363 |
| CIDEA | Cell death-inducing DFFA-like effector a | -1.32^B^ | 0.916 | 0.973 | -1.78^B^ | <0.01 | 0.014 |
| CIDEB | Cell-death-inducing DNA-fragmentation-factor-like effector B | -1.02 | 0.283 | 0.597 | -2.62 | 0.333 | 0.405 |
| CUL2 | Cullin 2 | 1.17 | 0.976 | 0.988 | 1.18 | 0.518 | 0.588 |
| CYCS | Cytochrome c, somatic | -1.51 | 0.127 | 0.449 | -1.42 | <0.01 | 0.018 |
| LOC100516103 | Death-associated protein kinase 1 | 3.41 | 0.717 | 0.923 | -1.47 | 0.281 | 0.363 |
| DDX20 | DEAD (Asp-Glu-Ala-Asp) box polypeptide 20 | 1.64 | 0.094 | 0.442 | -1.57 | 0.880 | 0.901 |
| DIABLO | Diablo, IAP-binding mitochondrial protein | -1.18 | 0.025 | 0.352 | -1.01 | 0.566 | 0.617 |
| DPF2 | D4, zinc and double PHD fingers family 2 | 1.84 | 0.659 | 0.917 | -1.03 | 0.126 | 0.204 |
| ERC1 | ELKS/RAB6-interacting/CAST family member 1 | -1.11 | 0.144 | 0.449 | -5.05^A^ | 0.100 | 0.181 |
| ERN2 | Endoplasmic reticulum to nucleus signaling 2 | 1.86^A^ | 0.022 | 0.352 | -1.62^B^ | <0.01 | 0.014 |
| FAS | Fas (TNF receptor superfamily, member 6) | 1.98 | 0.925 | 0.973 | 1.76 | 0.010 | 0.042 |
| FASLG | Fas ligand (TNF superfamily, member 6) | -1.11^B^ | 0.880 | 0.973 | 1.60 | <0.01 | 0.033 |
| FEM1B | Fem-1 homolog b (C. elegans) | 1.02 | 0.753 | 0.923 | -1.79 | 0.178 | 0.258 |
| GADD45A | Growth arrest and DNA-damage-inducible, alpha | -1.17 | 0.571 | 0.827 | -1.45 | 0.068 | 0.136 |
| IGF1R | Insulin-like growth factor 1 receptor | 1.46 | 0.313 | 0.597 | -4.88 | <0.01 | 0.022 |
| IL10 | Interleukin 10 | -1.95^A^ | 0.200 | 0.535 | 1.01 | 0.847 | 0.879 |
| LALBA | Lactalbumin, alpha- | -2.64^B^ | 0.377 | 0.657 | -2.40^B^ | 0.297 | 0.378 |
| HRK | Activator of apoptosis harakiri-like | -1.12^B^ | 0.679 | 0.917 | -4.60^B^ | 0.030 | 0.093 |
| LOC100156777 | Caspase 7 | 1.00 | 0.853 | 0.973 | 1.03 | 0.776 | 0.815 |
| TNFRSF21 | Tumor necrosis factor receptor superfamily member 21-like | 1.39 | 0.060 | 0.352 | -1.87 | 0.046 | 0.104 |
| RIPK2 | Receptor-interacting serine/threonine-protein kinase 2-like | -1.49 | 0.050 | 0.352 | -1.22 | 0.104 | 0.185 |
| CASP9 | Caspase-9-like | 1.37^B^ | 0.049 | 0.352 | -1.94^B^ | <0.01 | 0.012 |
| LTBR | Tumor necrosis factor receptor superfamily member 3-like | 1.56 | 0.952 | 0.987 | -1.35 | <0.01 | 0.017 |
| LOC100522011 | Apoptosis-associated speck-like protein containing a CARD-like | -1.38 | 0.435 | 0.702 | 2.09 | 0.078 | 0.146 |
| DFFA | DNA fragmentation factor subunit alpha-like | -1.00 | 0.688 | 0.917 | -1.48 | 0.071 | 0.138 |
| LOC100523672 | Nucleolar protein 3-like | -1.25^B^ | 0.139 | 0.449 | -1.79^B^ | <0.01 | 0.033 |
| BNIP3 | BCL2/adenovirus E1B 19 kDa protein-interacting protein 3-like | -1.35^A^ | 0.063 | 0.352 | -3.16^A^ | 0.042 | 0.101 |
| LOC641352 | Caspase-15 | 1.82 | 0.311 | 0.597 | -1.73 | 0.264 | 0.358 |
| LTA | Lymphotoxin alpha (TNF superfamily, member 1) | 10.33^B^ | 0.646 | 0.917 | -4.11* | 0.473 | 0.544 |
| MCL1 | Myeloid cell leukemia sequence 1 (BCL2-related) | 1.05 | 0.107 | 0.449 | -1.07 | 0.033 | 0.093 |
| MTL5 | Metallothionein-like 5, testis-specific (tesmin) | 3.47^B^ | 0.141 | 0.449 | 2.24 | 0.161 | 0.245 |
| NFKB1 | Nuclear factor of kappa light polypeptide gene enhancer in B-cells 1 | 2.15 | 0.127 | 0.449 | -1.12 | 0.957 | 0.957 |
| NOD1 | Nucleotide-binding oligomerization domain containing 1 | 6.1B^B^ | 0.210 | 0.535 | 1.17^B^ | 0.128 | 0.204 |
| PAK7 | P21 protein (Cdc42/Rac)-activated kinase 7 | -5.87^B^ | 0.205 | 0.535 | -7.39^B^ | 0.036 | 0.095 |
| PPP2R1A | Protein phosphatase 2, regulatory subunit A, alpha | 2.10 | 0.872 | 0.973 | -2.75^A^ | 0.040 | 0.098 |
| PPP2R1B | Protein phosphatase 2, regulatory subunit A, beta | -1.03 | 0.567 | 0.827 | -1.70 | 0.528 | 0.592 |
| PROP1 | PROP paired-like homeobox 1 | 1.31^B^ | 0.865 | 0.973 | -1.08^B^ | 0.033 | 0.093 |
| RFWD2 | Ring finger and WD repeat domain 2 | -1.05 | 0.283 | 0.597 | -1.49 | 0.184 | 0.258 |
| SART1 | Squamous cell carcinoma antigen recognized by T cells | 2.33 | 0.125 | 0.449 | -1.96 | 0.019 | 0.062 |
| LOC100517325 | Signal-induced proliferation-associated 1 | 3.76^B^ | 0.059 | 0.352 | -1.91^B^ | 0.064 | 0.131 |
| STAMBP | STAM binding protein | -1.17 | 0.223 | 0.535 | -1.34 | 0.059 | 0.124 |
| TNF | Tumor necrosis factor | 1.61 | 0.218 | 0.535 | -1.95 | 0.181 | 0.258 |
| TNFRSF11B | Tumor necrosis factor receptor superfamily, member 11b | 3.03^B^ | 0.307 | 0.597 | -1.31^B^ | 0.024 | 0.077 |
| TNFRSF1A | Tumor necrosis factor receptor superfamily, member 1A | 1.36 | 0.057 | 0.352 | -1.73^A^ | 0.110 | 0.192 |
| TNFRSF1B | Tumor necrosis factor receptor superfamily, member 1B | 4.43 | 0.443 | 0.702 | -1.15 | 0.049 | 0.108 |
| TNFSF10 | Tumor necrosis factor (ligand) superfamily, member 10 | -1.46 | 0.383 | 0.657 | 1.24 | 0.281 | 0.363 |
| TP53 | Tumor protein p53 | 1.84 | 0.139 | 0.449 | -1.07 | 0.381 | 0.445 |
| TP53BP2 | Tumor protein p53 binding protein, 2 | 1.61 | 0.289 | 0.597 | -1.69* | 0.013 | 0.0458 |
| TRAF2 | TNF receptor-associated factor 2 | 2.34 | 0.086 | 0.442 | -1.58 | 0.072 | 0.138 |
| XIAP | X-linked inhibitor of apoptosis | -1.16 | 0.778 | 0.923 | -1.16 | 0.353 | 0.418 |

A: This gene's average threshold cycle is relatively high (> 30) in either the control or the test sample, and is reasonably low in the other sample (< 30). B: This gene's average threshold cycle is relatively high (> 30), meaning that its relative expression level is low, in both control and test samples, and the p-value for the fold-change is either unavailable or relatively high (p > 0.05). The FDR is the False Discovery Rate adjusted p-values

**S9 Table. RT² Profiler™ PCR Array Pig Oxidative Stress CT values in the liver of 14, 23 and 35 days old normal birth weight piglets**

| Well | Symbol | Description | D14 | D23 | D35 |
| --- | --- | --- | --- | --- | --- |
| A01 | ALB | Albumin | 13.57 | 14.05 | 15.31 |
| A02 | ALOX12 | Arachidonate 12-lipoxygenase | 31.58 | 33.22 | 33.73 |
| A03 | AOX1 | Aldehyde oxidase-like | 25.34 | 24.36 | 24.39 |
| A04 | APOE | Apolipoprotein E | 19.99 | 19.85 | 20.99 |
| A05 | BAG2 | BAG family molecular chaperone regulator 2-like | 29.88 | 29.37 | 30.68 |
| A06 | BNIP3 | BCL2/adenovirus E1B 19 kDa protein-interacting protein 3-like | 29.99 | 29.71 | 30.73 |
| A07 | CAT | Catalase | 21.14 | 20.93 | 21.34 |
| A08 | CCL5 | Chemokine (C-C motif) ligand 5 | 28.38 | 28.49 | 29.37 |
| A09 | CYGB | Cytoglobin-like | 28.57 | 29.75 | 30.28 |
| A10 | DHCR24 | 24-dehydrocholesterol reductase | 25.1 | 25.28 | 25.88 |
| A11 | DUOX1 | Dual oxidase 1 | 36.79 | 36.91 | 38.64 |
| A12 | DUOX2 | Dual oxidase 2 | 34.85 | 36.83 | 38.35 |
| B01 | DUSP1 | Dual specificity phosphatase 1 | 27.02 | 26.97 | 27.8 |
| B02 | EPHX2 | Epoxide hydrolase 2, cytoplasmic | 24.79 | 24.3 | 24.53 |
| B03 | EPX | Eosinophil peroxidase | 38.31 | 37.35 | 38.7 |
| B04 | FHL2 | Four and a half LIM domains protein 2-like | 31.01 | 31.36 | 34.1 |
| B05 | FOXM1 | Forkhead box M1 | 31.75 | 33.88 | 33.43 |
| B06 | FTH1 | Ferritin, heavy polypeptide 1 | 18.82 | 18.36 | 19.11 |
| B07 | GCLC | Glutamate-cysteine ligase, catalytic subunit | 24.81 | 24.66 | 25.43 |
| B08 | GCLM | Glutamate-cysteine ligase, modifier subunit | 28.8 | 28.74 | 27.93 |
| B09 | GLA | Alpha-galactosidase A | 27.21 | 28.1 | 28.17 |
| B10 | GP91-PHOX | NADPH oxidase heavy chain subunit | 28.4 | 29.43 | 31 |
| B11 | GPX1 | Glutathione peroxidase 1 | 23.1 | 23.01 | 22.63 |
| B12 | GPX2 | Glutathione peroxidase 2 (gastrointestinal) | 34.14 | 34.79 | 37.83 |
| C01 | GPX3 | Glutathione peroxidase 3 (plasma) | 26.11 | 26.82 | 28.13 |
| C02 | GPX4 | Glutathione peroxidase 4 (phospholipid hydroperoxidase) | 23.58 | 23.03 | 23.78 |
| C03 | GPX5 | Glutathione peroxidase 5 (epididymal androgen-related protein) | 39.03 | 39.9 | 40 |
| C04 | GSS | Glutathione synthetase | 26.51 | 27.19 | 28.29 |
| C05 | GSTZ1 | Glutathione S-transferase zeta 1 | 26.17 | 26.89 | 27.58 |
| C06 | HMOX1 | Heme oxygenase (decycling) 1 | 26.29 | 26.63 | 27.88 |
| C07 | HSP70.2 | Heat shock protein 70.2 | 25.67 | 25.63 | 27.5 |
| C08 | HSP90AA1 | 90-kDa heat shock protein | 24 | 23.45 | 24.49 |
| C09 | KRT1 | Keratin 1 | 40 | 38.38 | 40 |
| C10 | LHPP | Phospholysine phosphohistidine inorganic pyrophosphate phosphatase | 28.09 | 28.91 | 29.86 |
| C11 | LOC100049683 | Solute carrier family 7 (anionic amino acid transporter light chain, xc- system), member 11 | 30.51 | 30.32 | 30.36 |
| C12 | LOC100516395 | Lactoperoxidase-like | 32.15 | 33.91 | 31.06 |
| D01 | TTN | Titin-like | 32.33 | 32.95 | 35.45 |
| D02 | LOC100622575 | Pancreatic secretory trypsin inhibitor-like | 34.77 | 36.33 | 40 |
| D03 | LOC100739163 | Glutathione S-transferase P | 26.64 | 25.57 | 25.71 |
| D04 | LOC396625 | Glutathione reductase | 26.99 | 26.59 | 27.35 |
| D05 | LOC733635 | Aldo-keto reductase family 1 member C2-like | 26.43 | 27 | 28.66 |
| D06 | MB | Myoglobin | 40 | 40 | 40 |
| D07 | MBL2 | Mannose-binding lectin (protein C) 2, soluble | 24.29 | 24.73 | 24.25 |
| D08 | MGST3 | Microsomal glutathione S-transferase 3 | 22.84 | 22.75 | 22.47 |
| D09 | MPO | Myeloperoxidase | 34.03 | 35.51 | 35.29 |
| D10 | MPV17 | MpV17 mitochondrial inner membrane protein | 34.58 | 34.87 | 36.07 |
| D11 | MSRA | Peptide methionine sulfoxide reductase | 25.56 | 27.09 | 26.95 |
| D12 | MT-III | Metallothionein-III | 24.51 | 24.69 | 26.91 |
| E01 | NCF1 | Neutrophil cytosolic factor 1 | 29.9 | 30.82 | 32.56 |
| E02 | NCF2 | Neutrophil cytosolic factor 2 | 30.1 | 30.67 | 32.4 |
| E03 | NCOA7 | Nuclear receptor coactivator 7 | 29.7 | 29.77 | 31.16 |
| E04 | NOS2 | Nitric oxide synthase 2, inducible | 34.77 | 35.6 | 36 |
| E05 | NOX4 | NADPH oxidase 4 | 31.65 | 35.07 | 37.42 |
| E06 | NOX5 | NADPH oxidase, EF-hand calcium binding domain 5 | 36.55 | 34.9 | 37.87 |
| E07 | NQO1 | NAD(P)H dehydrogenase, quinone 1 | 25.26 | 24.4 | 23.62 |
| E08 | NUDT1 | Nudix (nucleoside diphosphate linked moiety X)-type motif 1 | 27.96 | 28.54 | 28.16 |
| E09 | OXR1 | Oxidation resistance 1 | 26.83 | 26.62 | 27.38 |
| E10 | OXSR1 | Oxidative-stress responsive 1 | 28.24 | 28.87 | 29.99 |
| E11 | PDLIM1 | PDZ and LIM domain 1 | 25.72 | 25.27 | 26.01 |
| E12 | PRDX1 | Peroxiredoxin-1-like | 23.27 | 22.48 | 22.29 |
| F01 | PRDX2 | Peroxiredoxin 2 | 24.22 | 23.46 | 24.01 |
| F02 | PRDX3 | Peroxiredoxin 3 | 22.68 | 21.64 | 21.69 |
| F03 | PRDX4 | Peroxiredoxin 4 | 21.87 | 21.24 | 21.83 |
| F04 | PRDX5 | Peroxiredoxin 5 | 24.39 | 23.57 | 23.83 |
| F05 | PRDX6 | Peroxiredoxin 6 | 22.99 | 21.85 | 22.05 |
| F06 | PRNP | Prion protein | 30.79 | 31.01 | 31.6 |
| F07 | PTGR1 | Prostaglandin reductase 1 | 24.92 | 23.95 | 22.89 |
| F08 | PTGS1 | Prostaglandin-endoperoxide synthase 1 (prostaglandin G/H synthase and cyclooxygenase) | 28.93 | 29.64 | 30.58 |
| F09 | PTGS2 | Prostaglandin G/H synthase-2 | 31.93 | 32.19 | 33.11 |
| F10 | RNF7 | Ring finger protein 7 | 26.47 | 25.93 | 26.39 |
| F11 | SCARA3 | Scavenger receptor class A, member 3 | 31.69 | 33.11 | 35.33 |
| F12 | SEPP1 | Selenoprotein P, plasma, 1 | 20.13 | 18.62 | 18.86 |
| G01 | SFTPD | Surfactant protein D | 34.48 | 34.88 | 37.47 |
| G02 | SIRT2 | Sirtuin 2 | 29.18 | 29.42 | 31.03 |
| G03 | SOD1 | Superoxide dismutase 1, soluble | 22.74 | 21.77 | 22.75 |
| G04 | SOD3 | Extracellular superoxide dismutase | 28.91 | 28.69 | 29.9 |
| G05 | SQSTM1 | Sequestosome-1-like | 26.6 | 26.07 | 27.05 |
| G06 | TRAPPC6B | Trafficking protein particle complex subunit 6B-like | 30.37 | 29.68 | 30.32 |
| G07 | TTNLOC100620261 | Titin | 33.24 | 34.44 | 38.3 |
| G08 | TXN | Thioredoxin | 23.11 | 21.79 | 21.45 |
| G09 | TXNRD1 | Thioredoxin reductase 1 | 27.11 | 25.84 | 26.62 |
| G10 | TXNRD2 | Thioredoxin reductase 2 | 27.98 | 27.78 | 28.85 |
| G11 | UCP2 | Uncoupling protein 2 (mitochondrial, proton carrier) | 34.3 | 34.46 | 36.06 |
| G12 | VIMP | Selenoprotein S | 25.16 | 23.94 | 24.26 |
| H01 | ACTG1 | Actin gamma 1 | 22.16 | 21.81 | 23.23 |
| H02 | B2M | Beta-2-microglobulin | 21.63 | 20.35 | 20.99 |
| H03 | GAPDH | Glyceraldehyde-3-phosphate dehydrogenase | 23.38 | 23.19 | 23.72 |
| H04 | HPRT1 | Hypoxanthine phosphoribosyltransferase 1 | 24.14 | 22.53 | 22.7 |
| H05 | RPL13A | Ribosomal protein L13a | 21 | 19.58 | 20.33 |
| H06 | SGDC | Pig Genomic DNA Contamination | 40 | 40 | 40 |
| H07 | RTC | Reverse Transcription Control | 22.76 | 22.46 | 22.83 |
| H08 | RTC | Reverse Transcription Control | 23.11 | 22.68 | 22.78 |
| H09 | RTC | Reverse Transcription Control | 23.4 | 22.36 | 23.15 |
| H10 | PPC | Positive PCR Control | 21.01 | 20.44 | 20.58 |
| H11 | PPC | Positive PCR Control | 21.06 | 20.45 | 20.64 |
| H12 | PPC | Positive PCR Control | 21.16 | 20.61 | 20.8 |

Normalization analysis used reference genes, ACTG1 (Actin gamma 1), GAPDH (Glyceraldehyde-3-phosphate dehydrogenase), HPRT1 (Hypoxanthine phosphoribosyltransferase 1), RPL13A (Ribosomal protein L13a), n = 3 piglets.

**S10 Table. RT² Profiler™ PCR Array Pig Mitochondrial Energy Metabolism CT values in the liver of 14, 23 and 35 days old normal birth weight piglets**

| Well | Symbol | Description | D14 | D23 | D35 |
| --- | --- | --- | --- | --- | --- |
| A01 | ARRDC3 | Arrestin domain containing 3 | 23.77 | 23.72 | 24.39 |
| A02 | ASB1 | Ankyrin repeat and SOCS box protein 1-like | 28.51 | 30.07 | 30.68 |
| A03 | ATP12A | ATPase, H+/K+ transporting, nongastric, alpha polypeptide | 39.74 | 40 | 40 |
| A04 | ATP4A | ATPase, H+/K+ exchanging, alpha polypeptide | 33.57 | 35.42 | 37.22 |
| A05 | ATP4B | ATPase, H+/K+ exchanging, beta polypeptide | 32.56 | 36.27 | 33.82 |
| A06 | ATP5A1 | ATP synthase, H+ transporting, mitochondrial F1 complex, alpha subunit 1, cardiac muscle | 22.31 | 23.59 | 23.15 |
| A07 | ATP5B | ATP synthase, H+ transporting, mitochondrial F1 complex, beta polypeptide | 20.45 | 20.92 | 21.13 |
| A08 | ATP5E | ATP synthase, H+ transporting, mitochondrial F1 complex, epsilon subunit | 20.86 | 21.3 | 20.93 |
| A09 | ATP5F1 | ATP synthase, H+ transporting, mitochondrial Fo complex, subunit B1 | 24.49 | 24.38 | 25.17 |
| A10 | ATP5G1 | ATP synthase, H+ transporting, mitochondrial Fo complex, subunit C1 (subunit 9) | 22.39 | 23.47 | 23.24 |
| A11 | LOC100037988 | ATP synthase, H+ transporting, mitochondrial Fo complex, subunit C2 (subunit 9) | 39.22 | 39.81 | 39.6 |
| A12 | ATP5G3 | ATP synthase, H+ transporting, mitochondrial Fo complex, subunit C3 (subunit 9) | 26.92 | 21.01 | 20.83 |
| B01 | ATP5H | ATP synthase, H+ transporting, mitochondrial Fo complex, subunit d | 21.56 | 21.48 | 21.91 |
| B02 | ATP5I | ATP synthase, H+ transporting, mitochondrial Fo complex, subunit E | 22.13 | 22.29 | 22.46 |
| B03 | ATP5L | ATP synthase, H+ transporting, mitochondrial Fo complex, subunit G | 27.32 | 27.32 | 27.39 |
| B04 | ATP6V0A2 | ATPase, H+ transporting, lysosomal V0 subunit a2 | 27.61 | 28.71 | 28.9 |
| B05 | ATP6V0D2 | ATPase, H+ transporting, lysosomal 38kDa, V0 subunit d2 | 29.47 | 32.44 | 30.09 |
| B06 | ATP6V1C2 | ATPase, H+ transporting, lysosomal 42kDa, V1 subunit C2 | 24.18 | 25.52 | 26.09 |
| B07 | ATP6V1E2 | ATPase, H+ transporting, lysosomal 31kDa, V1 subunit E2 | 34.49 | 35.42 | 38.18 |
| B08 | ATP6V1G3 | V-type proton ATPase subunit G 3-like | 26.79 | 28.45 | 28.44 |
| B09 | BCS1L | BCS1-like (S. cerevisiae) | 28.46 | 29.71 | 30.84 |
| B10 | Cox5b | Mitochondrial cytochrome c oxidase subunit Vb | 21.57 | 22.5 | 22.1 |
| B11 | COX6A1 | Cytochrome c oxidase subunit VIa polypeptide 1 | 22.27 | 22.02 | 22.43 |
| B12 | COX6C | COX6C protein | 21.75 | 21.61 | 21.82 |
| C01 | COX7A2 | COX7A2 protein | 22.72 | 22.74 | 23.06 |
| C02 | DNAJB1 | DnaJ (Hsp40) homolog, subfamily B, member 1 | 25.42 | 25.87 | 27.07 |
| C03 | EDN1 | Endothelin 1 | 30.72 | 31.9 | 32.58 |
| C04 | GADD45B | Growth arrest and DNA-damage-inducible, beta | 29.15 | 30.16 | 30.71 |
| C05 | HSP70.2 | Heat shock protein 70.2 | 24.82 | 26.12 | 27.6 |
| C06 | LHPP | Phospholysine phosphohistidine inorganic pyrophosphate phosphatase | 27.08 | 29.3 | 29.88 |
| C07 | LOC100154992 | Mitochondrial inner membrane protein OXA1L-like | 24.72 | 25.48 | 26.33 |
| C08 | LOC100156375 | COX15 homolog, cytochrome c oxidase assembly protein | 26.52 | 28.26 | 27.48 |
| C09 | LOC100156879 | Ubiquinol--cytochrome c reductase | 24.25 | 25.11 | 25.67 |
| C10 | LOC100156967 | Cytochrome c oxidase subunit 5A, mitochondrial-like | 21.91 | 23.08 | 22.34 |
| C11 | LOC100157935 | Cytochrome c oxidase subunit 8C, mitochondrial-like | 35.17 | 37.2 | 37.92 |
| C12 | LOC100511690 | Cytochrome c1, heme protein, mitochondrial-like | 22.91 | 24.01 | 24.24 |
| D01 | LOC100517408 | Cytochrome c oxidase subunit 7B2, mitochondrial-like | 36.05 | 34.83 | 40 |
| D02 | LOC100519366 | Cytochrome c oxidase subunit 6B1-like | 32.64 | 31.89 | 31.78 |
| D03 | LOC100519594 | Cytochrome c oxidase subunit 6A2, mitochondrial-like | 28.84 | 30.05 | 30.65 |
| D04 | LOC100522725 | Cytochrome c oxidase subunit 8A, mitochondrial-like | 24.18 | 25.35 | 25.65 |
| D05 | LOC100624067 | Cytochrome c oxidase subunit 4 isoform 1, mitochondrial-like | 21.83 | 22.65 | 22.53 |
| D06 | LOC100624950 | Cytochrome b561 domain-containing protein 1-like | 28.27 | 28.51 | 29.29 |
| D07 | LOC100739238 | Low-density lipoprotein receptor-related protein 5-like | 25.4 | 26.92 | 28.35 |
| D08 | NDUFB6 | NADH dehydrogenase 1 beta subcomplex 6 | 23.88 | 25.09 | 24.42 |
| D09 | ATP5O | ATP synthase H+-transporting mitochondrial F1 complex O subunit | 22.15 | 21.9 | 22.21 |
| D10 | NDUFA1 | NADH dehydrogenase [ubiquinone] 1 alpha subcomplex subunit 1-like | 24.17 | 25.23 | 24.93 |
| D11 | NDUFA10 | NADH dehydrogenase [ubiquinone] 1 alpha subcomplex subunit 10, mitochondrial-like | 25.29 | 25.49 | 26.51 |
| D12 | NDUFA11 | NADH dehydrogenase (ubiquinone) 1 alpha subcomplex, 11, 14.7kDa | 24.18 | 24.76 | 25.16 |
| E01 | NDUFA2 | NADH dehydrogenase (ubiquinone) 1 alpha subcomplex, 2, 8kDa | 28.1 | 30.7 | 27.36 |
| E02 | NDUFA3 | NADH dehydrogenase [ubiquinone] 1 alpha subcomplex subunit 3-like | 24.86 | 25.82 | 26.15 |
| E03 | NDUFA4 | NADH dehydrogenase (ubiquinone) 1 alpha subcomplex, 4, 9kDa | 20.17 | 20.34 | 20.57 |
| E04 | NDUFA5 | NADH dehydrogenase [ubiquinone] 1 alpha subcomplex subunit 5-like | 23.31 | 23.84 | 23.47 |
| E05 | NDUFA6 | NADH dehydrogenase (ubiquinone) 1 alpha subcomplex, 6, 14kDa | 29.75 | 31.64 | 32.81 |
| E06 | NDUFA7 | NADH dehydrogenase [ubiquinone] 1 alpha subcomplex subunit 7-like | 24.36 | 24.81 | 24.48 |
| E07 | NDUFA8 | NADH dehydrogenase (ubiquinone) 1 alpha subcomplex, 8, 19kDa | 23.74 | 24.3 | 24.34 |
| E08 | NDUFA9 | NADH dehydrogenase [ubiquinone] 1 alpha subcomplex subunit 9, mitochondrial-like | 38.32 | 40 | 40 |
| E09 | NDUFAB1 | NADH dehydrogenase (ubiquinone) 1, alpha/beta subcomplex, 1, 8kDa | 24.08 | 24.45 | 24.83 |
| E10 | NDUFB10 | NADH dehydrogenase [ubiquinone] 1 beta subcomplex subunit 10-like | 23.29 | 23.4 | 23.8 |
| E11 | NDUFB2 | NADH dehydrogenase (ubiquinone) 1 beta subcomplex, 2, 8kDa | 22.39 | 22.64 | 22.6 |
| E12 | NDUFB3 | NADH dehydrogenase (ubiquinone) 1 beta subcomplex, 3, 12kDa | 32.15 | 32.47 | 32.09 |
| F01 | NDUFB4 | NADH dehydrogenase [ubiquinone] 1 beta subcomplex subunit 4-like | 23.94 | 24.42 | 24.72 |
| F02 | NDUFB5 | NADH dehydrogenase (ubiquinone) 1 beta subcomplex, 5, 16kDa | 28.33 | 34.23 | 28.59 |
| F03 | NDUFB7 | NADH dehydrogenase (ubiquinone) 1 beta subcomplex, 7, 18kDa | 33.43 | 36 | 40 |
| F04 | NDUFB8 | NADH dehydrogenase (ubiquinone) 1 beta subcomplex, 8, 19kDa | 23.95 | 24.28 | 23.87 |
| F05 | NDUFB9 | NADH dehydrogenase (ubiquinone) 1 beta subcomplex, 9, 22kDa | 22.61 | 22.62 | 22.71 |
| F06 | NDUFC1 | NADH dehydrogenase (ubiquinone) 1, subcomplex unknown, 1, 6kDa | 24.12 | 24.52 | 24.11 |
| F07 | NDUFS1 | NADH dehydrogenase (ubiquinone) Fe-S protein 1, 75kDa (NADH-coenzyme Q reductase) | 25.75 | 27.05 | 27.06 |
| F08 | NDUFS2 | NADH dehydrogenase (ubiquinone) Fe-S protein 2, 49kDa (NADH-coenzyme Q reductase) | 31.61 | 34.15 | 35.77 |
| F09 | NDUFS3 | NADH dehydrogenase (ubiquinone) Fe-S protein 3, 30kDa (NADH-coenzyme Q reductase) | 23.59 | 24.28 | 24.87 |
| F10 | NDUFS4 | NADH dehydrogenase (ubiquinone) Fe-S protein 4, 18kDa (NADH-coenzyme Q reductase) | 22.8 | 22.59 | 22.78 |
| F11 | NDUFS5 | NADH dehydrogenase (ubiquinone) Fe-S protein 5, 15kDa (NADH-coenzyme Q reductase) | 21.82 | 22.41 | 22.34 |
| F12 | NDUFS6 | NADH dehydrogenase [ubiquinone] iron-sulfur protein 6, mitochondrial-like | 23.13 | 24.02 | 24.26 |
| G01 | NDUFS7 | NADH dehydrogenase [ubiquinone] iron-sulfur protein 7, mitochondrial-like | 23.04 | 23.32 | 24.16 |
| G02 | NDUFS8 | NADH dehydrogenase [ubiquinone] iron-sulfur protein 8, mitochondrial-like | 31.88 | 33.39 | 33.19 |
| G03 | NDUFV1 | NADH dehydrogenase [ubiquinone] flavoprotein 1, mitochondrial-like | 29.94 | 31.79 | 32.41 |
| G04 | PPA1 | Pyrophosphatase 1 | 22.56 | 23.07 | 22.28 |
| G05 | PPA2 | Inorganic pyrophosphatase 2, mitochondrial-like | 24.55 | 24.79 | 32.13 |
| G06 | SDHA | Succinate dehydrogenase [ubiquinone] flavoprotein subunit, mitochondrial | 22.23 | 23.68 | 23.75 |
| G07 | SDHB | Succinate dehydrogenase complex, subunit B, iron sulfur (Ip) | 21.41 | 22.93 | 23.07 |
| G08 | SDHC | Succinate dehydrogenase cytochrome b560 subunit, mitochondrial-like | 23.39 | 23.6 | 23.74 |
| G09 | SDHD | Succinate dehydrogenase complex, subunit D, integral membrane protein | 22.93 | 23.1 | 23.47 |
| G10 | SLC25A25 | Solute carrier family 25 (mitochondrial carrier; phosphate carrier), member 25 | 28.75 | 30.34 | 31.75 |
| G11 | UCP2 | Uncoupling protein 2 (mitochondrial, proton carrier) | 31.62 | 34.35 | 35.77 |
| G12 | UQCR10 | Ubiquinol-cytochrome c reductase complex 7.2 kDa protein | 29.12 | 24.34 | 24.29 |
| H01 | ACTG1 | Actin gamma 1 | 21.13 | 22.14 | 23.47 |
| H02 | B2M | Beta-2-microglobulin | 20.48 | 20.55 | 21.01 |
| H03 | GAPDH | Glyceraldehyde-3-phosphate dehydrogenase | 22.38 | 24.06 | 24.18 |
| H04 | HPRT1 | Hypoxanthine phosphoribosyltransferase 1 | 23.2 | 23.4 | 23.04 |
| H05 | RPL13A | Ribosomal protein L13a | 20.48 | 19.82 | 20.39 |
| H06 | SGDC | Pig Genomic DNA Contamination | 40 | 40 | 40 |
| H07 | RTC | Reverse Transcription Control | 22.12 | 22.51 | 22.76 |
| H08 | RTC | Reverse Transcription Control | 22.28 | 22.22 | 22.67 |
| H09 | RTC | Reverse Transcription Control | 23.03 | 22.07 | 22.76 |
| H10 | PPC | Positive PCR Control | 20.18 | 20.54 | 20.37 |
| H11 | PPC | Positive PCR Control | 20.27 | 20.4 | 20.42 |
| H12 | PPC | Positive PCR Control | 20.38 | 20.59 | 20.68 |

Normalization analysis used reference genes, B2M (Beta-2-microglobulin), HPRT1 (Hypoxanthine phosphoribosyltransferase 1), RPL13A (Ribosomal protein L13a).

**S11Table. RT² Profiler™ PCR Array Pig Oxidative Stress CT values in the intestinal mucosa of 14, 23 and 35 days old normal birth weight piglets**

| Well | Symbol | Description | D14 | D23 | D35 |
| --- | --- | --- | --- | --- | --- |
| A01 | ALB | Albumin | 33.29 | 35.33 | 31.22 |
| A02 | ALOX12 | Arachidonate 12-lipoxygenase | 30.66 | 29.25 | 31.69 |
| A03 | AOX1 | Aldehyde oxidase-like | 30.28 | 30.21 | 31.47 |
| A04 | APOE | Apolipoprotein E | 28.78 | 26.89 | 28.85 |
| A05 | BAG2 | BAG family molecular chaperone regulator 2-like | 29.57 | 28.07 | 28.92 |
| A06 | BNIP3 | BCL2/adenovirus E1B 19 kDa protein-interacting protein 3-like | 29.07 | 28.56 | 29.59 |
| A07 | CAT | Catalase | 23.88 | 23.37 | 24.13 |
| A08 | CCL5 | Chemokine (C-C motif) ligand 5 | 24.86 | 23.96 | 23.87 |
| A09 | CYGB | Cytoglobin-like | 27.11 | 26.48 | 28.07 |
| A10 | DHCR24 | 24-dehydrocholesterol reductase | 27.23 | 26.26 | 27.23 |
| A11 | DUOX1 | Dual oxidase 1 | 35.38 | 34.15 | 35.02 |
| A12 | DUOX2 | Dual oxidase 2 | 35.26 | 31.25 | 32.32 |
| B01 | DUSP1 | Dual specificity phosphatase 1 | 25.53 | 25.19 | 26.72 |
| B02 | EPHX2 | Epoxide hydrolase 2, cytoplasmic | 27.1 | 27.14 | 26.32 |
| B03 | EPX | Eosinophil peroxidase | 39.18 | 33.56 | 39.01 |
| B04 | FHL2 | Four and a half LIM domains protein 2-like | 29.73 | 28.69 | 30.69 |
| B05 | FOXM1 | Forkhead box M1 | 30.63 | 28.86 | 30.65 |
| B06 | FTH1 | Ferritin, heavy polypeptide 1 | 17.4 | 17.22 | 18.79 |
| B07 | GCLC | Glutamate-cysteine ligase, catalytic subunit | 25.36 | 24.4 | 25.91 |
| B08 | GCLM | Glutamate-cysteine ligase, modifier subunit | 28.82 | 28.79 | 27.92 |
| B09 | GLA | Alpha-galactosidase A | 24.58 | 25.79 | 26.1 |
| B10 | GP91-PHOX | NADPH oxidase heavy chain subunit | 28.36 | 25.72 | 28.84 |
| B11 | GPX1 | Glutathione peroxidase 1 | 24.85 | 23.45 | 24.84 |
| B12 | GPX2 | Glutathione peroxidase 2 (gastrointestinal) | 28.38 | 26.46 | 26.19 |
| C01 | GPX3 | Glutathione peroxidase 3 (plasma) | 23.98 | 25.87 | 28.5 |
| C02 | GPX4 | Glutathione peroxidase 4 (phospholipid hydroperoxidase) | 22.01 | 22.88 | 23.21 |
| C03 | GPX5 | Glutathione peroxidase 5 (epididymal androgen-related protein) | 38.64 | 37.18 | 38.17 |
| C04 | GSS | Glutathione synthetase | 26.78 | 26.39 | 27.62 |
| C05 | GSTZ1 | Glutathione S-transferase zeta 1 | 29.01 | 29.02 | 31.01 |
| C06 | HMOX1 | Heme oxygenase (decycling) 1 | 26.12 | 26.56 | 28.6 |
| C07 | HSP70.2 | Heat shock protein 70.2 | 25.22 | 23.33 | 24.48 |
| C08 | HSP90AA1 | 90-kDa heat shock protein | 22.42 | 21.4 | 23.03 |
| C09 | KRT1 | Keratin 1 | 39.59 | 38.87 | 40 |
| C10 | LHPP | Phospholysine phosphohistidine inorganic pyrophosphate phosphatase | 28.92 | 27.15 | 28.7 |
| C11 | LOC100049683 | Solute carrier family 7 (anionic amino acid transporter light chain, xc- system), member 11 | 33.01 | 31.43 | 30.57 |
| C12 | LOC100516395 | Lactoperoxidase-like | 40 | 38.73 | 37.8 |
| D01 | TTN | Titin-like | 33.48 | 32.07 | 33.88 |
| D02 | LOC100622575 | Pancreatic secretory trypsin inhibitor-like | 16.37 | 16.24 | 15.71 |
| D03 | LOC100739163 | Glutathione S-transferase P | 22.46 | 21.21 | 22 |
| D04 | LOC396625 | Glutathione reductase | 25.39 | 25.02 | 24.72 |
| D05 | LOC733635 | Aldo-keto reductase family 1 member C2-like | 40 | 40 | 40 |
| D06 | MB | Myoglobin | 39.21 | 39.8 | 40 |
| D07 | MBL2 | Mannose-binding lectin (protein C) 2, soluble | 39.45 | 37.88 | 38.79 |
| D08 | MGST3 | Microsomal glutathione S-transferase 3 | 21.45 | 22.02 | 21.04 |
| D09 | MPO | Myeloperoxidase | 40 | 35.3 | 37.97 |
| D10 | MPV17 | MpV17 mitochondrial inner membrane protein | 34.09 | 32.66 | 33.27 |
| D11 | MSRA | Peptide methionine sulfoxide reductase | 25.76 | 26.18 | 25.88 |
| D12 | MT-III | Metallothionein-III | 26.69 | 21.71 | 26.57 |
| E01 | NCF1 | Neutrophil cytosolic factor 1 | 29.4 | 26.99 | 29.9 |
| E02 | NCF2 | Neutrophil cytosolic factor 2 | 30.06 | 28.34 | 29.86 |
| E03 | NCOA7 | Nuclear receptor coactivator 7 | 27.9 | 27.16 | 28.63 |
| E04 | NOS2 | Nitric oxide synthase 2, inducible | 31.78 | 28.78 | 28.89 |
| E05 | NOX4 | NADPH oxidase 4 | 28.61 | 27.83 | 30.61 |
| E06 | NOX5 | NADPH oxidase, EF-hand calcium binding domain 5 | 33.73 | 33.52 | 35.32 |
| E07 | NQO1 | NAD(P)H dehydrogenase, quinone 1 | 25.93 | 24.01 | 25.3 |
| E08 | NUDT1 | Nudix (nucleoside diphosphate linked moiety X)-type motif 1 | 27.08 | 26.72 | 26.92 |
| E09 | OXR1 | Oxidation resistance 1 | 26.07 | 26.47 | 26.69 |
| E10 | OXSR1 | Oxidative-stress responsive 1 | 27.09 | 26.69 | 27.96 |
| E11 | PDLIM1 | PDZ and LIM domain 1 | 24.98 | 24.18 | 25.38 |
| E12 | PRDX1 | Peroxiredoxin-1-like | 20.04 | 20.34 | 20 |
| F01 | PRDX2 | Peroxiredoxin 2 | 22.18 | 22.51 | 22.24 |
| F02 | PRDX3 | Peroxiredoxin 3 | 22.07 | 22.81 | 22.09 |
| F03 | PRDX4 | Peroxiredoxin 4 | 22.85 | 23.15 | 22.53 |
| F04 | PRDX5 | Peroxiredoxin 5 | 23.11 | 23.09 | 23.45 |
| F05 | PRDX6 | Peroxiredoxin 6 | 22.04 | 22.78 | 22.66 |
| F06 | PRNP | Prion protein | 29.83 | 29.21 | 30.31 |
| F07 | PTGR1 | Prostaglandin reductase 1 | 25.32 | 24.08 | 24.23 |
| F08 | PTGS1 | Prostaglandin-endoperoxide synthase 1 (prostaglandin G/H synthase and cyclooxygenase) | 29.65 | 28.14 | 30.7 |
| F09 | PTGS2 | Prostaglandin G/H synthase-2 | 31.38 | 29.17 | 31.67 |
| F10 | RNF7 | Ring finger protein 7 | 24.04 | 24.57 | 24.73 |
| F11 | SCARA3 | Scavenger receptor class A, member 3 | 32.02 | 30.38 | 32.39 |
| F12 | SEPP1 | Selenoprotein P, plasma, 1 | 17.01 | 18.64 | 17.82 |
| G01 | SFTPD | Surfactant protein D | 26.95 | 25.98 | 25.66 |
| G02 | SIRT2 | Sirtuin 2 | 28.39 | 28.06 | 29.59 |
| G03 | SOD1 | Superoxide dismutase 1, soluble | 23.48 | 23.38 | 24.08 |
| G04 | SOD3 | Extracellular superoxide dismutase | 28.58 | 25.94 | 28.76 |
| G05 | SQSTM1 | Sequestosome-1-like | 25.9 | 25.06 | 26.5 |
| G06 | TRAPPC6B | Trafficking protein particle complex subunit 6B-like | 28.78 | 28.63 | 28.95 |
| G07 | TTNLOC100620261 | Titin | 38.81 | 39.06 | 40 |
| G08 | TXN | Thioredoxin | 18.65 | 18.4 | 18.39 |
| G09 | TXNRD1 | Thioredoxin reductase 1 | 23.69 | 22.49 | 23.49 |
| G10 | TXNRD2 | Thioredoxin reductase 2 | 28.03 | 26.75 | 28.23 |
| G11 | UCP2 | Uncoupling protein 2 (mitochondrial, proton carrier) | 32.4 | 30.77 | 33.86 |
| G12 | VIMP | Selenoprotein S | 23 | 23.41 | 23.17 |
| H01 | ACTG1 | Actin gamma 1 | 19.36 | 18.4 | 20.15 |
| H02 | B2M | Beta-2-microglobulin | 17.95 | 18.18 | 17.64 |
| H03 | GAPDH | Glyceraldehyde-3-phosphate dehydrogenase | 21.69 | 21.07 | 22.31 |
| H04 | HPRT1 | Hypoxanthine phosphoribosyltransferase 1 | 25.47 | 25.24 | 25.67 |
| H05 | RPL13A | Ribosomal protein L13a | 18.57 | 18.77 | 18.5 |
| H06 | SGDC | Pig Genomic DNA Contamination | 40 | 40 | 40 |
| H07 | RTC | Reverse Transcription Control | 22.4 | 22.23 | 22.51 |
| H08 | RTC | Reverse Transcription Control | 21.68 | 22.28 | 22.54 |
| H09 | RTC | Reverse Transcription Control | 21.83 | 22.29 | 22.6 |
| H10 | PPC | Positive PCR Control | 20.47 | 20.52 | 20.59 |
| H11 | PPC | Positive PCR Control | 20.48 | 20.58 | 20.72 |
| H12 | PPC | Positive PCR Control | 20.63 | 20.71 | 20.82 |

Normalization analysis used reference genes, B2M (Beta-2-microglobulin), GAPDH (Glyceraldehyde-3-phosphate dehydrogenase), HPRT1 (Hypoxanthine phosphoribosyltransferase 1), RPL13A (Ribosomal protein L13a), n = 3 piglets.

**S12 Table. RT² Profiler™ PCR Array Pig Mitochondrial Energy Metabolism average CT values in the intestinal mucosa of 14, 23 and 35 days old normal birth weight piglets**

| Well | Symbol | Description | D14 | D23 | D35 |
| --- | --- | --- | --- | --- | --- |
| A01 | ARRDC3 | Arrestin domain containing 3 | 24.61 | 24.93 | 25.48 |
| A02 | ASB1 | Ankyrin repeat and SOCS box protein 1-like | 29.68 | 28.08 | 30.53 |
| A03 | ATP12A | ATPase, H+/K+ transporting, nongastric, alpha polypeptide | 35.53 | 32.06 | 35.56 |
| A04 | ATP4A | ATPase, H+/K+ exchanging, alpha polypeptide | 37.15 | 34.28 | 36.75 |
| A05 | ATP4B | ATPase, H+/K+ exchanging, beta polypeptide | 34.46 | 32.7 | 30.72 |
| A06 | ATP5A1 | ATP synthase, H+ transporting, mitochondrial F1 complex, alpha subunit 1, cardiac muscle | 22.01 | 21.88 | 21.46 |
| A07 | ATP5B | ATP synthase, H+ transporting, mitochondrial F1 complex, beta polypeptide | 20.37 | 19.99 | 19.43 |
| A08 | ATP5E | ATP synthase, H+ transporting, mitochondrial F1 complex, epsilon subunit | 20.48 | 20.88 | 19.69 |
| A09 | ATP5F1 | ATP synthase, H+ transporting, mitochondrial Fo complex, subunit B1 | 24.2 | 24.15 | 22.97 |
| A10 | ATP5G1 | ATP synthase, H+ transporting, mitochondrial Fo complex, subunit C1 (subunit 9) | 21.56 | 21.14 | 20.56 |
| A11 | LOC100037988 | ATP synthase, H+ transporting, mitochondrial Fo complex, subunit C2 (subunit 9) | 38.34 | 36.56 | 39.51 |
| A12 | ATP5G3 | ATP synthase, H+ transporting, mitochondrial Fo complex, subunit C3 (subunit 9) | 19.74 | 20.28 | 19.14 |
| B01 | ATP5H | ATP synthase, H+ transporting, mitochondrial Fo complex, subunit d | 20.45 | 21.05 | 20.13 |
| B02 | ATP5I | ATP synthase, H+ transporting, mitochondrial Fo complex, subunit E | 21 | 21.61 | 21.13 |
| B03 | ATP5L | ATP synthase, H+ transporting, mitochondrial Fo complex, subunit G | 26.69 | 26.65 | 26.76 |
| B04 | ATP6V0A2 | ATPase, H+ transporting, lysosomal V0 subunit a2 | 27.45 | 26.54 | 27.27 |
| B05 | ATP6V0D2 | ATPase, H+ transporting, lysosomal 38kDa, V0 subunit d2 | 39.84 | 34.86 | 40 |
| B06 | ATP6V1C2 | ATPase, H+ transporting, lysosomal 42kDa, V1 subunit C2 | 26.03 | 25.45 | 26.32 |
| B07 | ATP6V1E2 | ATPase, H+ transporting, lysosomal 31kDa, V1 subunit E2 | 37.03 | 33.45 | 36.22 |
| B08 | ATP6V1G3 | V-type proton ATPase subunit G 3-like | 32.8 | 31.2 | 33.09 |
| B09 | BCS1L | BCS1-like (S. cerevisiae) | 31.04 | 28.21 | 29.19 |
| B10 | Cox5b | Mitochondrial cytochrome c oxidase subunit Vb | 20.9 | 20.78 | 20.01 |
| B11 | COX6A1 | Cytochrome c oxidase subunit VIa polypeptide 1 | 21.07 | 21.31 | 20.3 |
| B12 | COX6C | COX6C protein | 20.42 | 21.61 | 20.12 |
| C01 | COX7A2 | COX7A2 protein | 20.85 | 21.6 | 20.89 |
| C02 | DNAJB1 | DnaJ (Hsp40) homolog, subfamily B, member 1 | 24.21 | 23.54 | 24.14 |
| C03 | EDN1 | Endothelin 1 | 30.08 | 28.14 | 30.65 |
| C04 | GADD45B | Growth arrest and DNA-damage-inducible, beta | 28.9 | 27.55 | 28.9 |
| C05 | HSP70.2 | Heat shock protein 70.2 | 25.59 | 23.46 | 24.54 |
| C06 | LHPP | Phospholysine phosphohistidine inorganic pyrophosphate phosphatase | 29.16 | 27.29 | 28.93 |
| C07 | LOC100154992 | Mitochondrial inner membrane protein OXA1L-like | 26 | 24.57 | 25.03 |
| C08 | LOC100156375 | COX15 homolog, cytochrome c oxidase assembly protein | 26.59 | 26.62 | 26.24 |
| C09 | LOC100156879 | Ubiquinol--cytochrome c reductase | 24.78 | 23.27 | 23.72 |
| C10 | LOC100156967 | Cytochrome c oxidase subunit 5A, mitochondrial-like | 20.37 | 20.47 | 20.09 |
| C11 | LOC100157935 | Cytochrome c oxidase subunit 8C, mitochondrial-like | 30.49 | 32.06 | 31.61 |
| C12 | LOC100511690 | Cytochrome c1, heme protein, mitochondrial-like | 23.36 | 22.24 | 22.37 |
| D01 | LOC100517408 | Cytochrome c oxidase subunit 7B2, mitochondrial-like | 34.44 | 37.93 | 34.27 |
| D02 | LOC100519366 | Cytochrome c oxidase subunit 6B1-like | 34.23 | 31.85 | 33.68 |
| D03 | LOC100519594 | Cytochrome c oxidase subunit 6A2, mitochondrial-like | 29.75 | 28.32 | 29.21 |
| D04 | LOC100522725 | Cytochrome c oxidase subunit 8A, mitochondrial-like | 23.84 | 23.41 | 23.6 |
| D05 | LOC100624067 | Cytochrome c oxidase subunit 4 isoform 1, mitochondrial-like | 20.77 | 20.62 | 20.6 |
| D06 | LOC100624950 | Cytochrome b561 domain-containing protein 1-like | 25.99 | 26.3 | 27.1 |
| D07 | LOC100739238 | Low-density lipoprotein receptor-related protein 5-like | 26.86 | 25.68 | 28.02 |
| D08 | NDUFB6 | NADH dehydrogenase 1 beta subcomplex 6 | 22.94 | 23.35 | 22.39 |
| D09 | ATP5O | ATP synthase H+-transporting mitochondrial F1 complex O subunit | 20.6 | 21.5 | 20.2 |
| D10 | NDUFA1 | NADH dehydrogenase [ubiquinone] 1 alpha subcomplex subunit 1-like | 22.72 | 23.17 | 23.04 |
| D11 | NDUFA10 | NADH dehydrogenase [ubiquinone] 1 alpha subcomplex subunit 10, mitochondrial-like | 25.44 | 24.5 | 24.83 |
| D12 | NDUFA11 | NADH dehydrogenase (ubiquinone) 1 alpha subcomplex, 11, 14.7kDa | 23.96 | 23.85 | 23.04 |
| E01 | NDUFA2 | NADH dehydrogenase (ubiquinone) 1 alpha subcomplex, 2, 8kDa | 26.33 | 26.66 | 25.42 |
| E02 | NDUFA3 | NADH dehydrogenase [ubiquinone] 1 alpha subcomplex subunit 3-like | 25.65 | 24.64 | 25.39 |
| E03 | NDUFA4 | NADH dehydrogenase (ubiquinone) 1 alpha subcomplex, 4, 9kDa | 20.43 | 21.34 | 20.51 |
| E04 | NDUFA5 | NADH dehydrogenase [ubiquinone] 1 alpha subcomplex subunit 5-like | 22.26 | 22.87 | 22.04 |
| E05 | NDUFA6 | NADH dehydrogenase (ubiquinone) 1 alpha subcomplex, 6, 14kDa | 31.04 | 29.41 | 31.05 |
| E06 | NDUFA7 | NADH dehydrogenase [ubiquinone] 1 alpha subcomplex subunit 7-like | 22.71 | 22.94 | 22.14 |
| E07 | NDUFA8 | NADH dehydrogenase (ubiquinone) 1 alpha subcomplex, 8, 19kDa | 23.31 | 23.65 | 22.73 |
| E08 | NDUFA9 | NADH dehydrogenase [ubiquinone] 1 alpha subcomplex subunit 9, mitochondrial-like | 35.74 | 35.19 | 34.35 |
| E09 | NDUFAB1 | NADH dehydrogenase (ubiquinone) 1, alpha/beta subcomplex, 1, 8kDa | 24.04 | 23.49 | 23.75 |
| E10 | NDUFB10 | NADH dehydrogenase [ubiquinone] 1 beta subcomplex subunit 10-like | 23.02 | 22.89 | 22.79 |
| E11 | NDUFB2 | NADH dehydrogenase (ubiquinone) 1 beta subcomplex, 2, 8kDa | 21.12 | 22.27 | 20.85 |
| E12 | NDUFB3 | NADH dehydrogenase (ubiquinone) 1 beta subcomplex, 3, 12kDa | 31.25 | 31.68 | 29.84 |
| F01 | NDUFB4 | NADH dehydrogenase [ubiquinone] 1 beta subcomplex subunit 4-like | 23.21 | 23.13 | 23.82 |
| F02 | NDUFB5 | NADH dehydrogenase (ubiquinone) 1 beta subcomplex, 5, 16kDa | 27.8 | 27.35 | 26.59 |
| F03 | NDUFB7 | NADH dehydrogenase (ubiquinone) 1 beta subcomplex, 7, 18kDa | 36.28 | 32.22 | 35.29 |
| F04 | NDUFB8 | NADH dehydrogenase (ubiquinone) 1 beta subcomplex, 8, 19kDa | 22.72 | 23.13 | 22.13 |
| F05 | NDUFB9 | NADH dehydrogenase (ubiquinone) 1 beta subcomplex, 9, 22kDa | 21.55 | 21.84 | 21.07 |
| F06 | NDUFC1 | NADH dehydrogenase (ubiquinone) 1, subcomplex unknown, 1, 6kDa | 22.63 | 23.67 | 22.48 |
| F07 | NDUFS1 | NADH dehydrogenase (ubiquinone) Fe-S protein 1, 75kDa (NADH-coenzyme Q reductase) | 26.51 | 26 | 25.45 |
| F08 | NDUFS2 | NADH dehydrogenase (ubiquinone) Fe-S protein 2, 49kDa (NADH-coenzyme Q reductase) | 33.45 | 31.08 | 33.3 |
| F09 | NDUFS3 | NADH dehydrogenase (ubiquinone) Fe-S protein 3, 30kDa (NADH-coenzyme Q reductase) | 24.1 | 23.24 | 23.31 |
| F10 | NDUFS4 | NADH dehydrogenase (ubiquinone) Fe-S protein 4, 18kDa (NADH-coenzyme Q reductase) | 22.56 | 22.92 | 22.03 |
| F11 | NDUFS5 | NADH dehydrogenase (ubiquinone) Fe-S protein 5, 15kDa (NADH-coenzyme Q reductase) | 21.18 | 22.03 | 20.63 |
| F12 | NDUFS6 | NADH dehydrogenase [ubiquinone] iron-sulfur protein 6, mitochondrial-like | 22.81 | 22.53 | 22.35 |
| G01 | NDUFS7 | NADH dehydrogenase [ubiquinone] iron-sulfur protein 7, mitochondrial-like | 22.39 | 22.17 | 22.68 |
| G02 | NDUFS8 | NADH dehydrogenase [ubiquinone] iron-sulfur protein 8, mitochondrial-like | 31.39 | 30.68 | 30.67 |
| G03 | NDUFV1 | NADH dehydrogenase [ubiquinone] flavoprotein 1, mitochondrial-like | 30.65 | 28.8 | 29.5 |
| G04 | PPA1 | Pyrophosphatase 1 | 21.45 | 21.63 | 20.42 |
| G05 | PPA2 | Inorganic pyrophosphatase 2, mitochondrial-like | 24.64 | 24.66 | 24.21 |
| G06 | SDHA | Succinate dehydrogenase [ubiquinone] flavoprotein subunit, mitochondrial | 22.95 | 22.15 | 22.65 |
| G07 | SDHB | Succinate dehydrogenase complex, subunit B, iron sulfur (Ip) | 22.4 | 22.37 | 21.75 |
| G08 | SDHC | Succinate dehydrogenase cytochrome b560 subunit, mitochondrial-like | 23.28 | 23.69 | 23.01 |
| G09 | SDHD | Succinate dehydrogenase complex, subunit D, integral membrane protein | 22.22 | 22.05 | 22.08 |
| G10 | SLC25A25 | Solute carrier family 25 (mitochondrial carrier; phosphate carrier), member 25 | 29.26 | 27.43 | 28.83 |
| G11 | UCP2 | Uncoupling protein 2 (mitochondrial, proton carrier) | 33.97 | 31 | 33.85 |
| G12 | UQCR10 | Ubiquinol-cytochrome c reductase complex 7.2 kDa protein | 24.08 | 24.7 | 23.04 |
| H01 | ACTG1 | Actin gamma 1 | 20.22 | 18.26 | 19.99 |
| H02 | B2M | Beta-2-microglobulin | 18.2 | 18.18 | 17.32 |
| H03 | GAPDH | Glyceraldehyde-3-phosphate dehydrogenase | 22.88 | 20.99 | 22.27 |
| H04 | HPRT1 | Hypoxanthine phosphoribosyltransferase 1 | 25.87 | 25.54 | 25.68 |
| H05 | RPL13A | Ribosomal protein L13a | 18.99 | 19.12 | 18.48 |
| H06 | SGDC | Pig Genomic DNA Contamination | 40 | 38.96 | 40 |
| H07 | RTC | Reverse Transcription Control | 22.47 | 22.44 | 22.47 |
| H08 | RTC | Reverse Transcription Control | 22.21 | 22.07 | 22.52 |
| H09 | RTC | Reverse Transcription Control | 22.58 | 22.09 | 22.47 |
| H10 | PPC | Positive PCR Control | 20.56 | 20.43 | 20.63 |
| H11 | PPC | Positive PCR Control | 20.49 | 20.39 | 20.6 |
| H12 | PPC | Positive PCR Control | 20.69 | 20.67 | 20.92 |

Normalization analysis used reference genes, B2M (Beta-2-microglobulin), GAPDH (Glyceraldehyde-3-phosphate dehydrogenase), HPRT1 (Hypoxanthine phosphoribosyltransferase 1), RPL13A (Ribosomal protein L13a), n = 3 piglets.

**S13 Table. RT² Profiler™ PCR Array Pig Cytokines and Chemokines in the intestinal mucosa of 14, 23 and 35 days old normal birth weight piglets**

| Well | Symbol | Description | D14 | D21 | D35 |
| --- | --- | --- | --- | --- | --- |
| A01 | ADIPOQ | Adiponectin. C1Q and collagen domain containing | 36.04 | 33.29 | 36.34 |
| A02 | AMCF-II | Alveolar macrophage-derived chemotactic factor-II | 27.52 | 26.39 | 25.68 |
| A03 | BMP2 | Bone morphogenetic protein 2 | 27.66 | 26.12 | 27.62 |
| A04 | BMP3 | Bone morphogenetic protein 3 | 27.43 | 25.95 | 28.4 |
| A05 | BMP4 | Bone morphogenetic protein 4 | 25.77 | 24.63 | 25.67 |
| A06 | BMP6 | Bone morphogenetic protein 6 | 33.6 | 32.45 | 34.76 |
| A07 | Bmp7 | Bone morphogenetic protein 7 | 31.29 | 29.42 | 31.21 |
| A08 | C5 | Complement component 5 | 25.27 | 23.38 | 23.59 |
| A09 | CCL1 | Chemokine (C-C motif) ligand 1 | 35.36 | 31.33 | 34.67 |
| A10 | CCL11 | CCL11 | 28.18 | 28.15 | 27.72 |
| A11 | CCL17 | Chemokine ligand 17-like protein | 33.67 | 27.84 | 32.64 |
| A12 | CCL19 | Chemokine (C-C motif) ligand 19 | 28.03 | 24.1 | 26.38 |
| B01 | CCL2 | Chemokine (C-C motif) ligand 2 | 25.64 | 23.85 | 24.91 |
| B02 | CCL20 | Chemokine (C-C motif) ligand 20 | 22.97 | 23.09 | 24.12 |
| B03 | CCL21 | Chemokine (C-C motif) ligand 21 | 26.47 | 25.31 | 27.64 |
| B04 | CCL22 | C-C motif chemokine 22-like | 37.11 | 32.95 | 37.33 |
| B05 | CCL25 | Chemokine (C-C motif) ligand 25 | 21.01 | 22.45 | 21.09 |
| B06 | CCL27 | Chemokine (C-C motif) ligand 27 | 30.76 | 28.97 | 29.77 |
| B07 | CCL28 | Chemokine (C-C motif) ligand 28 | 28.09 | 27.08 | 25.93 |
| B08 | CCL3L1 | Chemokine (C-C motif) ligand 3-like 1 | 31.73 | 27.73 | 29.47 |
| B09 | CCL4 | Chemokine (C-C motif) ligand 4 | 30.25 | 26.11 | 27.66 |
| B10 | CCL5 | Chemokine (C-C motif) ligand 5 | 24.97 | 24.08 | 24 |
| B11 | CCL8 | Chemokine (C-C motif) ligand 8 | 28.76 | 25.43 | 27.11 |
| B12 | CD40LG | CD40 ligand | 31.21 | 29.82 | 30.64 |
| C01 | CSF1 | Colony stimulating factor 1 (macrophage) | 31.13 | 28.44 | 30.42 |
| C02 | CSF2 | Colony stimulating factor 2 (granulocyte-macrophage) | 31.55 | 31.12 | 32.03 |
| C03 | CSF3 | Colony stimulating factor 3 (granulocyte) | 37.32 | 34.63 | 37.7 |
| C04 | CXCL10 | Chemokine (C-X-C motif) ligand 10 | 26.04 | 22.45 | 22.97 |
| C05 | CXCL11 | Chemokine (C-X-C motif) ligand 11 | 31.27 | 25.97 | 27.18 |
| C06 | CXCL12 | Chemokine (C-X-C motif) ligand 12 | 26.69 | 25.64 | 26.53 |
| C07 | LOC396594 | Growth-regulated protein homolog gamma | 28.29 | 27.91 | 28.46 |
| C08 | CXCL9 | Chemokine (C-X-C motif) ligand 9 | 29.19 | 24.86 | 25.76 |
| C09 | FASLG | Fas ligand (TNF superfamily. member 6) | 30.75 | 30.2 | 30.25 |
| C10 | IFN-ALPHA-4 | Interferon-alpha-4 | 31.75 | 30.18 | 32.79 |
| C11 | IFN-ALPHA-5 | Interferon. alpha 5 | 32.16 | 29.75 | 32.8 |
| C12 | IFNB1 | Interferon beta | 39.22 | 34.41 | 37.83 |
| D01 | IFNG | Interferon-gamma | 33.15 | 31 | 28.49 |
| D02 | IL10 | Interleukin 10 | 29.63 | 28.69 | 29.43 |
| D03 | IL12A | Interleukin 12A (natural killer cell stimulatory factor 1. cytotoxic lymphocyte maturation factor 1. p35) | 37.52 | 33.32 | 37.84 |
| D04 | IL12B | Interleukin 12B (natural killer cell stimulatory factor 2. cytotoxic lymphocyte maturation factor 2. p40) | 37 | 31 | 33.23 |
| D05 | IL13 | Interleukin 13 | 34.22 | 31.03 | 34.46 |
| D06 | IL15 | Interleukin 15 | 27.66 | 27.26 | 26.82 |
| D07 | IL16 | Interleukin 16 | 29.06 | 26.76 | 28.57 |
| D08 | IL17A | Interleukin 17A | 40 | 37.99 | 37.38 |
| D09 | IL17F | Interleukin 17F | 35.01 | 34.55 | 32.11 |
| D10 | IL18 | Interleukin 18 (interferon-gamma-inducing factor) | 24.58 | 23.35 | 23.11 |
| D11 | IL1A | Interleukin 1. alpha | 30.34 | 28.42 | 29.2 |
| D12 | IL1B1 | Interleukin 1. beta 1 | 31.29 | 28.52 | 30.45 |
| E01 | IL2 | Interleukin 2 | 38.56 | 32.93 | 33.92 |
| E02 | IL21 | Interleukin 21 | 40 | 33.34 | 31.56 |
| E03 | IL22 | Interleukin 22 | 34.5 | 33.49 | 30.76 |
| E04 | IL23A | Interleukin 23. alpha subunit p19 | 34.58 | 32.64 | 34.32 |
| E05 | IL27 | Interleukin 27 | 35.91 | 32.5 | 35.1 |
| E06 | IL4 | Interleukin 4 | 33.38 | 30.89 | 33.74 |
| E07 | IL5 | Interleukin 5 | 30.63 | 29.74 | 30.85 |
| E08 | IL6 | Interleukin 6 (interferon. beta 2) | 38.88 | 34.59 | 37.02 |
| E09 | IL7 | Interleukin 7 | 27.12 | 26.48 | 26.55 |
| E10 | CXCL8 | Interleukin 8 | 24.54 | 24.05 | 23.6 |
| E11 | IL9 | Interleukin 9 | 38.47 | 38 | 38.28 |
| E12 | INHA | Inhibin. alpha | 40 | 40 | 40 |
| F01 | INHBA | Inhibin. beta A | 30.3 | 29.35 | 31.48 |
| F02 | LIF | Leukemia inhibitory factor (cholinergic differentiation factor) | 31.19 | 29.31 | 31.29 |
| F03 | OSM | Oncostatin-M-like | 40 | 37.54 | 40 |
| F04 | LOC100515857 | C-C motif chemokine 3-like | 32.44 | 31.8 | 31.19 |
| F05 | CCL23 | C-C motif chemokine 23-like | 32.67 | 28.35 | 31.14 |
| F06 | CCL16 | C-C motif chemokine 16-like | 27.61 | 32.18 | 30.51 |
| F07 | CNTF | Ciliary neurotrophic factor-like | 30.43 | 29.2 | 31.21 |
| F08 | CXCL13 | C-X-C motif chemokine 13-like | 30.66 | 26.06 | 28.2 |
| F09 | THPO | Thrombopoietin | 35.3 | 33.66 | 33.84 |
| F10 | LOC100621682 | Uncharacterized LOC100621682 | 36.96 | 35.32 | 36.97 |
| F11 | TNFSF9 | Tumor necrosis factor ligand superfamily member 9-like | 32.15 | 30.21 | 31.41 |
| F12 | CCL24 | Chemokine ligand 24-like protein | 30.96 | 26.66 | 27.04 |
| G01 | LTA | Lymphotoxin alpha (TNF superfamily. member 1) | 36.08 | 30.62 | 36.85 |
| G02 | LTB | Lymphotoxin beta (TNF superfamily. member 3) | 29.85 | 25.48 | 28.84 |
| G03 | MIF | Macrophage migration inhibitory factor (glycosylation-inhibiting factor) | 21.61 | 20.63 | 21.51 |
| G04 | MSTN | Myostatin | 34.01 | 32.81 | 34.35 |
| G05 | SPP1 | Secreted phosphoprotein 1 | 30.37 | 22.28 | 26.51 |
| G06 | TGFB1 | Transforming growth factor. beta 1 | 27.46 | 24.64 | 27.09 |
| G07 | TGFB2 | Transforming growth factor. beta 2 | 29.2 | 28.69 | 30.26 |
| G08 | TNF | Tumor necrosis factor | 29.03 | 27.49 | 29.05 |
| G09 | TNFSF10 | Tumor necrosis factor (ligand) superfamily. member 10 | 24.11 | 23.23 | 22.99 |
| G10 | TNFSF13B | Tumor necrosis factor (ligand) superfamily. member 13b | 35.57 | 31.1 | 30.28 |
| G11 | TNFSF4 | Tumor necrosis factor (ligand) superfamily. member 4 | 31.87 | 30.32 | 31.77 |
| G12 | VEGFA | Vascular endothelial growth factor A | 26.52 | 25.44 | 27.03 |
| H01 | ACTG1 | Actin gamma 1 | 20.03 | 18.32 | 20.17 |
| H02 | B2M | Beta-2-microglobulin | 18.18 | 17.31 | 17.32 |
| H03 | GAPDH | Glyceraldehyde-3-phosphate dehydrogenase | 21.86 | 20.88 | 22.25 |
| H04 | HPRT1 | Hypoxanthine phosphoribosyltransferase 1 | 25.63 | 24.35 | 25.18 |
| H05 | RPL13A | Ribosomal protein L13a | 18.99 | 18.29 | 18.46 |
| H06 | SGDC | Pig Genomic DNA Contamination | 40 | 40 | 40 |
| H07 | RTC | Reverse Transcription Control | 22.74 | 21.91 | 22.41 |
| H08 | RTC | Reverse Transcription Control | 22.78 | 21.85 | 22.41 |
| H09 | RTC | Reverse Transcription Control | 22.72 | 21.8 | 22.41 |
| H10 | PPC | Positive PCR Control | 20.56 | 20.29 | 20.16 |
| H11 | PPC | Positive PCR Control | 20.58 | 20.25 | 20.13 |
| H12 | PPC | Positive PCR Control | 20.74 | 20.36 | 20.3 |

Normalization analysis used reference genes, B2M (Beta-2-microglobulin), GAPDH (Glyceraldehyde-3-phosphate dehydrogenase), HPRT1 (Hypoxanthine phosphoribosyltransferase 1), RPL13A (Ribosomal protein L13a), n = 3 piglets.

**S14 Table. RT² Profiler™ PCR Array Pig Apoptosis Ct values in the intestinal mucosa of 14, 23 and 35 days old normal birth weight piglets**

| Well | Symbol | Description | D14 | D23 | D35 |
| --- | --- | --- | --- | --- | --- |
| A01 | ABL1 | C-abl oncogene 1, non-receptor tyrosine kinase | 28.66 | 27.71 | 30.33 |
| A02 | AIFM1 | Apoptosis-inducing factor, mitochondrion-associated, 1 | 26.76 | 26.58 | 27.06 |
| A03 | AIFM3 | Apoptosis-inducing factor, mitochondrion-associated, 3 | 34.52 | 34.06 | 35.33 |
| A04 | AKT1 | V-akt murine thymoma viral oncogene homolog 1 | 24.99 | 24.05 | 25.9 |
| A05 | APAF1 | Apoptotic peptidase activating factor 1 | 26.02 | 26.12 | 27.4 |
| A06 | BAD | BCL2-associated agonist of cell death | 29.74 | 28.85 | 30.17 |
| A07 | BAG3 | BCL2-associated athanogene 3 | 28.88 | 29.38 | 31.63 |
| A08 | BAK1 | Bak protein | 27.22 | 26.61 | 27.49 |
| A09 | BAX | BCL2-associated X protein | 25.48 | 25.74 | 25.91 |
| A10 | BCL2 | B-cell CLL/lymphoma 2 | 28.78 | 27.43 | 29.16 |
| A11 | BCL2A1 | BCL2-related protein A1 | 28.6 | 26.07 | 27.05 |
| A12 | BCL2L1 | BCL2-like 1 | 26.37 | 25.76 | 26.59 |
| B01 | BCL2L10 | BCL2-like 10 (apoptosis facilitator) | 38.92 | 37.12 | 40 |
| B02 | LOC100154044 | BCL2-like 2 | 27.06 | 26.68 | 28.57 |
| B03 | BID | BH3 interacting domain death agonist | 24.39 | 24.38 | 24.96 |
| B04 | LOC100622859 | Baculoviral IAP repeat containing 2 | 26.43 | 26.18 | 26.93 |
| B05 | BIRC3 | Baculoviral IAP repeat containing 3 | 22.94 | 22.94 | 22.75 |
| B06 | BIRC5 | Baculoviral IAP repeat containing 5 | 25.65 | 25.24 | 24.77 |
| B07 | BNIP2 | BCL2/adenovirus E1B 19kDa interacting protein 2 | 31.55 | 31.45 | 31.75 |
| B08 | BNIP3L | BCL2/adenovirus E1B 19kDa interacting protein 3-like | 24.39 | 24.9 | 25.06 |
| B09 | BRAF | V-raf murine sarcoma viral oncogene homolog B1 | 27.26 | 27.06 | 27.99 |
| B10 | CASP1 | Caspase 1, apoptosis-related cysteine peptidase (interleukin 1, beta, convertase) | 24.19 | 24.3 | 23.14 |
| B11 | CASP10 | Caspase 10, apoptosis-related cysteine peptidase | 23.69 | 23.83 | 24.74 |
| B12 | CASP14 | Caspase 14, apoptosis-related cysteine peptidase | 40 | 37.85 | 40 |
| C01 | CASP2 | Caspase 2, apoptosis-related cysteine peptidase | 26.52 | 25.16 | 26.86 |
| C02 | CASP3 | Caspase 3, apoptosis-related cysteine peptidase | 21.88 | 22.35 | 22.22 |
| C03 | LOC100522887 | Caspase 4, apoptosis-related cysteine peptidase | 23.88 | 23.79 | 23.33 |
| C04 | CASP6 | Caspase 6, apoptosis-related cysteine peptidase | 23.01 | 23.15 | 22.94 |
| C05 | CASP8 | Caspase 8, apoptosis-related cysteine peptidase | 26 | 26.24 | 25.7 |
| C06 | CBX4 | Chromobox homolog 4 | 28.7 | 28.16 | 30.08 |
| C07 | CD40 | CD40 molecule, TNF receptor superfamily member 5 | 29.47 | 27.07 | 29.05 |
| C08 | CD40LG | CD40 ligand | 30.54 | 29.95 | 30.63 |
| C09 | CD70 | CD70 molecule | 35.35 | 32.4 | 34.36 |
| C10 | CFDP1 | Craniofacial development protein 1 | 24.78 | 24.68 | 25.19 |
| C11 | CIDEA | Cell death-inducing DFFA-like effector a | 36.45 | 36.73 | 37.29 |
| C12 | CIDEB | Cell-death-inducing DNA-fragmentation-factor-like effector B | 22.69 | 22.59 | 24.09 |
| D01 | CUL2 | Cullin 2 | 27.07 | 26.71 | 26.84 |
| D02 | CYCS | Cytochrome c, somatic | 22.87 | 23.34 | 23.4 |
| D03 | LOC100516103 | Death-associated protein kinase 1 | 28.71 | 26.81 | 29.29 |
| D04 | DDX20 | DEAD (Asp-Glu-Ala-Asp) box polypeptide 20 | 26.77 | 25.92 | 27.44 |
| D05 | DIABLO | Diablo, IAP-binding mitochondrial protein | 25.13 | 25.24 | 25.17 |
| D06 | DPF2 | D4, zinc and double PHD fingers family 2 | 26.21 | 25.19 | 26.27 |
| D07 | ERC1 | ELKS/RAB6-interacting/CAST family member 1 | 27.89 | 27.91 | 30.23 |
| D08 | ERN2 | Endoplasmic reticulum to nucleus signaling 2 | 30.34 | 29.31 | 31.06 |
| D09 | FAS | Fas (TNF receptor superfamily, member 6) | 27.74 | 26.62 | 26.93 |
| D10 | FASLG | Fas ligand (TNF superfamily, member 6) | 30.76 | 30.79 | 30.09 |
| D11 | FEM1B | Fem-1 homolog b (C. elegans) | 26.45 | 26.28 | 27.31 |
| D12 | GADD45A | Growth arrest and DNA-damage-inducible, alpha | 23.97 | 24.08 | 24.52 |
| E01 | IGF1R | Insulin-like growth factor 1 receptor | 27.67 | 26.98 | 29.97 |
| E02 | IL10 | Interleukin 10 | 29.17 | 30 | 29.16 |
| E03 | LALBA | Lactalbumin, alpha- | 38.73 | 40 | 40 |
| E04 | HRK | Activator of apoptosis harakiri-like | 33.96 | 34 | 36.17 |
| E05 | LOC100156777 | Caspase 7 | 22.96 | 22.82 | 22.92 |
| E06 | TNFRSF21 | Tumor necrosis factor receptor superfamily member 21-like | 24.66 | 24.05 | 25.58 |
| E07 | RIPK2 | Receptor-interacting serine/threonine-protein kinase 2-like | 26.73 | 27.17 | 27.03 |
| E08 | CASP9 | Caspase-9-like | 30.92 | 30.33 | 31.89 |
| E09 | LTBR | Tumor necrosis factor receptor superfamily member 3-like | 28.09 | 27.32 | 28.54 |
| E10 | LOC100522011 | Apoptosis-associated speck-like protein containing a CARD-like | 23.13 | 23.47 | 22.07 |
| E11 | DFFA | DNA fragmentation factor subunit alpha-like | 26.8 | 26.67 | 27.37 |
| E12 | LOC100523672 | Nucleolar protein 3-like | 31.13 | 31.32 | 31.98 |
| F01 | BNIP3 | BCL2/adenovirus E1B 19 kDa protein-interacting protein 3-like | 29.7 | 30 | 31.37 |
| F02 | LOC641352 | Caspase-15 | 27.42 | 26.42 | 28.23 |
| F03 | LTA | Lymphotoxin alpha (TNF superfamily, member 1) | 35.3 | 31.8 | 37.35 |
| F04 | MCL1 | Myeloid cell leukemia sequence 1 (BCL2-related) | 23.41 | 23.2 | 23.53 |
| F05 | MTL5 | Metallothionein-like 5, testis-specific (tesmin) | 33.04 | 31.11 | 31.88 |
| F06 | NFKB1 | Nuclear factor of kappa light polypeptide gene enhancer in B-cells 1 | 26.49 | 25.25 | 26.67 |
| F07 | NOD1 | Nucleotide-binding oligomerization domain containing 1 | 35.87 | 33.13 | 35.64 |
| F08 | PAK7 | P21 protein (Cdc42/Rac)-activated kinase 7 | 37.1 | 39.53 | 40 |
| F09 | PPP2R1A | Protein phosphatase 2, regulatory subunit A, alpha | 29.15 | 27.94 | 30.62 |
| F10 | PPP2R1B | Protein phosphatase 2, regulatory subunit A, beta | 25.93 | 25.86 | 26.72 |
| F11 | PROP1 | PROP paired-like homeobox 1 | 39.41 | 38.88 | 39.54 |
| F12 | RFWD2 | Ring finger and WD repeat domain 2 | 26.57 | 26.51 | 27.16 |
| G01 | SART1 | Squamous cell carcinoma antigen recognized by T cells | 28.64 | 27.29 | 29.62 |
| G02 | LOC100517325 | Signal-induced proliferation-associated 1 | 33.02 | 30.97 | 33.97 |
| G03 | STAMBP | STAM binding protein | 25.09 | 25.19 | 25.52 |
| G04 | TNF | Tumor necrosis factor | 28.34 | 27.52 | 29.32 |
| G05 | TNFRSF11B | Tumor necrosis factor receptor superfamily, member 11b | 32.74 | 31 | 33.14 |
| G06 | TNFRSF1A | Tumor necrosis factor receptor superfamily, member 1A | 29.66 | 29.08 | 30.47 |
| G07 | TNFRSF1B | Tumor necrosis factor receptor superfamily, member 1B | 28.69 | 26.41 | 28.9 |
| G08 | TNFSF10 | Tumor necrosis factor (ligand) superfamily, member 10 | 23.57 | 24 | 23.27 |
| G09 | TP53 | Tumor protein p53 | 25.06 | 24.04 | 25.18 |
| G10 | TP53BP2 | Tumor protein p53 binding protein, 2 | 27.43 | 26.6 | 28.2 |
| G11 | TRAF2 | TNF receptor-associated factor 2 | 29.29 | 27.93 | 29.96 |
| G12 | XIAP | X-linked inhibitor of apoptosis | 24.46 | 24.55 | 24.69 |
| H01 | ACTG1 | Actin gamma 1 | 19.6 | 18.58 | 20.56 |
| H02 | B2M | Beta-2-microglobulin | 18.02 | 18.23 | 17.62 |
| H03 | GAPDH | Glyceraldehyde-3-phosphate dehydrogenase | 21.43 | 20.9 | 22.39 |
| H04 | HPRT1 | Hypoxanthine phosphoribosyltransferase 1 | 25.39 | 25.17 | 25.56 |
| H05 | RPL13A | Ribosomal protein L13a | 18.88 | 18.82 | 18.39 |
| H06 | SGDC | Pig Genomic DNA Contamination | 40 | 38.81 | 40 |
| H07 | RTC | Reverse Transcription Control | 22.18 | 22.46 | 22.41 |
| H08 | RTC | Reverse Transcription Control | 22.25 | 22.44 | 22.42 |
| H09 | RTC | Reverse Transcription Control | 22.18 | 22.51 | 22.47 |
| H10 | PPC | Positive PCR Control | 19.91 | 20.04 | 19.98 |
| H11 | PPC | Positive PCR Control | 19.95 | 20.09 | 20.01 |
| H12 | PPC | Positive PCR Control | 20 | 20.16 | 20.16 |

Normalization analysis used reference genes, B2M (Beta-2-microglobulin), GAPDH (Glyceraldehyde-3-phosphate dehydrogenase), HPRT1 (Hypoxanthine phosphoribosyltransferase 1), RPL13A (Ribosomal protein L13a), n = 3 piglets.

**S15 Table. Analysis of variance (main effects and interactions) for all measured parameters in qPCR and ELISA analyses of intestinal mucosa liver, kidney and plasma of piglets (P-values).**

| Gene | *P-values* | | |
| --- | --- | --- | --- |
|  | **Birthweight** | **Age Birthweight x Age** | |
| *Mucosa* |  |  |  |
| *BCL2A1* | 0.05 | <.0001 | 0.94 |
| *CCL19* | 0.26 | <.0001 | 0.44 |
| *GPX2* | 0.89 | <.0001 | 0.36 |
| *IL8* | 0.08 | 0.005 | 0.69 |
| *MT3* | 0.48 | <.0001 | 0.18 |
| *NCF1* | 0.21 | 0.11 | 0.76 |
| *NDUFB2* | 0.09 | 0.002 | 0.17 |
| *NDUFB7* | 0.50 | 0.004 | 0.44 |
| *TXNRD2* | 0.006 | 0.02 | 0.58 |
| Liver |  |  |  |
| *BNIP3* | <.0001 | 0.76 | 0.26 |
| *GPX3* | 0.002 | 0.009 | 0.65 |
| *HMOX1* | 0.07 | 0.007 | 0.74 |
| *MSRA* | 0.95 | 0.0005 | 0.20 |
| *MT3* | 0.47 | <.0001 | 0.27 |
| *NDUFA2* | 0.43 | <.0001 | 0.17 |
| *NDUFA5* | 0.45 | 0.02 | 0.98 |
| *PRDX3* | 0.47 | <.0001 | 0.82 |
| *TXNRD2* | 0.012 | 0.001 | 0.41 |
| Kidney |  |  |  |
| *BNIP3* | 0.70 | 0.001 | 0.06 |
| *GPx1* | 0.58 | 0.55 | 0.73 |
| *GPx3* | 0.008 | <.0001 | 0.01 |
| *MRSA* | 0.21 | 0.57 | 0.47 |
| *MT1* | 0.53 | <.0001 | 0.51 |
| *MT2* | 0.61 | <.0001 | 0.81 |
| *NDUFA2* | 0.53 | 0.0002 | 0.30 |
| *NDUFA5* | 0.58 | 0.003 | 0.51 |
| *TXN* | 0.91 | 0.03 | 1.00 |
| Plasma |  |  |  |
| *TNF-* *α* | 0.06 | <.0001 | 0.34 |
| *IL1-* *β* | 0.01 | <.0001 | 0.27 |

**S16 Table. Analysis of variance (main effects and interactions) with both litter and gender included as randow factors for all measured parameters in qPCR and ELISA analyses of intestinal mucosa liver, kidney and plasma of piglets (P-values).**

| Gene | *P-values* | | |
| --- | --- | --- | --- |
|  | **Birthweight** | **Age Birthweight x Age** | |
| *Mucosa* |  |  |  |
| *BCL2A1* | 0.05 | <.0001 | 0.94 |
| *CCL19* | 0.16 | <.0001 | 0.33 |
| *GPX2* | 0.88 | <.0001 | 0.36 |
| *IL8* | 0.09 | 0.006 | 0.68 |
| *MT3* | 0.83 | <.0001 | 0.25 |
| *NCF1* | 0.31 | 0.08 | 0.91 |
| *NDUFB2* | 0.12 | 0.002 | 0.15 |
| *NDUFB7* | 0.59 | 0.005 | 0.45 |
| *TXNRD2* | 0.005 | 0.04 | 0.46 |
| Liver |  |  |  |
| *BNIP3* | <.0001 | 0.76 | 0.27 |
| *GPX3* | 0.004 | 0.02 | 0.66 |
| *HMOX1* | 0.07 | 0.008 | 0.74 |
| *MSRA* | 0.97 | 0.0005 | 0.18 |
| *MT3* | 0.75 | <.0001 | 0.31 |
| *NDUFA2* | 0.43 | <.0001 | 0.17 |
| *NDUFA5* | 0.58 | 0.02 | 0.96 |
| *PRDX3* | 0.47 | <.0001 | 0.82 |
| *TXNRD2* | 0.012 | 0.0009 | 0.40 |
| Kidney |  |  |  |
| *BNIP3* | 0.71 | 0.001 | 0.06 |
| *GPx1* | 0.67 | 0.69 | 0.60 |
| *GPx3* | 0.008 | <.0001 | 0.01 |
| *MRSA* | 0.20 | 0.55 | 0.45 |
| *MT1* | 0.61 | <.0001 | 0.51 |
| *MT2* | 0.56 | <.0001 | 0.74 |
| *NDUFA2* | 0.70 | 0.0002 | 0.31 |
| *NDUFA5* | 0.87 | 0.003 | 0.50 |
| *TXN* | 0.98 | 0.06 | 0.99 |
| Plasma |  |  |  |
| *TNF-* *α* | 0.02 | 0.0009 | 0.45 |
| *IL1-* *β* | 0.0007 | 0.001 | 0.47 |

**S2 Fig. Box plot of targeted gene expression measured by quantitative PCR in liver of low and normal birthweight piglets at specific days of age.**

**
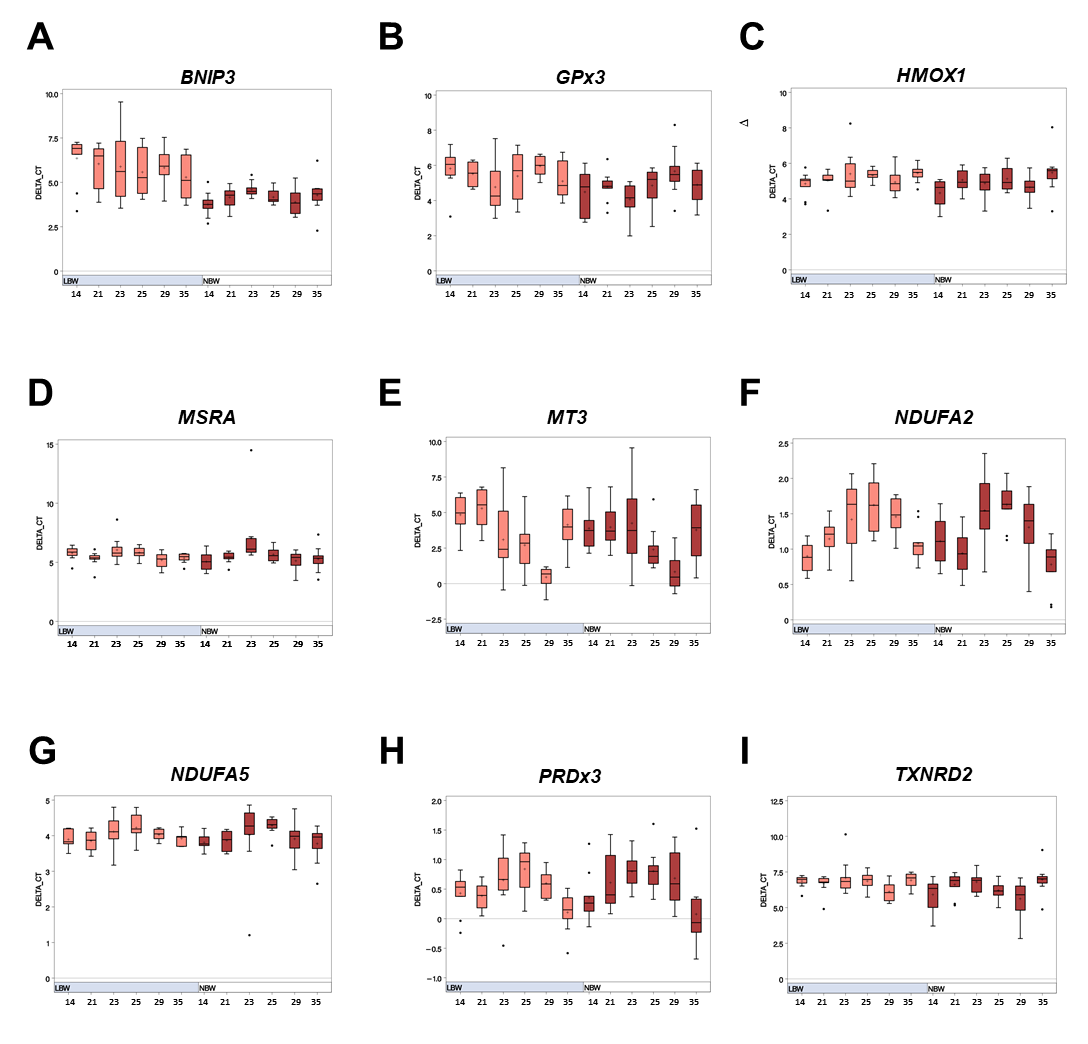
**Box plot of mRNA expression (delta CT) of LBW (low-birth weight piglets) and NBW (normal-birth weight piglets), n = 10, at specific days of age. *BNIP3 = BCL2 interacting protein 3; GPx3 = glutathione peroxidase 3; HMOX1 = heme oxygenase 1; MRSA = methionine sulfoxide reductase A, MT3 = metallothionein 3; NDUFA2 = NADH ubiquinone oxidoreductase subunit A2; NDUFA5 = NADH ubiquinone oxidoreductase subunit A5; PRDx3 = peroxideroxin 3;TXNRD2 = thioredoxin reductase 2.*

**S3 Fig. Box plot of targeted gene expression measured by quantitative PCR in intestinal mucosa of low and normal birthweight piglets at specific days of age.
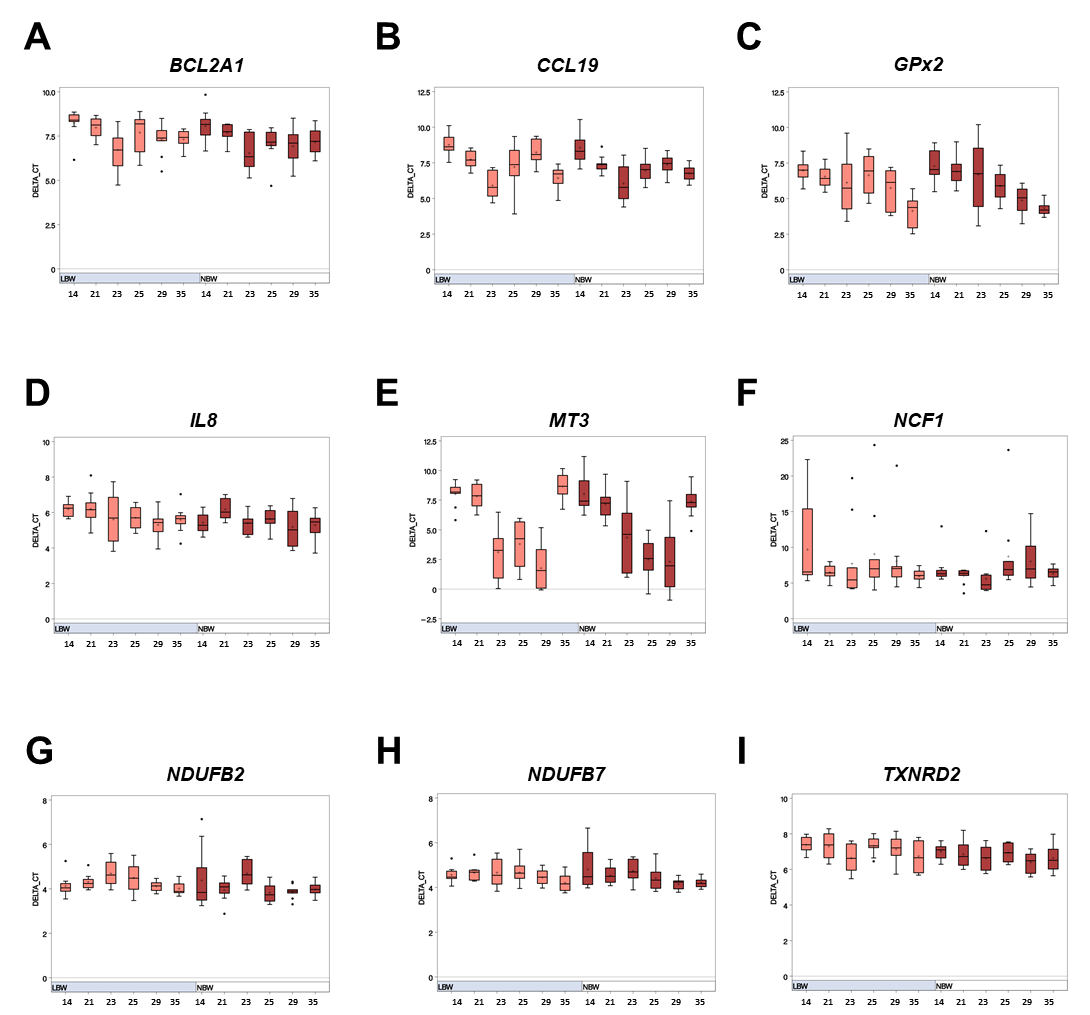
**

Box plot of mRNA expression (delta CT) of LBW (low-birth weight piglets) and NBW (normal-birth weight piglets), n = 10, at specific days of age . *BCL2A1 = B-cell lymphoma 2-related protein A1; CCL19 = c-c motif chemokine ligand 19; GPx2 = glutathione peroxidase 2; IL8 = interleukin 8; MT3 = metallothionein 3; NCF1= neutrophil cytosol factor 1; NDUFB2 = NADH ubiquinone oxidoreductase subunit B2; NDUFB7 = NADH ubiquinone oxidoreductase subunit B7; TXNRD2 = thioredoxin reductase 2.*

**S4 Fig. Box plot of targeted gene expression measured by quantitative PCR in kidney of low and normal birthweight piglets at specific days of age.**

**
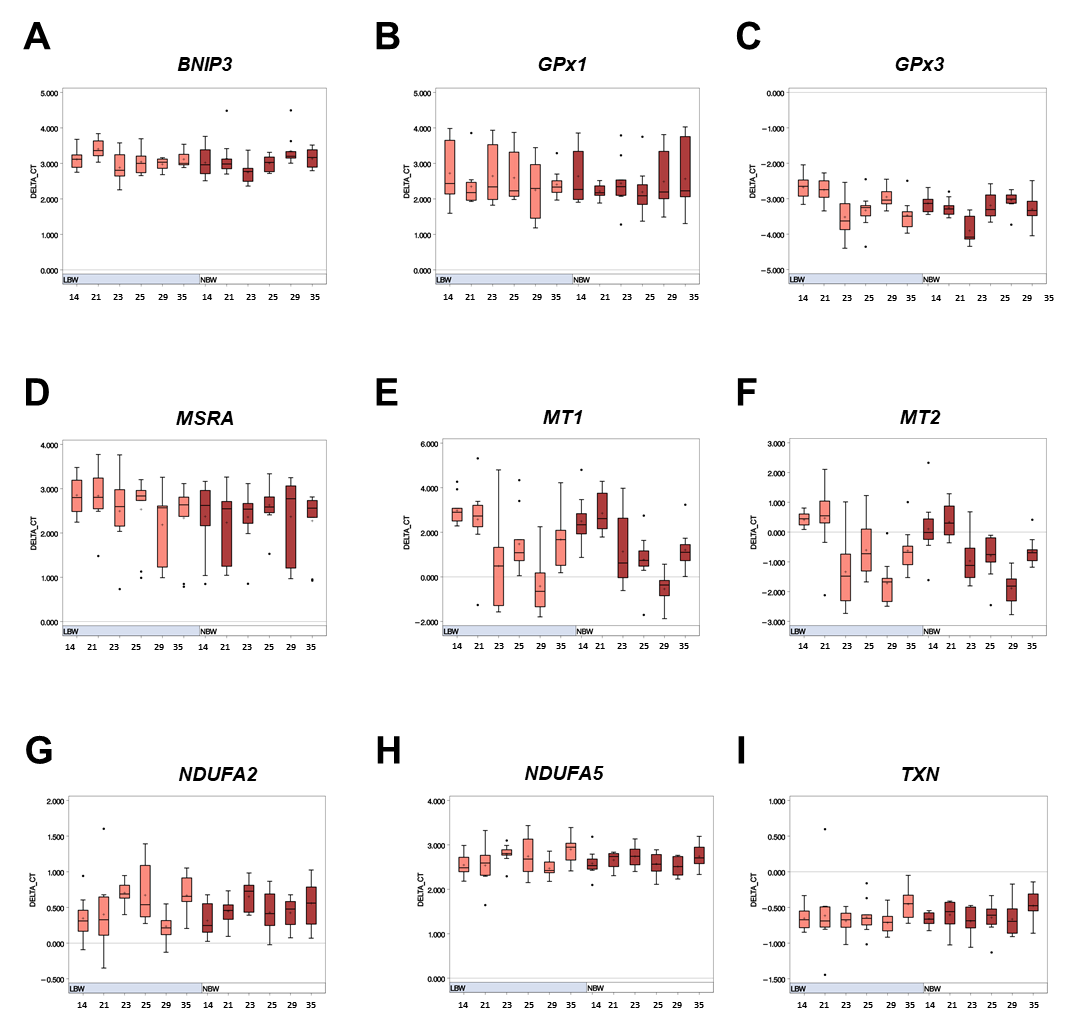
**

Box plot of mRNA expression (delta CT) of LBW (low-birth weight piglets) and NBW (normal-birth weight piglets), n = 10, at specific days of age. *BNIP3* = BCL2 interacting protein 3; *GPx1* = glutathione peroxidase 1; *GPx3* = glutathione peroxidase 3; *MRSA*= methionine sulfoxide reductase A; *MT1* = metallothionein 1A; *MT2* = metallothionein 2B; *NDUFA2* = NADH ubiquinone oxidoreductase subunit A2; *NDUFA5* = NADH ubiquinone oxidoreductase subunit A5; TXN = thioredoxin

**S5 Fig. Box plot of levels of TNF-α and IL-1β in plasma samples** **of LBW and NBW piglets at specific days of age throughout the peri-weaning period.**


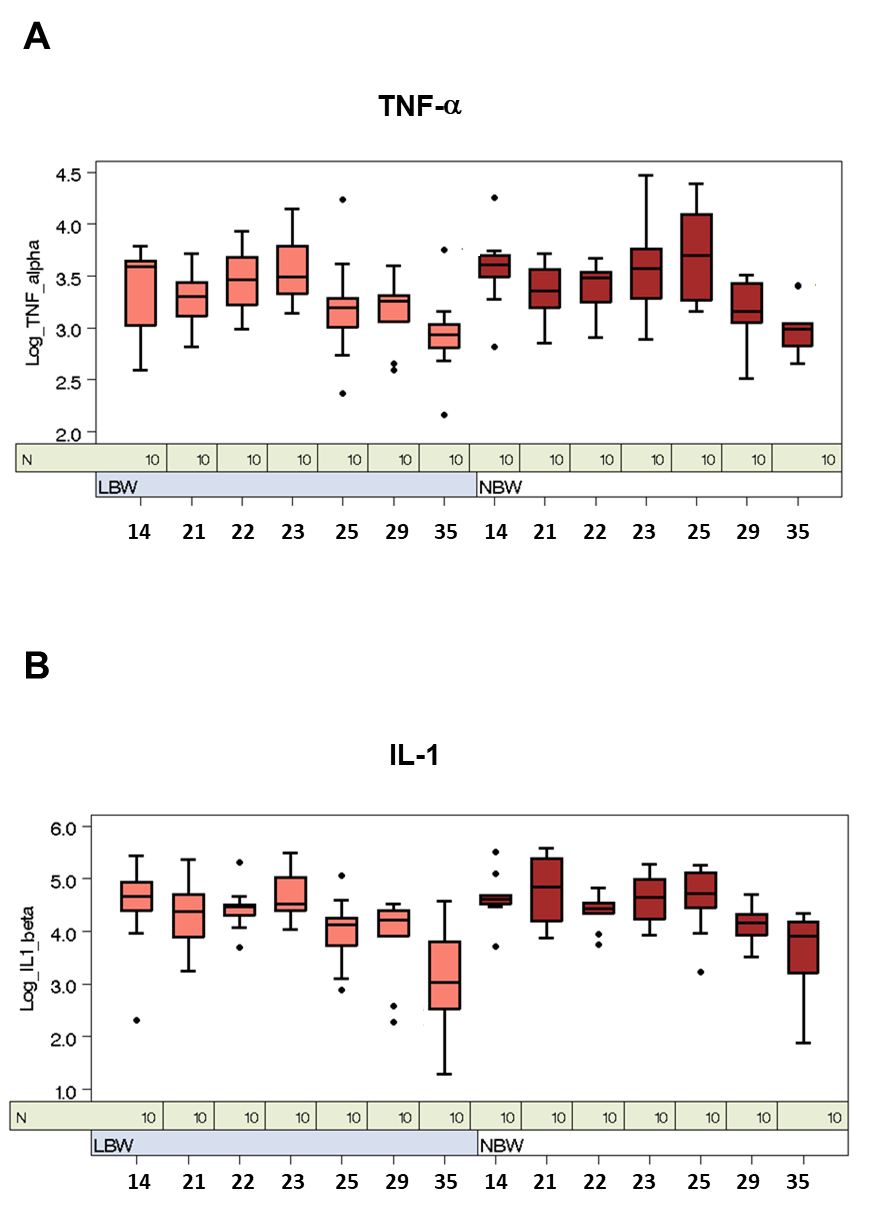

Supplement: S1 File — (DOCX) [file pone.0247188.s001.docx]
